# Full Spectroscopic Characterization of the Molecular Oxygen-Based Methane to Methanol Conversion over Open Fe(II) Sites in a Metal–Organic Framework

**DOI:** 10.1021/jacs.3c07216

**Published:** 2023-09-18

**Authors:** Alessandro Tofoni, Francesco Tavani, Marco Vandone, Luca Braglia, Elisa Borfecchia, Paolo Ghigna, Dragos Costantin Stoian, Toni Grell, Sara Stolfi, Valentina Colombo, Paola D’Angelo

**Affiliations:** † Dipartimento di Chimica, Università degli Studi di Roma “La Sapienza”, P.le A. Moro 5, I-00185 Rome, Italy; ‡ Dipartimento di Chimica & UdR INSTM di Milano, Università degli Studi di Milano, Via Golgi 19, 20133 Milan, Italy; § CNR-Istituto Officina dei Materiali, TASC, 34149 Trieste, Italy; ∥ Dipartimento di Chimica & UdR INSTM di Torino, Università di Torino, Via P. Giuria 7, 10125 Turin, Italy; ⊥ Dipartimento di Chimica, Università di Pavia, V.le Taramelli 13, I-27100 Pavia, Italy; # The Swiss-Norwegian Beamlines (SNBL), European Synchrotron Radiation Facility, BP 220, 38043 Grenoble, France; ∇ CNR − SCITEC − Istituto di Scienze e Tecnologie Chimiche “Giulio Natta”, Via Golgi 19, 20133 Milan, Italy

## Abstract

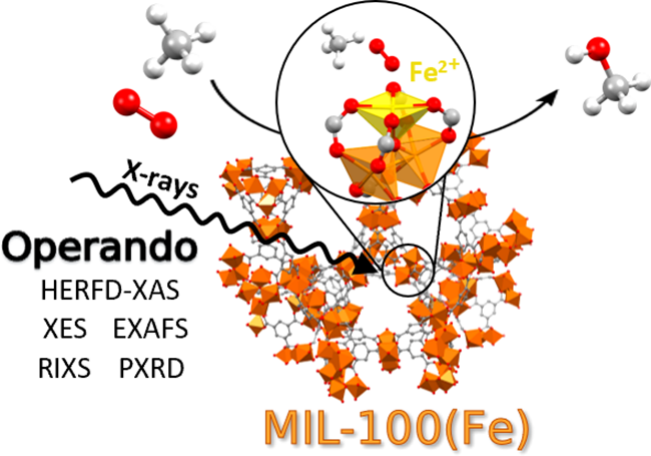

Iron-based enzymes efficiently activate molecular oxygen to perform the oxidation of methane to methanol (MTM), a reaction central to the contemporary chemical industry. Conversely, a very limited number of artificial catalysts have been devised to mimic this process. Herein, we employ the MIL-100(Fe) metal–organic framework (MOF), a material that exhibits isolated Fe sites, to accomplish the MTM conversion using O_2_ as the oxidant under mild conditions. We apply a diverse set of advanced operando X-ray techniques to unveil how MIL-100(Fe) can act as a catalyst for direct MTM conversion. Single-phase crystallinity and stability of the MOF under reaction conditions (200 or 100 °C, CH_4_ + O_2_) are confirmed by X-ray diffraction measurements. X-ray absorption, emission, and resonant inelastic scattering measurements show that thermal treatment above 200 °C generates Fe(II) sites that interact with O_2_ and CH_4_ to produce methanol. Experimental evidence-driven density functional theory (DFT) calculations illustrate that the MTM reaction involves the oxidation of the Fe(II) sites to Fe(III) via a high-spin Fe(IV)=O intermediate. Catalyst deactivation is proposed to be caused by the escape of CH_3_^•^ radicals from the relatively large MOF pore cages, ultimately resulting in the formation of hydroxylated triiron units, as proven by valence-to-core X-ray emission spectroscopy. The O_2_-based MTM catalytic activity of MIL-100(Fe) in the investigated conditions is demonstrated for two consecutive reaction cycles, proving the MOF potential toward active site regeneration. These findings will desirably lay the groundwork for the design of improved MOF catalysts for the MTM conversion.

## Introduction

The direct oxidation of methane to methanol (MTM) is a grand challenge in modern catalysis with profound implications for the chemical industry.^1^ In order to activate the strongly inert methane C–H bond, current large-scale technologies rely on elevated temperatures and pressures (*T* > 400 °C, *P* > 30 bar), involving expensive and nonenvironmentally friendly processes.^2−5^ Nature masters the selective and efficient MTM conversion at ambient conditions using enzymes, such as soluble methane monooxygenase (sMMO) which is based on a diiron core.^6^ This highlights the key role played by active sites containing transition metal (TM) ions such as iron in enabling the efficient catalytic MTM conversion and has inspired the development of biomimetic TM catalysts.^7^ Effective MTM catalysts are required to activate the inert methane C–H bond under mild conditions (*T* < 300 °C, 1 atm), while preventing the overoxidation of the methanol product.^8^

Metal–organic frameworks (MOFs) are porous hybrid materials that are emerging as novel platforms for C–H bond activation reactions, and their exceptional tunability makes them ideal candidates to catalyze the MTM conversion.^9,10^ In recent years, iron-based MOFs have attracted considerable attention for the MTM oxidation, conducted in most cases employing N_2_O as the oxidant.^7,11−19^ However, limited availability and high cost make the use of N_2_O inconvenient for practical applications.^20^ Nitrous oxide is also a neurotoxic gas with long-term exposure hazards,^21^ for which an exposure limit of 25 ppm has been established by the National Institute for Occupational Safety and Health (NIOSH). Conversely, employing molecular oxygen for the MTM conversion is of enormous interest since it would represent a more industrially relevant and economically favorable oxidant.^22,23^ The catalytic dissociation of O_2_ is widespread in enzymatic systems,^24−26^ but a very limited number of artificial catalysts have been devised to mimic this process.^17,27−29^ Specifically, the use of O_2_ for the Fe-MOF-catalyzed MTM reaction has not, to the best of our knowledge, yet been carried out under nonphotocatalytic conditions.^30^ The MTM conversion employing O_2_ has recently been performed using a ferrierite zeolite.^22^ In this system, it was proposed that the active Fe(IV)=O complex is formed when O_2_ dissociates over adjacent Fe(II) sites, in analogy to the putative mechanism of O_2_ cleavage at the sMMO diiron catalytic core.^24,31^ As a consequence, it appears of significant interest to investigate the MTM conversion over Fe-based MOFs employing O_2_ with molecular-level accuracy provided by spectroscopic and diffraction techniques.

MIL-100(Fe) is a MOF composed of Fe(III) trimers linked through the benzene-1,3,5-tricarboxylate (BTC) ligand, whose thermal treatment above 200 °C generates high-spin (S = 2) Fe(II) coordinatively unsaturated sites (CUSs).^32,33^ In recent years it has been demonstrated, both from theoretical^34,35^ and experimental^36,37^ points of view, that such Fe(II) sites enable the partial oxidation of light alkanes, such as methane, ethane and propane, to the respective alcohols under mild conditions using N_2_O. Conversely, no attempts have been reported in the literature to employ molecular oxygen to carry out the MIL-100(Fe) catalyzed MTM conversion. Notably, the iron species in this MOF have similar geometry and oxidation state as those proposed for Fe(II)-based heme and nonheme enzymes known to dissociate O_2_ to form Fe(IV)=O complexes,^38−40^ supporting the hypothesis that these sites may activate molecular oxygen for the MTM reaction as well.

In this work, we apply a diverse set of advanced operando X-ray techniques to unveil how MIL-100(Fe) shows a promising reactivity toward the direct MTM conversion under mild conditions using O_2_ as the oxidant. We employ operando X-ray absorption (XAS) and emission (XES) spectroscopies together with resonant inelastic X-ray scattering (RIXS) and powder X-ray diffraction (PXRD) measurements to fully characterize the MIL-100(Fe) MOF during activation and under conditions relevant to the O_2_-based MTM reaction. A structural and mechanistic understanding of the reactive MTM process is reached by multivariate and density functional theory (DFT) analyses of the experimental data. We initially track the formation of unsaturated Fe(II) sites upon thermal treatment with a thorough operando spectroscopic analysis. We then provide the first evidence of MTM conversion using O_2_ under mild conditions over MIL-100(Fe), and exploit the complementarity of the employed experimental techniques to propose a consistent chemical assignment for the key reaction species. Finally, we employ state-of-the-art theoretical simulations of the spectroscopic data to validate the experimental results, which are then used as a basis to propose a DFT-derived mechanistic picture that is fully coherent with the experimental evidence.

## Experimental and Computational Methods

### Synthesis and Characterization of MIL-100(Fe)

MIL-100(Fe) was synthesized in gram quantities following a HF-free procedure based on water reconstruction, with slight modifications to the previously reported protocol.^41^ PXRD patterns, reported in Figure S1, show improved crystallinity of the sample at the end of the reconstruction procedure, and a structureless Le Bail refinement (Figure S2) confirmed the phase purity of the material. The measured attenuated total reflectance Fourier transform IR (ATR-FTIR) spectra (Figure S6) as well as thermogravimetric analysis (TGA) and N_2_ sorption profiles (Figure S7a,b, respectively) are in agreement with those reported in the literature.^33^ The thermal behavior of MIL100-(Fe) was investigated prior to the operando experiments with in-house variable-temperature PXRD (VT-PXRD) measurements. All structureless Le Bail refinements were carried out using the TOPAS-Academic 6 software package.^42−44^ Full details on the synthesis and characterization of MIL-100(Fe) are provided in Section S1 of the SI.

### Operando HERFD-XAS, XAS-PXRD, XES, and RIXS Experiments

Operando high energy resolution fluorescence detected (HERFD) XAS, 1s3p RIXS and valence-to-core (VtC) XES measurements were performed at beamline ID26 of the European Synchrotron Radiation Facility (ESRF).^45^ HERFD-XAS spectra were collected in an energy range between 7100 and 7150 eV in 0.1 eV steps. Resonant Kβ and VtC XES measurements were collected in energy ranges of 7030–7068 and 7060–7120 eV, respectively, in 0.2 eV steps.

Combined operando XAS and PXRD measurements were performed at the BM31 beamline of the ESRF. XAS spectra were collected between 7000 and 7900 eV with a step of 0.3 eV. PXRD patterns were instead collected with a 2D DEXELA detector using a wavelength of 0.3386 Å.^46^ Full details on the operando HERFD-XAS, XAS-PXRD, and XES and RIXS measurements are provided in Section S1 of the SI.

### *Ab Initio* XAS and VtC-XES Calculations

The XANES and VtC-XES theoretical data analysis was performed with the FDMNES code using the finite differences method (FDM) implementing the solver method for the finite difference matrix.^47−49^ Simulations of XANES and VtC-XES spectra were performed including spin–orbit coupling, quadrupolar transitions, and self-consistency in the calculations.^50^ A Gaussian broadening of 2.5 eV was applied to the VtC-XES theoretical spectra. Additional details on the calculations are provided in Section S2 of the SI.

### DFT Calculations

DFT calculations were carried out with the ORCA 5.0.2 quantum chemistry code using the uM06-L functional together with the def2-TZVP basis set.^51−55^ This combination of functional and basis set was employed following a widespread and previously benchmarked approach to model the reactivity of trimeric nodes found in MIL-100(Fe), where the BTC ligands are substituted by formyl groups.^16,34−36,56^ Further details are reported in Section S3 of the SI.

## Results and Discussion

### Thermal Activation of MIL-100(Fe)

**Figure 1:**
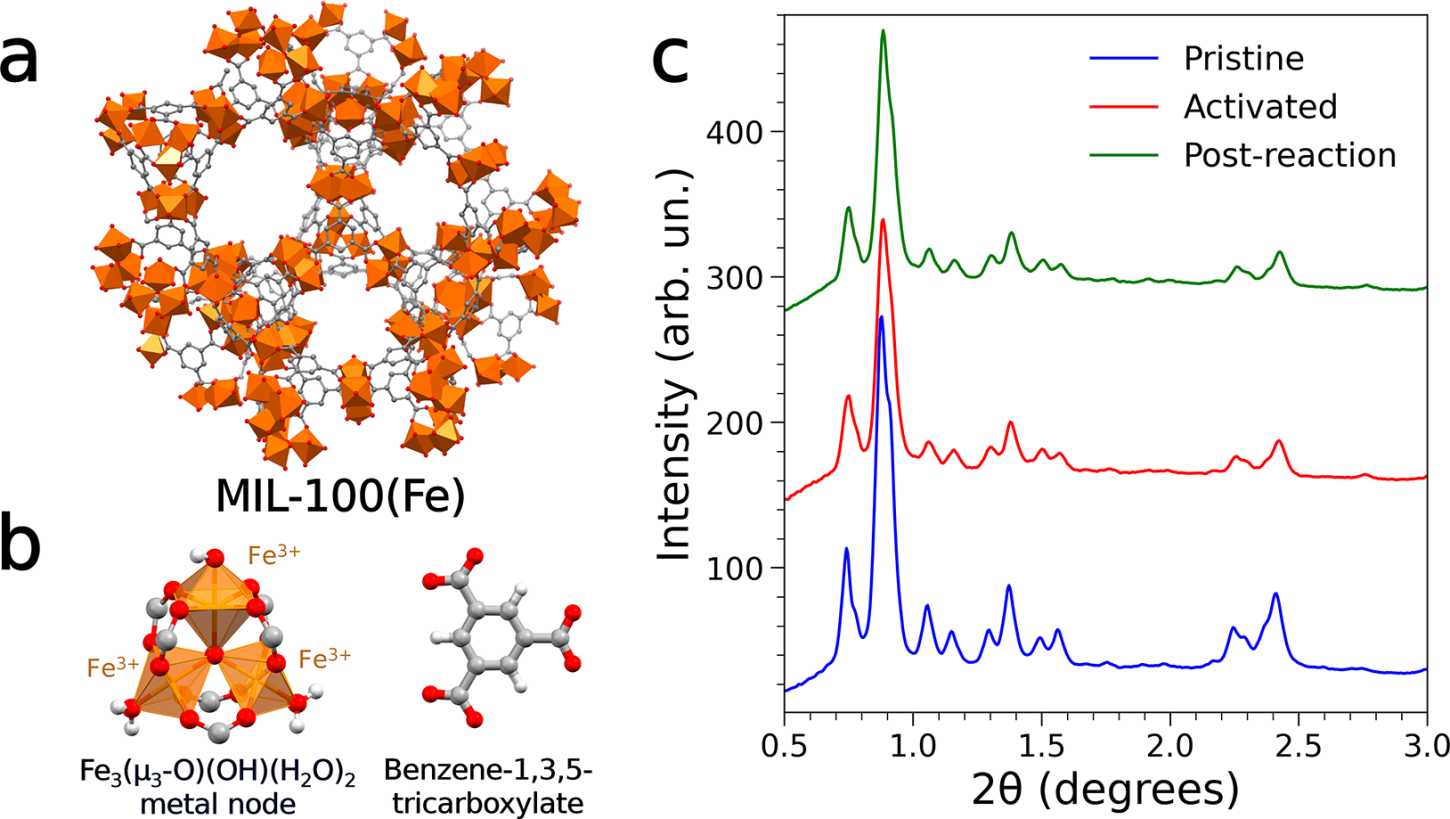
(a) Crystal structure of MIL-100(Fe). (b) Depiction of the triiron oxo-centered clusters in the MIL-100(Fe) building unit (left) and of the BTC ligand (right), highlighting the water molecules and hydroxide ion coordinating the Fe(III) centers. Color code: Fe(III), orange, O, red, C, gray, H, white. Hydrogen atoms in the structure are omitted for clarity. (c) Synchrotron PXRD patterns collected on the pristine (blue), activated at 250 °C in He (red), and postreaction MIL-100(Fe) sample (green). λ = 0.3386 Å.

In the MIL-100(Fe) crystal structure (see Figure 1a), triiron oxo-centered clusters of formula Fe_3_(μ_3_-O)(OH)(H_2_O)_2_ are linked through BTC ligands and form cages with diameters of about 25 and 29 Å, respectively.^32^ The Fe(III) ions are coordinated by the carboxylate groups in a slightly distorted octahedral geometry with an oxygen atom acting as the common vertex (μ_3_-O), while two water molecules and an hydroxide ion cap the octahedra (Figure 1b).

It is known that, upon temperature treatment above 200 °C, MIL-100(Fe) samples synthesized with HF-free procedures undergo H_2_O and HO^–^ desorption from the Fe(III) trimers, leaving behind high-spin (S = 2) Fe(II) CUSs.^33,57^ Preliminary in-house VT-PXRD experiments were therefore carried out to assess the stability of the investigated sample, and the PXRD patterns (Figure S3) did not show any loss in the long-range structural ordering of the MOF up to 310 °C. A structureless Le Bail refinement on each measured pattern (Table S1, Figure S4) confirmed that the MIL-100(Fe) phase is maintained in the essayed temperature range besides a gradual contraction of the unit cell axis (from 73.20 Å, at 30° to 72.68 Å, at 310 °C) due to the known desolvation processes.

**Figure 2:**
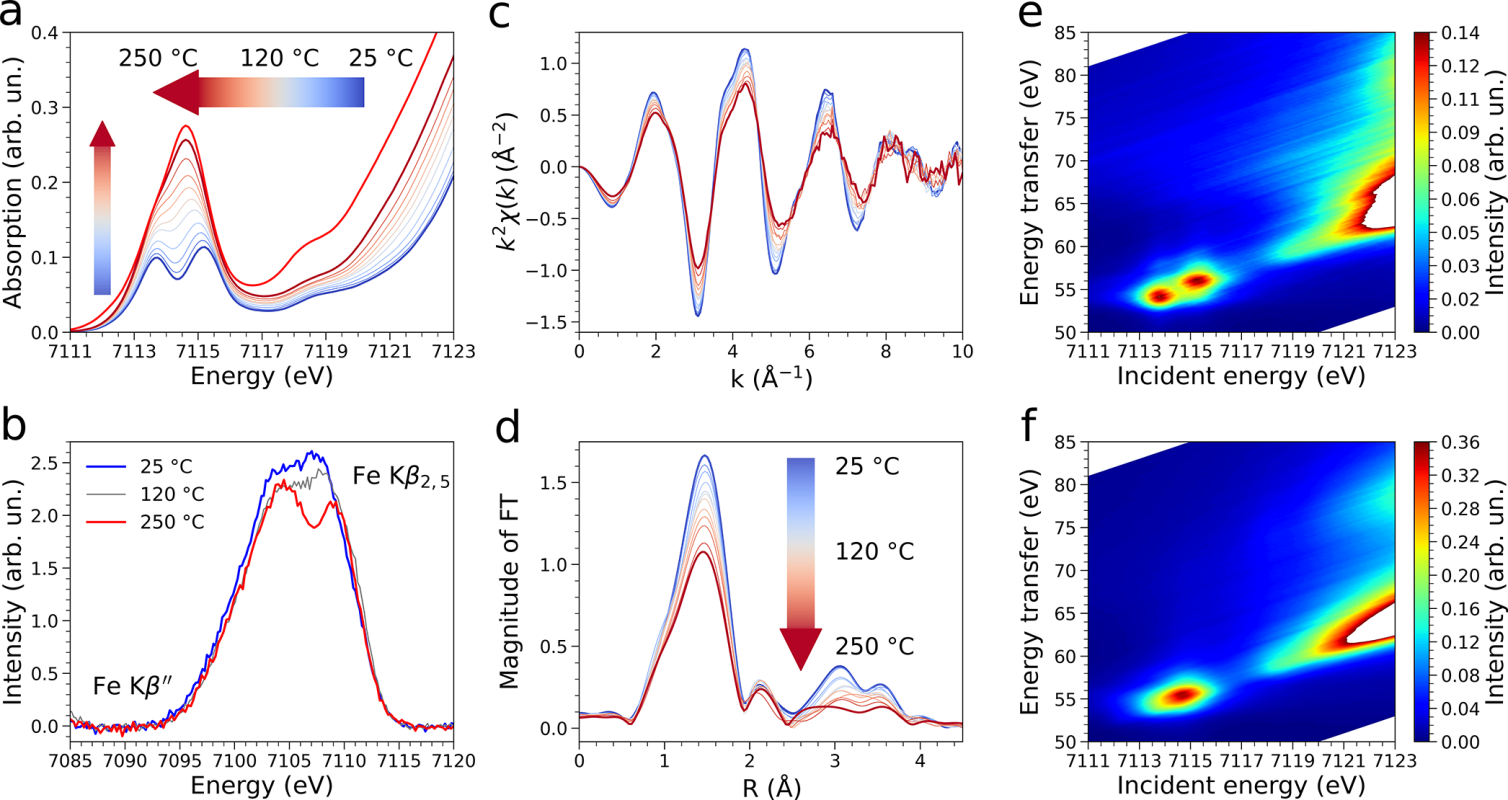
Thermal activation of the MIL-100(Fe) sample between 25 and 250 °C monitored by X-ray spectroscopic techniques. (a) Sequence of Fe K-edge HERFD-XAS spectra collected during sample activation in He flux (blue, 25 °C; dark red, 250 °C). The XAS spectrum of the sample after thermal treatment at 250 °C in He for 4 h is depicted in bright red. (b) Fe Kβ_2,5_ and Kβ″ VtC-XES spectra (collected in He flux) of the pristine sample (blue), of the sample measured at 120 °C during activation (gray), and after thermal treatment at 250 °C for 4 h (bright red). (c) Temperature evolution of the Fe K-edge EXAFS signal, *k*^2^χ(*k*), during thermal activation of the sample (blue, 25 °C; dark red, 250 °C). (d) Nonphase shift corrected FT magnitude of the EXAFS signal (same color code as panel c). (e) Fe 1s3p RIXS plane of the pristine sample. (f) Fe 1s3p RIXS plane of the sample activated at 250 °C in He.

Subsequently, we followed the thermal activation of MIL-100(Fe) by combining synchrotron HERFD-XAS, XAS-PXRD, VtC-XES, and 1s3p RIXS measurements in order to gain accurate insights into the structural and electronic modifications induced by thermal treatment on the pristine MOF. Figure 2a shows the evolution of the Fe K-edge HERFD-XAS spectra measured upon activation of the MIL-100(Fe) sample in He flux from room temperature to 250 °C and after thermal treatment at 250 °C for 4 h. In the X-ray absorption near-edge structure (XANES) spectrum of the pristine MOF, the pre-edge region presents two main features located at ∼7113.7 and 7115.1 eV, which are due to 1s → 3d transitions into the *e*_*g*_ and *t*_2*g*_ orbitals.^58^ As the temperature is increased, these two features progressively coalesce into a broad peak centered at ∼7114.6 eV that presents a shoulder at ∼7113.7 eV, as shown in the XANES spectrum measured at 250 °C (Figure 2a). In fact, during thermal treatment the loss of water and hydroxide ligands is known to provoke a change in the geometry of the iron sites from distorted octahedral to approximate square pyramidal, with contextual reduction of one of the three Fe(III) sites of the trimer to Fe(II).^33^ The XAS spectrum measured after treatment at 250 °C for 4 h also exhibits a peak at ∼7118.3 eV and a slight intensity increase around ∼7112.1 eV, both of which are due to the formation of Fe(II) sites.^58^ The observed transitions in the pre-edge region are consistent with those expected for a system where a mixture of high spin Fe(II) and Fe(III) sites is present.^58^ The edge position of the XANES spectra shifts from approximately 7124.6 to 7123.3 eV during the thermal activation process, due to the partial iron reduction. In addition, the MOF activation was followed by employing the same experimental procedure in a combined synchrotron XAS/PXRD experiment. Figure S8 presents a structureless Le Bail refinement of the PXRD pattern collected on the sample prepared for this experiment before thermal treatment. This analysis confirmed that the sample preparation (section 1.6 of the SI) did not produce alterations of the MOF structure, although a slight decrease in the long-range ordering is observed due to the pelletization process. The synchrotron PXRD pattern collected on the sample activated at 250 °C in He flow (Figure 1c) again showed that the MIL-100(Fe) crystal structure is stable up to this temperature. The evolution of the PXRD patterns recorded during the thermal treatment is shown in Figure S9, and it is in full agreement with that observed during the in-house VT-PXRD experiment. The XANES spectra collected with lower resolution as compared with the data of Figure 2a are shown in Figure S10 and are fully consistent with the observed trends in the HERFD data. The corresponding extended X-ray absorption fine structure (EXAFS) spectra extracted from the raw XAS data collected during the activation process are shown in Figure 2c, while Figure 2d presents the magnitude of the Fourier Transform (FT) calculated in the 1.8–9.0 Å^–1^*k* range. The intensity decrease of the FT first peak is due both to a decrease in the number of oxygen atoms coordinating the iron centers during activation and to the increase in the Debye–Waller factor associated with thermal and structural disorder. This finding is consistent with the HERFD-XAS results, and can be understood as caused by the release of H_2_O and HO^–^ ligands that are located at an average 2.00 ± 0.05 Å distance from the Fe(III) centers in the pristine MOF crystal structure.^32^ The VtC-XES spectra of the pristine MOF measured at room temperature, 120 °C, and 250 °C during thermal activation are shown in Figure 2b and display appreciable differences. In the Kβ_2,5_ region, the VtC-XES spectrum of the pristine MOF presents a broad peak likely split into two unresolved transitions at ∼7104.1 and 7107.3 eV, the latter being more intense. As the temperature is brought up to 120 °C, the relative intensity at 7107.3 eV decreases, and a +1.4 eV shift of the highest energy transition is observed. The VtC-XES spectrum of the activated sample displays two resolved transitions at ∼7104.5 and 7109.1 eV in the Kβ_2,5_ region. Conversely, the limited signal-to-noise ratio hampers the detection of appreciable components in the Kβ″″ region. These modifications are expected to be caused by the release of the H_2_O ligands at 120 °C and of the HO^–^ groups above 200 °C, with contextual formation of Fe(II) sites. The obtained insights into the activation process are corroborated by 1s3p RIXS measurements. In a 1s3p RIXS experiment, a 1s electron is resonantly excited into a 3d orbital. This leads to a 1s^1^3dn^*n*+1^ intermediate state that decays into a 3p^5^3dn^*n*+1^ final state, showing distinct multiplet structures for different oxidation states and thus allowing their unambiguous assignment.^59^ Full 1s3p RIXS planes of the pristine and activated (250 °C, 4 h) samples are shown in Figures 2e and f, respectively. Further, 1s3p resonant XES spectra collected at incident energies of 7113.7, 7115.1, and 7124.5 eV are depicted in Figure S11. Note that the incident energies at 7113.7 and 7115.1 eV were selected to probe the pre-edge excitations in the XANES region.^59^ One may observe that resonant excitation at the three chosen incident energies clearly distinguishes the oxidation states of the two samples. In fact, upon thermal activation, the energy splitting between the Kβ′ and Kβ_1,3_ features decreases of ∼1.1 eV, for all incident energies, while the intensity of the Kβ′ transition is reduced when exciting at 7113.7 and 7115.1 eV. The decrease in splitting between the Kβ′ and Kβ_1,3_ is expected to be due to the decrease in unpaired d electrons when Fe(III) sites are reduced to the Fe(II) sites.^59^ Altogether, the XAS and XES measurements clearly indicate that in the activated MOF a fraction of Fe(II) sites is present.

**Figure 3:**
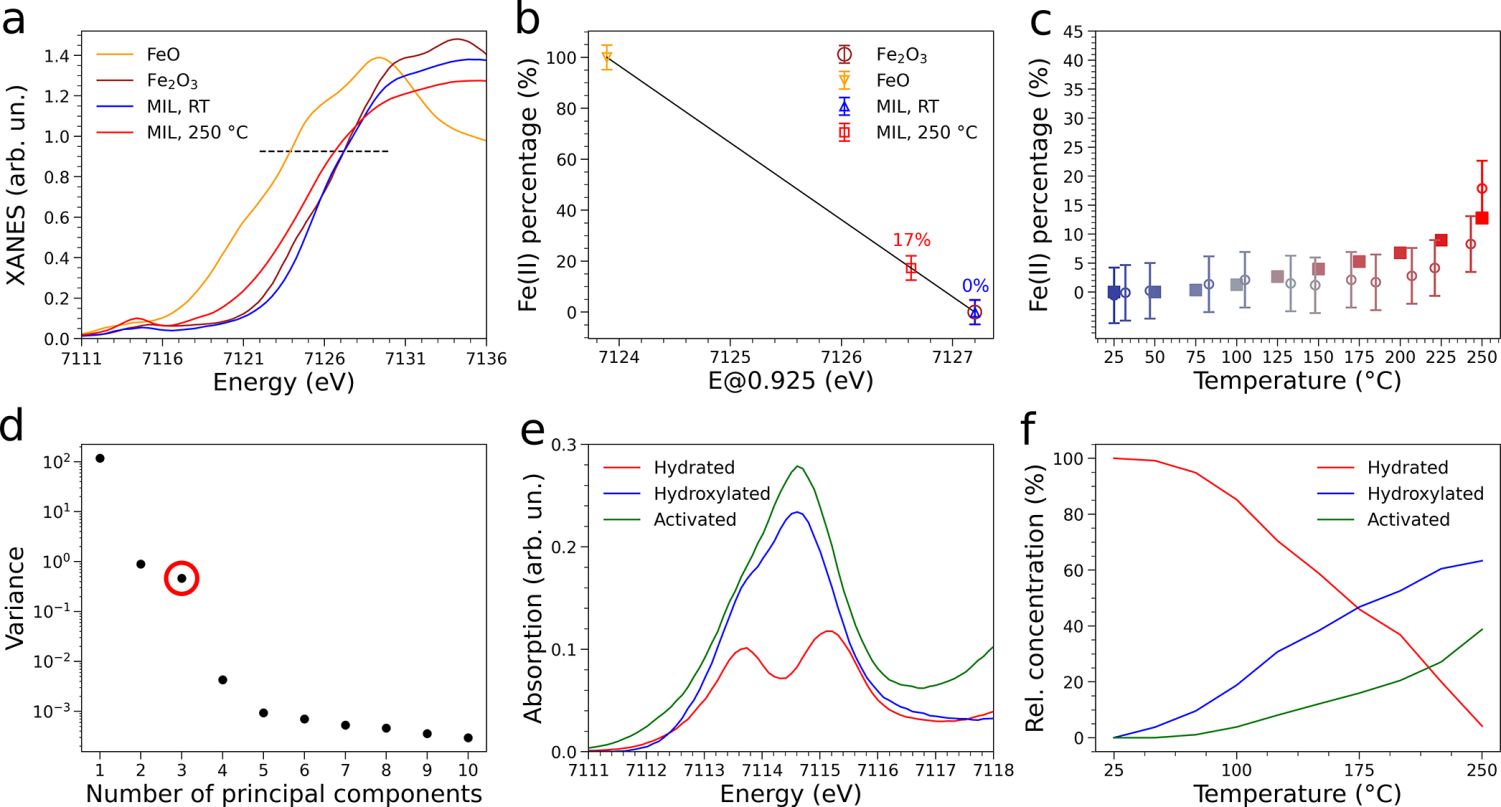
(a) Fe K-edge XANES spectra of FeO, Fe_2_O_3_, pristine MIL-100(Fe) and MIL-100(Fe) activated at 250 °C in He. The dashed line highlights the normalized intensity value at which the Fe(II) percentage has been determined. (b) Linear correlation between the energy position at 0.925 normalized intensity and the Fe(II) content in FeO (100% Fe(II)) and Fe_2_O_3_ (0% Fe(II)). The calculated Fe(II) percentages for pristine and activated MIL-100(Fe) are reported above the points. Line equation: *y* = −30.22*x* + 215396.78. (c) Comparison of the temperature evolution profile of the Fe(II) percentage calculated by linear correlation of panel b (dots with a ±5% error bar) and the relative concentration of the activated trimer component scaled by 1/3. (d) Scree plot reporting the explained variance of each PC in the HERFD-XAS data set. (e) Spectral profiles of the extracted pure components. (f) Temperature evolution of the relative concentration profiles for the three extracted components.

It is well known that, in the rising edge region, the Fe K-edge XAS spectra show transitions from core to bound states, namely 1s → 4s (edge shoulder), and 1s → 4p (edge crest),^60,61^ and these features have been found to correlate linearly with the iron oxidation state.^62^ Consequently, in order to estimate the evolution of Fe(II) content during activation, we followed the variation of the normalized intensity at a predefined height in the rising edge of the XANES spectra, as a function of temperature. We employed a previously reported procedure, using FeO and Fe_2_O_3_ as references (additional details are provided in Section S2.1 of the SI).^60,62^ This analysis indicated that while the Fe(II) content is negligible for *T* < 200 °C, it quickly rises up to about 17% at 250 °C after 1 h (see Figure 3c). Since the highest achievable Fe(II) content for a fully activated MOF is 33%, this finding suggests that after ∼1 h of thermal treatment about half of the Fe(III) trimeric units have been activated.

In order to single out the differently coordinated iron trimers in the activated sample and to obtain quantitative information on the number, nature and concentration evolution of the key species present during the thermal activation of MIL-100(Fe), a MCR-based analysis of the HERFD-XAS data was performed (see Section S2.2 of the SI), supported by Principal Component Analysis (PCA).^63−66^ Indeed, the HERFD-XAS data are expected to be very sensitive to the local MOF structural and electronic changes during activation, especially in the pre-edge region. First, the number *N* of principal components (PCs) contributing to the HERFD-XAS data mixture was determined by means of a Scree Test (see Figure 3d) and found to be *N* = 3. In fact, the presence of an elbow in the scree plot indicates that, for a number of components greater than three, the related singular values contribute to the HERFD-XAS data set reconstruction with approximately the same decaying slope and are thereby associated with noise. Next, a MCR-based decomposition was applied to the HERFD-XAS data in the pre-edge region. The extracted pre-edge regions of the spectra and concentration profiles of the three key components are shown in Figures 3e and 3f, respectively. The analysis revealed that two of the three components are assigned to the XAS spectra of the pristine and Fe(II)-containing activated samples (Figure 3e). The concentrations of the pristine and activated MOF species decrease and increase with temperature, respectively, as shown in Figure 3f. Note that the XANES spectrum of the component associated with the third additional species (Figure 3e) shows a pre-edge region with a broad peak at 7114.6 eV and a shoulder at 7113.7 eV. Notably, the intensity at 7112.1 eV, which is associated with the presence of Fe(II) centers,^58^ is negligible in the XAS spectrum of this component, indicating that it does not account for Fe(II) sites. The concentration evolution of this component is shown in Figure 3f: it gradually increases during activation and reaches a maximum value of ∼60% at 250 °C after 1 h. We note that the iron sites in both the pristine and activated trimers are antiferromagnetically coupled,^67^ and thereby the trimeric units constitute systems where the individual Fe(II) and Fe(III) contributions cannot be isolated. For this reason, the third XAS spectral component may be associated with a configuration of the MOF trimeric unit where the two H_2_O molecules have been removed, the HO^–^ group is still conserved, and all iron sites remain in the +3 oxidation state. The fact that three components account for most of the variance in the XANES data set supports the hypothesis that upon temperature treatment the H_2_O and HO^–^ groups are not released simultaneously, but rather in a sequential process.^33^

We point out that the full activation of MIL-100(Fe) requires more than 24 h,^37^ and it is reasonable that in our experimental conditions a significant fraction of trimers with intact hydroxy groups is still present after ∼1 h. Further thermal treatment for 4 h at 250 °C leads to additional activation of the MOF sample, yielding an estimated ∼22% Fe(II) content (see Figure 2a). Accordingly, it is expected that upon full sample activation, the concentration of the MOF hydroxylated fraction would decay to zero, with a corresponding increase of the Fe(II)-containing fraction. In addition, the MCR-extracted concentration profile of the activated trimeric unit is in good agreement with the previously estimated Fe(II) content evolution, within the experimental error (see Figure 3c).

**Figure 4:**
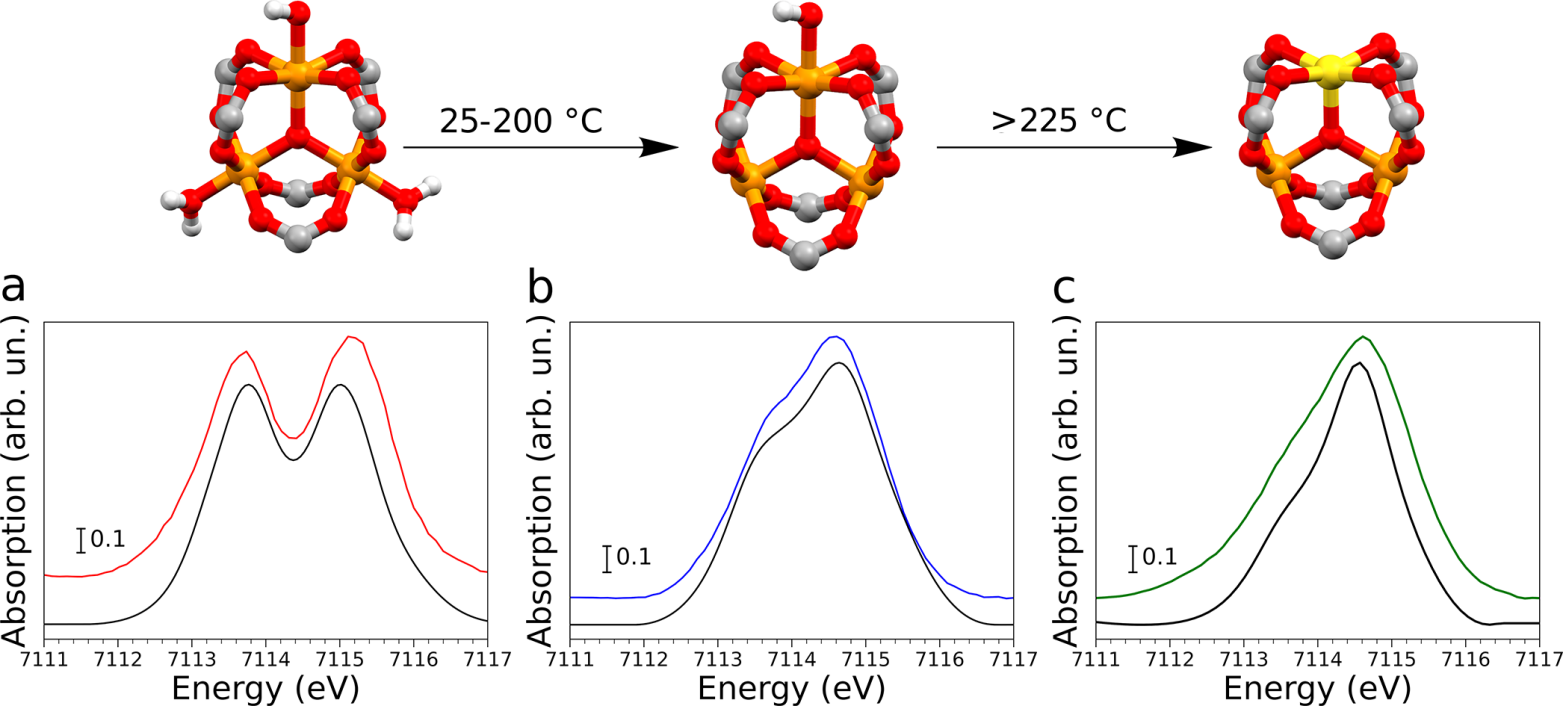
Upper panel: schematic representation of the proposed activation process of MIL-100(Fe). Color code: Fe(III), orange, Fe(II), yellow, O, red, C, gray, and H, white. The phenyl groups of the BTC ligands have been omitted for clarity. Lower panel: pre-edge region of the experimental Fe K-edge spectra of pristine MIL-100(Fe) (a, red), of the MCR-extracted component associated with hydroxylated Fe(III) sites (b, blue), and of the MOF activated at 250 °C for 4 h (c, green) compared to the corresponding theoretical curves (black).

To validate the proposed activation picture (which is summarized in the upper panel of Figure 4), an additional *ab initio* XAS analysis was carried out focusing on the pre-edge region.^47,48^ First, to benchmark the employed framework (fully discussed in Section S2.3 of the SI) the theoretical XAS spectrum of the pristine MOF was calculated starting from the available crystal structure.^32^Figure 4a compares the pre-edge features of the theoretical signal with the experimental XAS spectrum of the pristine MOF. One may note that the calculated energy positions and relative intensities of the transitions are in excellent agreement with the experimental ones. Having established the reliability of our method, we calculated the theoretical XAS signal for a trimeric cluster of MIL-100(Fe), where the H_2_O molecules have been removed and one HO^–^ ion coordinates a Fe(III) site. The pre-edge region of the theoretical spectrum is compared to the XAS spectrum of the MCR-extracted component that is associated with the hydroxylated trimers in Figure 4b. Also in this case the agreement between the theoretical and experimental curves is excellent, thereby confirming the proposed chemical identification of this component. Finally, the theoretical XAS spectrum of a fully activated trimeric unit is compared in Figure S14 to the HERFD-XAS experimental spectrum measured after treating the MOF for 4 h at 250 °C. One may observe that while the main experimental transition at 7114.6 eV is properly reproduced by the theoretical calculation, the less intense shoulder at 7113.7 eV is not accounted for. This lack of agreement may be attributed to an incomplete activation of the MOF sample. In fact, by combining the theoretical XAS spectra of the activated (Figure S14) and hydroxylated (Figure 4b) trimers in a 2:1 ratio it is possible to reproduce the experimental spectrum (Figure 4c), indicating that ∼66% of available MOF units contain Fe(II). Altogether, the presented spectroscopic evidence strongly suggests that a vast majority of the triiron sites present an intact, defect-free structure. Although the presence of structural defects over the metal nodes (e.g., missing-linker sites) cannot be completely excluded, it appears reasonable to conclude that, if such defective sites are present in the analyzed MIL-100(Fe) sample, their relative abundance is very limited.

### MTM Reaction with Molecular Oxygen

After activating the MIL-100(Fe) sample, we exposed the MOF to a mixture of CH_4_ (3 mL/min) and O_2_ (3 mL/min) in a preliminary experiment performed at 200 °C, using a 2 mm-diameter capillary reactor with increased sample mass. Remarkably, in these conditions, methanol production is observed, as shown by the evolution of the *m*/*z* = 31 mass profile presented in Figure S15a. Methanol is produced mainly within the first few minutes of the reaction, together with its overoxidation products (mainly carbon dioxide), which constitute the prevalent species formed during the MTM process (see Figure S16). The formation of dimethyl ether, a valuable oxygenate arising from the condensation of two methanol molecules, is also observed.

**Figure 5:**
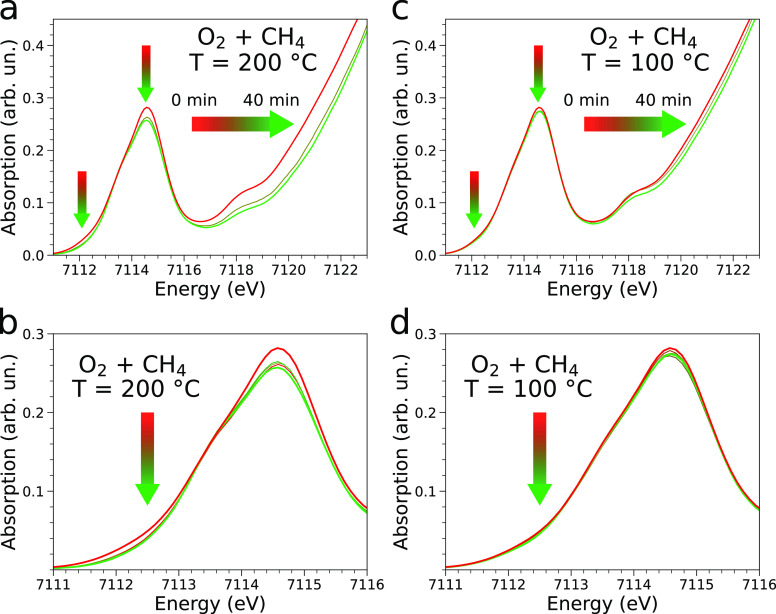
(a) Fe K-edge HERFD-XAS spectra collected on activated MIL-100(Fe) (red), while performing the MTM reaction over the activated sample with a mixture of O_2_ and CH_4_ at 200 °C (20 min, olive) and after the reaction (40 min, green) and (b) magnification of the 7111–7116 eV region. (c) Fe K-edge HERFD-XAS spectra collected on activated MIL-100(Fe) (red), while performing the MTM reaction over the activated sample with a mixture of O_2_ and CH_4_ at 100 °C (20 min, olive) and after the reaction (40 min, green) and (d) magnification of the 7111–7116 eV region. The arrows highlight the most significant spectral features.

Accurate insights into the evolution of the structural and electronic properties of the MOF catalyst during the MTM reaction can be obtained by combining X-ray spectroscopy and diffraction techniques. We used HERFD-XAS to monitor the oxidation state of the iron active sites during the O_2_-based MTM conversion over MIL-100(Fe) at 200 °C. As shown in Figures 5a,b the HERFD-XAS spectrum of the activated sample (red curve) differs significantly from that of the postreaction MOF (green curve) recorded at *t* = 40 min. The following spectral variations are observed: (i) the intensity of the Fe(II)-related feature at 7112.1 eV is depleted, (ii) the intensities of the transitions at 7114.6 and 7118.3 eV decrease, and (iii) the edge undergoes a ∼0.4 eV shift toward higher energies. Further, in the 1s3p Kβ resonant XES spectra collected at incident energies of 7113.7 and 7115.1 eV on the postreaction MOF there is an increase in the energy splitting between the Kβ′ and Kβ_1,3_ features if compared to the same resonant XES spectra measured on the activated material (see Figure S17). This indicates that the Fe(II) sites were oxidized to Fe(III). All this evidence suggests that the Fe(II) content initially active in the MOF catalyst is consumed during the reactive MTM process. The same trends are observed in the analogous lower resolution XAS-PXRD experiment (see Figure S18). Also in this case, we detected the formation of methanol under MTM reaction conditions (see Figure S15b). As for the EXAFS data, a small increase in the FT magnitude of the first peak is observed on the postreaction MOF. Note that although slight, this variation in the FT magnitude suggests that in the postreaction catalyst a fraction of the open iron sites have been coordinated by ligands that can hardly be identified by EXAFS alone.

After having assessed the ability of MIL-100(Fe) to catalyze the MTM reaction using O_2_ as the oxidant, we investigated the propensity of the material toward active site regeneration, which is a key issue in catalysis. To this end, we performed additional XAS-PXRD experiments. The postreaction MOF was reactivated by thermal treatment in He gas flow at 250 °C (for 1 h) and subjected to a second reaction cycle at 200 °C. Also in this case, we observed methanol production (see Figure S19). Figure S20 presents XAS monitoring of the second reaction cycle. Altogether, during this additional reaction cycle the XAS data exhibit changes in the pre-edge region very similar to those observed during the first reaction cycle, with a positive edge shift of ca. 0.4 eV in the XAS spectra. Figure S21 shows that the XAS spectra of the activated sample before (first activation) and after the first (second activation) and second (third activation) reaction cycles are nearly superimposable. Moreover, the PXRD patterns remained unchanged during the whole experiment (Figures S22–S24). The evolution of the MOF active Fe(II) content during the first and second MTM reaction cycles was estimated following the normalized intensity in the rising edge region of the XAS spectra, as previously discussed. As shown in Figure S25a,b, the initial Fe(II) fraction (17% after 1 h of thermal treatment) is depleted during the first reaction cycle, while ∼16% is recovered after reactivating the sample. The second reaction cycle again consumes the available Fe(II) sites. Importantly, performing the MTM reaction using O_2_ over MIL-100(Fe) at 100 °C also led to methanol production as evidenced by the evolution of the *m*/*z* = 31 MS signal (see Figure S15b). At 100 °C we observe changes in the HERFD-XAS (Figure 5c,d) and EXAFS (Figure S26) spectra that are very similar to those recorded when performing the MTM reaction at 200 °C. The same holds for the PXRD measurements shown in Figure S27. As expected, at 100 °C the intensity decay of the feature at 7112.1 eV in the HERFD-XAS spectra is very slight, and the shift of the absorption edge to higher energies is slower than at 200 °C, likely due to a reduced reaction kinetics. Conversely, we did not measure methanol production within our experimental sensitivity when exposing activated MIL-100(Fe) to a mixture of O_2_ and CH_4_ at room temperature, although performing such experiment resulted in modifications of the HERFD-XANES spectra that are fully in line with those observed at 200 and 100 °C (Figure S28). In order to obtain further insights into the nature of the observed O_2_-based MTM reactivity, we performed the MTM reaction at 200 °C changing the oxidant to N_2_O. As previously discussed, the N_2_O-based hydroxylation of methane over MIL-100(Fe) has been well documented.^16,37^ The corresponding time evolution of the Fe K-edge HERFD-XAS spectra is shown in Figure S29, and one may see that the spectroscopic changes are almost identical with those observed when the reaction is performed with O_2_. The same holds true for the resonant XES spectra collected under the same conditions (Figure S30). Subsequent investigations will be devoted to understanding whether the MIL-100(Fe) system may catalyze the MTM conversion at room temperature with O_2_ acting as the oxidant as well as to quantitatively screen productivity and selectivity as a function of the reaction temperature. The reaction was carried out cofeeding both reactants as a mixture, rather than following a stepwise oxidation-steam desorption protocol, in order to explore reaction conditions more suitable for streamlined industrial processes.

### Reaction Mechanism

The use of O_2_ as an oxidant in the MTM conversion requires dissociation of the strong O–O bond. In Fe-based catalysts, two possible O_2_ activation pathways have been proposed. The first mechanism requires two adjacent Fe(II) sites that cooperatively dissociate O_2_, a process that has been suggested to occur, among others, in sMMO and in the Ferrierite zeolite.^6,22^ Conversely, in the second mechanism, O_2_ adsorbs with an “end-on” configuration on an isolated Fe(II) site, leading to the formation of an Fe(IV)=O center through a putative ferric–hydroperoxo intermediate. This pathway has been proposed for a number of enzymes able to cleave the O–O bond.^38−40,68,69^ We note that in MIL-100(Fe) the distances between iron sites within individual and far apart trimeric units are equal to ∼3.4 and 7.3 Å, respectively. Further, the electronic and structural configurations of the trimers do not easily allow the interaction of O_2_ with more than one iron site. For these reasons, one may exclude the cooperation of more than one Fe center in the dissociation of O_2_ for the MTM process over this MOF.

In order to gain experimental insights into the mechanism of the MTM conversion over MIL-100(Fe), we investigated the interaction between the MOF and O_2_. Figure S31a compares the XAS spectrum recorded on a MOF sample pretreated at 250 °C for 1 h (black curve) to the XAS spectrum measured after MOF exposure to O_2_ for 30 min at 200 °C. A positive shift (∼0.5 eV) in the edge energy is observed, indicating that the Fe(II) active sites interacted with adsorbed O_2_. The slight increase of the FT transform magnitude at 1.5 Å (Figure S31b) can be associated with an increase of the iron coordination number, supporting this picture. Notably, as shown in Figure S32, the XAS spectrum measured after exposing the MOF to O_2_ and the one collected on the postreaction MOF are nearly superimposable, suggesting that in both cases the same prevalent species is formed. The species that arises upon the O_2_ interaction also persists once the MOF sample is exposed to a CH_4_ flux and to a O_2_ + CH_4_ gas mixture at 200 °C. In fact, as shown in Figures S31c and S31d, the XANES and EXAFS spectra recorded during exposure to CH_4_ (green curve) and the O_2_ + CH_4_ (purple curve) fluxes almost coincide within the experimental error.

Once the temperature is brought back to 250 °C, the recorded XAS spectrum (Figure S31e, red curve) and its FT magnitude (Figure S31f) are nearly identical with those of the previously activated material. These findings indicate that the initial Fe(II) fraction has been recovered and that the interaction established between O_2_ and the MOF is fully reversible upon heating at 250 °C. In addition, the PXRD patterns recorded while exposing the MOF to O_2_, CH_4_, and O_2_ + CH_4_ gas fluxes show no appreciable variation (see Figure S33) confirming that the sample has retained its long-range order and crystal structure. Finally, as shown in Figure S34, there is an appreciable increase in the signals at *m*/*z* = 17 and 18 during the O_2_ flux, indicating that water and HO^–^ ions are formed. A very similar effect has been observed in the FeDOBDC (DOBDC^4–^ = 4,6-dioxido-1,3-benzenedicarboxylate) system, a MOF presenting open Fe(II) sites in its structure. Upon exposure of this MOF to pure N_2_O, IR measurements showed the formation of hydroxylated Fe(III) groups in significant quantities.^70^

**Figure 6:**
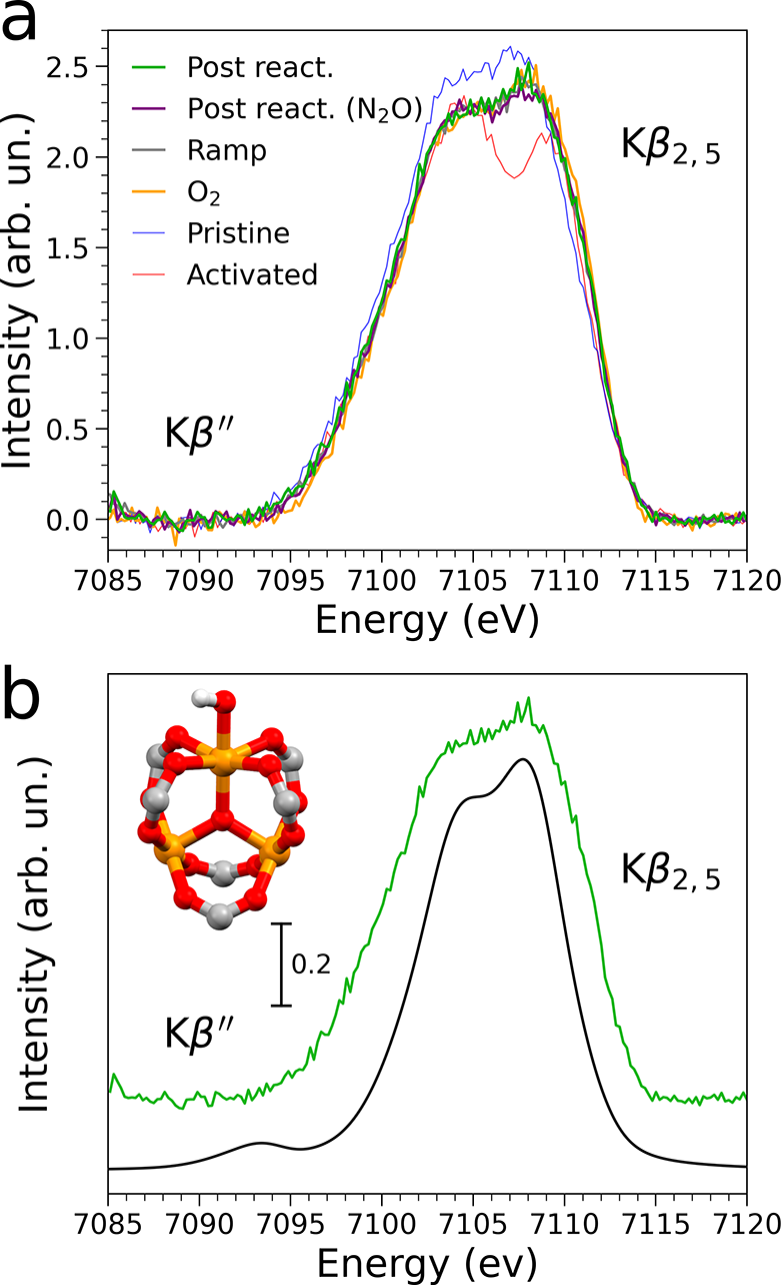
(a) Fe Kβ_2,5_ and Kβ″ VtC-XES spectra collected on the pristine MIL-100 (Fe) material in He flux at 25 °C (blue), at 250 °C in He (red), at 120 °C in He during thermal treatment (gray), at 200 °C in O_2_ flux (yellow), at 200 °C in He on the postreaction sample (green) and at 200 °C in He on the sample reacted with CH_4_ and N_2_O. (b) Comparison between the experimental Fe VtC-XES spectrum collected on the postreaction MOF at 200 °C (green) and the theoretical VtC-XES spectrum of a hydroxylated trimeric unit (black). The hydroxylated cluster is also shown in the panel (color code: Fe(III), orange, O, red, C, gray, and H, white).

It is important to observe that the information provided by XAS and PXRD results alone does not allow one to unambiguously determine the identity of the prevalent species formed when MIL-100(Fe) interacts with O_2_ and CH_4_. For this reason we employed VtC-XES, a technique renowned for its sensitivity to subtle ligand modifications,^31,59,71^ to gain experimental insights into the mechanism of MTM conversion over MIL-100(Fe). Figure 6a compares the VtC-XES spectra measured on the MIL-100(Fe) sample during its exposure to O_2_ at 200 °C (yellow curve), after the MTM reaction was performed at 200 °C with O_2_ (green curve) and N_2_O (purple curve) and at 120 °C during the initial thermal activation. Importantly, the four VtC-XES spectra are nearly superimposable. Only very small intensity differences at ∼7103.0 and ∼7110.0 eV may be observed in the Kβ_2,5_ region of the spectra. This evidence strongly suggests that the hydroxylated trimer is the prevalent trimeric unit that arises when the activated MOF is exposed to an O_2_ and CH_4_ mixture as well as pure O_2_. In fact, at 120 °C approximately 40% of the MOF trimers (see Figure 3e) are hydroxylated, while the Fe(II) content is still very low. Moreover, the slightly higher intensity at ∼7103.0 eV in the VtC-XES spectrum measured at 120 °C during thermal treatment could be attributed to the presence of the residual fraction of hydrated trimeric units. The VtC-XES technique is in fact sensitive to the amount of desorbed water molecules as evidenced by the spectral differences between the VtC-XES spectra of the pristine and partially dehydrated MOF samples (blue and gray curves, respectively, in Figure 6a). We point out that the fact that hydroxylated trimers are abundant in the postreaction MOF is consistent with previous work, where IR measurements have shown that a significant amount of hydroxylated Fe–OH sites form when performing the N_2_O-based MTM conversion over PCN-250(Fe).^16^ Since PCN-250(Fe) and MIL-100(Fe) are constituted by iron trimeric units with analogous electronic and structural properties, it is reasonable to expect a similar behavior under MTM reaction conditions.

Figure 6b presents the theoretical VtC-XES spectrum calculated for an hydroxylated Fe(III) cluster which shows an excellent agreement with the experimental spectrum measured on the postreaction MOF. This combined evidence strongly suggests that the prevalent trimeric unit that arises when exposing the activated MOF either to O_2_ or O_2_ + CH_4_ is the hydroxylated trimer. In fact, at 120 °C about 40% of the MOF trimers (see Figure 3e) are hydroxylated, while the Fe(II) content is still very low. Moreover, the slightly higher intensity at ∼7103.0 eV in the VtC-XES spectrum measured at 120 °C during thermal treatment could be attributed to the presence of the residual fraction of hydrated trimeric units. The formation of H_2_O and HO^–^ when exposing the MOF to O_2_ (Figure S34) reinforces this hypothesis. The fact that hydroxylated trimers are abundant in the postreaction MOF is consistent with previous work, where IR measurements have shown that a significant amount of hydroxylated Fe–OH sites form when performing the N_2_O-based MTM conversion over PCN-250(Fe).^16^ Since PCN-250(Fe) and MIL-100(Fe) are constituted by iron trimeric units with analogous electronic and structural properties, it is reasonable to expect a similar reaction behavior.

**Figure 7:**
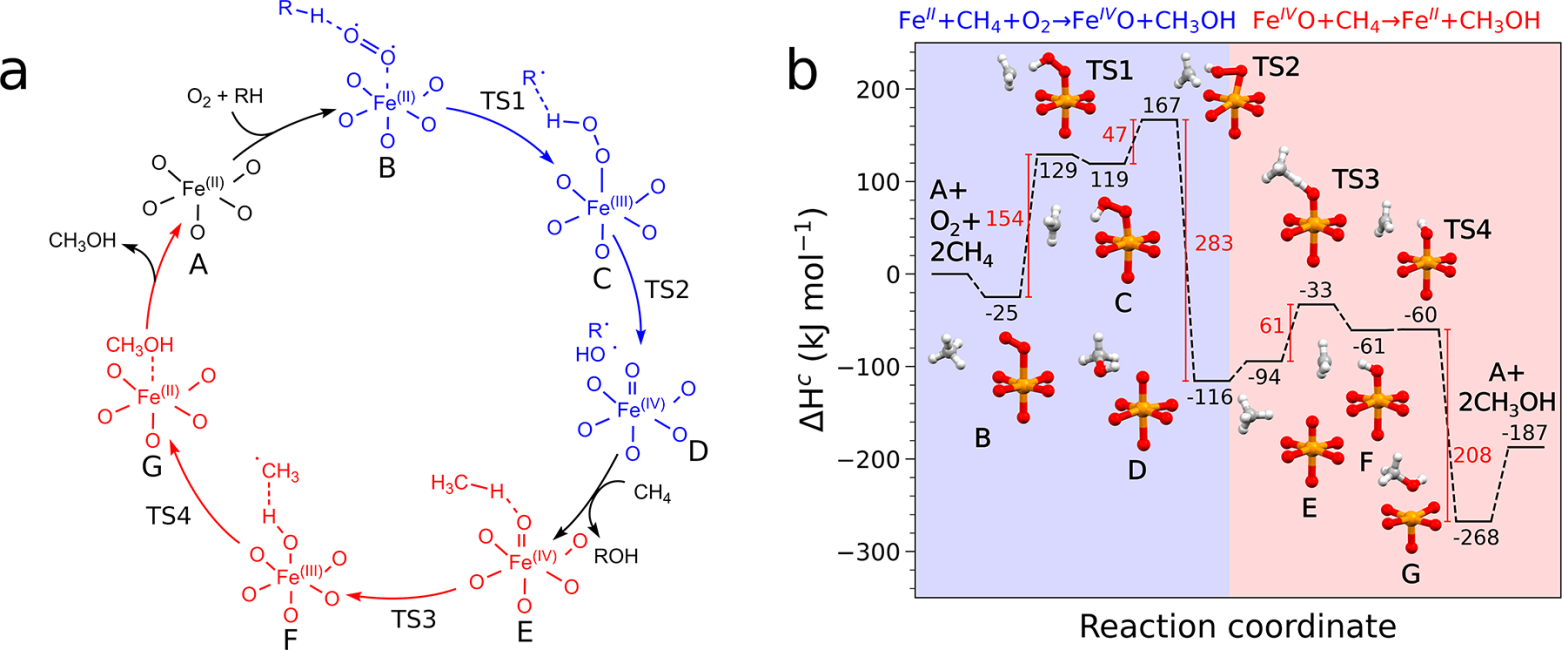
(a) Proposed mechanistic scheme of the O_2_-based MTM reaction occurring over Fe(II) sites in MIL-100(Fe). The two reaction steps are highlighted in blue and red, respectively. (b) Lowest-enthalpy reaction pathway of the mechanism described in panel (a) calculated at the DFT level of theory assuming **RH** = CH_4_. The separated reactants in their ground state set the zero of the enthalpy scale. The optimized structures are depicted near the associated enthalpy values (color code: orange, iron, red, oxygen, gray, carbon, and white, hydrogen).

The presented experimental insights were used as a basis to hypothesize a catalytic cycle, supported by DFT calculations, for the MTM reaction occurring over an Fe(II) site in MIL-100(Fe) with O_2_ as an oxidant. Specifically, with our DFT modeling we aim to propose a scheme that is in reasonable agreement with our experimental evidence and the current literature regarding molecular oxygen activation over isolated Fe(II) sites.^40,68,69^ We also stress that deviations from the proposed catalytic pathway cannot be excluded and that future investigations will be needed to complete the description of the O_2_-based MTM reactivity over this system. The proposed catalytic cycle is shown in Figure 7a. In the initial step, a hydrogen atom is transferred from hydrogen donor species **RH** to an iron trimer interacting with O_2_, producing an Fe(IV)=O site through an Fe(III)-OOH intermediate. This process is ubiquitous in iron nonheme enzymatic systems, where isolated iron centers interact with O_2_.^38−40^ In addition, the previously discussed hydroxylation of iron centers upon interaction with O_2_ indicates that hydrogen atom transfer processes are in place. An hydroperoxo Fe(III)-OOH intermediate is therefore compatible with this scenario. We must remark that our experimental data do not allow us to unambiguously determine the identity of the hydrogen donor species **RH**. A number of species could act as the hydrogen donor: residual water in the MOF pores, possibly methane itself or even the framework BTC linker. It cannot be excluded that all pathways may be operating under MTM reaction conditions and that the ensuing Fe(IV)=O sites could contribute to the MTM conversion following steps **E** → **A** in Figure 7a. Future studies will therefore be dedicated to further investigating the oxygen activation process and understanding which species are involved in the hydrogen atom abstraction.

In the DFT calculations, a single Fe-trimer node was employed as a cluster model (Figure S35). In agreement with previous work by Gagliardi et al.,^72,73^ we modeled the reaction assuming a high spin state (2S+1 = 15) for the trinuclear unit neglecting antiferromagnetic stabilization effects. This choice is motivated by the fact that broken symmetry wave functions, which are able to correctly describe the spin coupling effects present in the MIL-100(Fe) trimers, are not spin eigenfunctions and they do not correctly reproduce the spin density of the system.^72,73^ In addition, two distinct reaction pathways were considered, with total 2S+1 = 17 and 2S+1 = 15 multiplicities in the presence of triplet and singlet O_2_, respectively, to account for the spin transition occurring during the molecular oxygen-based MTM reaction (see Section S3 of the SI for additional details). The DFT enthalpy diagram of the proposed reactive pathway, calculated assuming **RH** = CH_4_ for modeling purposes, is shown in Figure 7b. The reaction initially proceeds on the 2S+1 = 17 spin surface, which is energetically favored (see Figures S37–S38). The first step is the “end-on” adsorption of O_2_ on an open Fe(II) site (**B**, 25 kJ mol^–1^) in the presence of CH_4_, which in our model acts as a hydrogen atom donor. After a hydrogen atom abstraction process, through transition state **TS1** (154 kJ mol^–1^, Figure 7b) intermediate **C** is reached (10 kJ mol^–1^), where a CH_3_^•^ radical interacts with a hydroperoxo group bound to the iron center. We point out that cluster models have been shown to overestimate reaction energetics for the MTM conversion over Fe(IV)=O centers in zeolites by more than 50%.^74^ Nevertheless, accurately modeling the MIL-100(Fe) giant structure requires a very large computational effort that is not justified by the related increase in thermochemical accuracy,^75^ and we expect the actual transition state energies to be lower than those predicted by our cluster DFT calculations. In a subsequent step, the FeO–OH bond is cleaved through transition state **TS2** (47 kJ mol^–1^) and the CH_3_^•^ and HO^•^ radicals recombine. This process yields the first molecule of methanol as a product and a high spin Fe(IV)=O site (intermediate **D**) releasing 283 kJ mol^–1^. As one may see in Figure S39, when the reaction proceeds from **TS2** to **D** the 2S+1 = 15 spin surface becomes the energetically favored one. The precise localization of the minimum energy crossing point (MECP) between the 2S+1 = 17 and 2S+1 = 15 spin surfaces is not straightforward. However, the presence of two mutually quasi isoenergetic and isostructural structures (see Figure S39) along the nudged elastic band pathways from **C** to **D** in both spin surfaces suggests that the MECP occurs during the FeO–OH bond cleavage. At this stage, the formed Fe(IV)=O site may interact with a second CH_4_ molecule (intermediate **D**, 22 kJ mol^–1^) to yield a second methanol molecule following a radical rebound mechanism that has been well documented to occur in the N_2_O-based MTM conversion over MIL-100(Fe).^16,34−36^ A hydrogen atom is transferred to the Fe(IV)=O center, leading to the formation of an Fe(III)–OH site and a CH_3_^•^ radical (intermediate **F**) through transition state **TS3** (61 kJ mol^–1^). Finally, a virtually barrierless step (**TS4**, 1 kJ mol^–1^) leads to the formation of the second methanol molecule (**G**, −208 kJ mol^–1^) and restores the Fe(II) open metal site. The corresponding Gibbs free energy diagram shows identical trends (Figure S40). We point out that one cannot exclude that the BTC ligands and/or residual water molecules present in the framework pores may also act as hydrogen atom donors in the first part of the MTM process and that these pathways may be operating in the presence of CH_4_ as well. Further, the deactivation of the MOF is expected to occur due to the escape of the CH_3_^•^ radical from intermediates **C** and **F**, whose desorption enthalpies are equal to ca. −20 and −10 kJ mol^–1^, respectively, in line with previous studies.^34^ This process has long been known to occur within radical rebound mechanisms involving light alkanes,^76^ where a certain degree of competition between the radical rebound and escape processes is in place.^77^ Recently, it has also been demonstrated that radical escape occurs in materials exhibiting pore apertures larger than the van der Waals diameter of CH_4_ (∼4.2 Å),^78^ whose pore windows are 5.5 Å, and 8.6 Å wide, respectively. An additional catalyst deactivation pathway could be caused by the interaction of molecular oxygen alone with an Fe(II) site in the presence of local inhomogeneities of the gas phase. As mentioned above, this interaction leads to the formation of hydroxylated iron trimers. This hypothesis is also strongly supported by the fact that dimethyl ether is produced in the reaction in significant quantities. Notably, dimethyl ether can be formed via methanol condensation or radical coupling. The former process is considered less likely to occur, but the latter has been observed, for instance, when performing the MTM reaction over the FeZSM-5 zeolite and is compatible with our experimental conditions.^79^ One cannot exclude that the interaction of molecular oxygen alone with the MOF Fe(II) sites may constitute an additional deactivation pathway. As mentioned above, this interaction leads to the formation of hydroxylated iron trimers.

Previous investigations on the MTM N_2_O-based reaction over MOFs composed of triiron nodes reported that methanol is produced after flushing the MOF with H_2_O.^16,36^ Water was proposed to interact with -OCH_3_ groups that coordinate iron sites and are formed during the reaction, yielding methanol.^16^ In our experimental conditions, as previously discussed, hydroxylated trimers are expected to constitute the larger fraction of trimeric units in the postreaction MOF. We cannot exclude, however, the presence of a lesser amount of triiron nodes where -OCH_3_ and/or other byproducts coordinate the iron sites. To investigate this possibility, we calculated a theoretical VtC-XES spectrum of a trimeric unit coordinated by a single -OCH_3_ group (see Figure S41). One may observe that the theoretical Kβ_2,5_ transition is shifted to higher energies compared to that of the hydroxylated trimer. This finding supports the hypothesis that the slight difference in intensity at ∼7110 eV between the VtC-XES spectra collected at 120 °C during activation and on the postreaction MOF could be attributed to a lower fraction of -OCH_3_ groups. The contribution from other reaction byproducts adsorbed on the MOF iron sites cannot be excluded but is reasonably very low. To further investigate this hypothesis, we exposed the postreaction MIL-100(Fe) to a He flux carrying water at 200 °C, after which we did not observe methanol production within the sensitivity of our experimental setup. This does not however exclude the presence of -OCH_3_ species forming in reduced quantities during the MTM reaction.

## Conclusions

In this work, it is shown that the direct MTM conversion using O_2_ as an oxidant occurs over MIL-100(Fe) under mild conditions. The MIL-100(Fe) activity may be regenerated by thermal treatment, as demonstrated by performing the MTM reaction for two consecutive catalytic cycles. The bulk structural properties of the MOF are retained during the reaction cycles, as evidenced by PXRD measurements. HERFD-XAS, EXAFS, XES, and RIXS measurements are employed to track the structural and electronic properties of the MOF iron sites from activation to reaction and postreaction conditions. DFT calculations guided by the experimental insights support the hypothesis that the active square-pyramidal Fe(II) centers located in the nodes of MIL-100(Fe) dissociate O_2_ leading to methanol production. As proven by VtC-XES measurements, catalyst deactivation predominantly leads to the formation of hydroxylated Fe(III) sites. This experimental evidence, supported by DFT and by the observed formation of dimethyl ether as a reaction product, strongly suggests that catalyst deactivation during the MTM reaction is, at least in part, due to the escape of CH_3_^•^ radicals from the active trimeric units. This process may be attributed to the fact that the pore apertures of MIL-100(Fe) are significantly larger than the van der Waals radius of methane.^78^ Further, one cannot exclude the presence of an additional deactivation pathway due to the interaction between the MOF Fe(II) open sites and molecular oxygen, which ultimately leads to the hydroxylation of the metal trimeric units. Future efforts to improve MTM catalytic activity and selectivity will require the design of MIL-100(Fe) analogues with reduced pore apertures. Altogether, it is shown that the combination of complementary X-ray experimental methods, supported by theory, enables a detailed comprehension of the MTM catalytic processes, providing information that would not be accessible to any of the techniques alone. We believe that our findings can lay the groundwork for further studies addressing the MTM reactivity of MIL-100(Fe) and similar MOFs with O_2_, and provide a protocol of general interest that may be extended to investigate MOFs and other materials relevant for heterogeneous catalysis.

## Supporting Information Available

Detailed information on material synthesis, characterization, X-ray absorption, emission and diffraction experiments, data analysis and DFT calculations (PDF)
XYZ coordinates of the computational models (ZIP)
