# Supplementary material for: Full Spectroscopic Characterization of the Molecular Oxygen-Based Methane to Methanol Conversion over Open Fe(II) Sites in a Metal–Organic Framework
Source: J Am Chem Soc. 2023 Sep 18;145(38):21040–52. doi: 10.1021/jacs.3c07216 (PMC10540213; doi:10.1021/jacs.3c07216)
Supplement: Supplementary file 1 — ja3c07216_si_001.pdf [file ja3c07216_si_001.pdf]

Supporting Information: Full Spectroscopic Characterization of the Molecular Oxygen-Based Methane to Methanol Conversion over Open Fe(II) Sites in a Metal-Organic Framework

Alessandro Tofoni,[†,@] Francesco Tavani,[†,@] Marco Vandone,[‡,@] Luca Braglia,[¶] Elisa Borfecchia,[§] Paolo Ghigna,[‖] Dragos Costantin Stoian,[⊥] Toni Grell,[‡] Sara Stolfi,[¶] Valentina Colombo,[*,‡] and Paola D'Angelo[*,†]

†*Dipartimento di Chimica, Università degli Studi di Roma "La Sapienza", P.le A. Moro 5, I-00185 Rome, Italy*

‡*Dipartimento di Chimica & UdR INSTM di Milano, Università degli Studi di Milano, Via Golgi 19, 20133 Milan, Italy*

¶*CNR-Istituto Officina dei Materiali, TASC, 34149 Trieste, Italy*

§*Dipartimento di Chimica & UdR INSTM di Torino, Università di Torino, Via P. Giuria 7, 10125 Turin, Italy*

‖*Dipartimento di Chimica, Università di Pavia, V.le Taramelli 13, I27100 Pavia, Italy*

⊥*The Swiss-Norwegian Beamlines (SNBL), European Synchrotron Radiation Facility, BP 220, 38043 Grenoble, France*

#*CNR - SCITEC - Istituto di Scienze e Tecnologie Chimiche "Giulio Natta", Via Golgi 19, 20133 Milan, Italy*

@*Equal contribution*

E-mail: p.dangelo@uniroma1.it; valentina.colombo@unimi.it

Contents

1 Experimental Methods **S-2**

1.1 Synthesis of MIL-100(Fe) S-2

1.2 Preliminary PXRD Characterization S-4

1.3 Infrared Spectroscopy Measurements S-6

1.4 Thermogravimetric Analysis S-7

1.5 N_2 Adsorption Measurements S-7

1.6 Operando HERFD-XAS, XAS-XRD, XES and RIXS Experiments S-7

2 Data Analysis **S-10**

2.1 Determination of the Fe(II) Content S-10

2.2 Decomposition of the HERFD-XAS Dataset into the Spectra and Fractional Concentration of the Key Components S-10

2.3 XAS and VtC-XES Calculations S-11

3 Density Functional Theory Calculations **S-11**

4 Supplementary Figures (Figures S1 - S41) **S-14**

5 Supplementary Tables (Tables S1 - S7) **S-41**

References **S-45**

1 Experimental Methods

1.1 Synthesis of MIL-100(Fe)

General literature-reported synthetic procedures for MIL-100(Fe) exploit solvothermal conditions, high temperatures, and the presence of hydrofluoric acid as a modulating agent, inducing the formation of single crystals.[S1–S3] These conditions can yield highly crystalline

materials, although it has been shown that HF leaves behind fluoride ions that are able to coordinate iron centers in the triiron building unit of MIL-100(Fe). This hampers the formation of active Fe(II) species essential for the methane to methanol catalytic reaction.[S4] Conversely, only hydroxide ions and water molecules bind the iron centers when HF-free syntheses are employed, and these can be simply removed by applying a thermal gradient to the material. Unluckily, the absence of the acid medium typically results in lower crystallinity of the synthesized material preventing a detailed crystallographic characterization of the system. To circumvent this problem, we employed a recently reported reconstruction procedure with slight modifications, exploiting a water-mediated re-crystallization of the framework.[S5]

All reagents were obtained from commercial suppliers and used as received. 1.4454 g of $Fe(NO_3)_3{\cdot}9H_2O$ (3.578 mmol) and 0.4932 g of H_3BTC (H_3BTC = benzene-1,3,5-tricarboxilic acid, 2.35 mmol) were mixed in an agate mortar and manually ground for 20 min. The resulting white powder was then annealed at 160°C for 4 h in an oven using a stainless steel autoclave (Parr instruments). After cooling to room temperature, the dark-brown product was collected and thoroughly washed with methanol to remove any unreacted components. The sample was then recovered by centrifugation and dried at 30 °C in air, yielding an orange powder of as-synthesized MIL-100(Fe), hereafter referred to as AS-MIL-100(Fe).

The reconstruction process of the AS-MIL-100(Fe) sample was carried out following Souza et al. with slight modifications:[S5] the AS-MIL-100(Fe) sample was immersed in ca. 30 mL of distilled water and stirred at room temperature for 7 hours. The material was then recovered via centrifugation and dried at room temperature. This procedure was repeated for two times, that is until the obtained material showed a significantly improved degree of crystallinity form PXRD analysis (vide infra). During the reconstruction process a change in the powder color is also observed, the final color being salmon pink after two washing steps. The final mass of the reconstructed MIL-100(Fe) sample was 0.750 g, (1.15 mmol, 32.14% yield based on $Fe(NO_3)_3{\cdot}9H_2O$). An additional reconstruction experiment was also carried out on the exhaust sample recovered from the X-ray absorption/diffraction experiment (vide

infra), again stirring the powder for 7 h in water and recovering the powder by centrifugation and subsequent drying in air.

1.2 Preliminary PXRD Characterization

Gently ground powders of MIL-100(Fe) were deposited in the 2 mm deep hollow of a zero background plate (a properly misoriented quartz monocrystal). Powder X-ray diffraction (PXRD) patterns were collected using Cu-Kα radiation (λ=1.5418 Å) on a vertical-scan Bruker AXS D8 Advance diffractometer in θ:θ mode, equipped with a Goebel Mirror and a Bruker Lynxeye Linear Position Sensitive detector, with the following optics: primary and secondary Soller slits, 2.3° and 2.5°, respectively; divergence slit, 0.1°; receiving slit, 2.82°. The generator operated at 40 kV with a 40 mA current. The nominal resolution for the present set-up is 0.08° 2θ (FWHM of the α1 component for the LaB_6 peak at about 21.3° 2θ). A variable temperature powder X-ray diffraction (VT-PXRD) analysis was also performed to confirm the structural stability of the material in air up to 310 °C. This was done using a custom-made sample heater (Officina Elettrotecnica di Tenno, Ponte Arche, Italy) plugged in a Bruker AXS D8 Advance diffractometer, with the following procedure: $\sim$ 20 mg of the compound were deposited in an aluminum sample-holder and heated in air from 30 °C up to 310 °C. Data were collected with a narrower temperature step in the 160-210 °C range, where the self-reduction process and the formation of Fe(II) species is expected. PXRD patterns were acquired covering a sensible low-to-medium-angle 2θ range (1.8-30.0°, Cu-Kα). Le Bail refinements were carried out using the TOPAS-Academic 6 software.[S6–S8] The peak shapes were described with the fundamental parameters approach.[S9] The background was modelled by a Chebyshev polynomial function.

The powder X-ray diffraction patterns collected at each step of the reconstruction procedure show an increase in the diffraction intensity of the typical Bragg peaks observed for MIL-100(Fe). This suggests an increase in the long-range order of the bulk material and of its crystallinity while proceeding with the reconstruction treatment in water (see Figure S1).

A structureless Le Bail fitting of the experimental pattern of the reconstructed MIL-100(Fe) is reported in Figure S2, confirming its phase purity.

Figure S3 shows the VT-PXRD patterns collected from 30 °C up to 310 °C. Notably, the crystallinity of the sample is not affected up to the essayed temperature, even if the sample undergoes the known desolvation and self-reduction processes. This confirmed the thermal and structural stability of the framework. Whole powder pattern refinements using the Le Bail method were performed on each pattern acquired in the range 30-210 °C to describe the behaviour of the unit cell parameters as a function of temperature. The obtained values of the refined unit cell parameters and the figures of merit of the refinements are reported in Table S1, while Figures S4a and b show the temperature evolution profile of the unit cell volume and a axis, respectively. Figure S4c instead shows the percentage variation of the unit cell parameters (p_T) with respect to their initial values determined at 30 °C (p_i). The results show a contraction of the unit cell volume in the range 30-150 °C. This shrinkage is fully reversible, as observed by the value of the unit cell parameters refined for the same sample cooled back to room temperature. This behavior is expected and related to the desorption/adsorption of a large amount of guest molecules (water) that can be hosted in the pores of MIL-100(Fe).

An additional laboratory-scale PXRD experiment was performed on the exhaust MIL-100(Fe) sample recovered from the operando X-ray absorption/diffraction experiment (vide infra). PXRD patterns were collected both on the post-reaction sample and on the same after reconstruction in water for 7 h. Figure S5 reports the results of this analysis comparing the PXRD pattern collected on the sample prior to synchrotron experiments (red trace), after the operando experiment (light blue trace), and the same after reconstruction (blue trace). This comparison confirms that no significant amorphization of the MIL-100(Fe) sample occurred in our conditions and that the MOF catalyst can be regenerated by water reconstruction affording similar crystallinity as the pristine sample after only one washing step.

1.3 Infrared Spectroscopy Measurements

Fourier transform infrared (FTIR) spectra were acquired over the 4000–400 cm^{-1} range in attenuated total reflectance (ATR) mode on a diamond crystal by means of a Perkin-Elmer Paragon 1000 spectrometer. A few milligrams of each sample (previously dried in air at room temperature) were put on the diamond crystal of the ATR-FTIR spectrometer, and four scans were collected and averaged.

Figure S6 shows the comparison between the AS-MIL-100(Fe) sample (red trace), with the same after a single reconstruction step (light blue trace) and with the sample obtained after two subsequent reconstruction steps (blue trace). Signals at 1374 cm^{-1} ($\triangle$ mark) can be assigned to the stretching of O=C=O moieties, while the signal at 1710 cm^{-1} (* mark) is due to the stretching of protonated carboxyl groups. The band centered at 1290 cm^{-1} ($\diamondsuit$ mark) is instead assigned to the presence of a small amount of unreacted ligand in the annealed powder. During the reconstruction procedure, the IR band centered at 1710 cm^{-1} decreases in intensity, indicating a decrease in free carboxylic acid groups present both in defects of the MOF structure and in unreacted H_3BTC. This happens because water promotes the deprotonation of carboxylic acid groups in MIL-100(Fe) as well as the reaction of H_3BTC with iron species to form MIL-100(Fe). For the same reasons, the band at 1290 cm^{-1} essentially disappears from the IR spectrum after the first soaking in water and the band at 1374 cm^{-1} becomes sharper after each washing step. The region relevant for O-H stretching modes of the hydroxyl groups shows a broad signal centered at 3200 cm^{-1} with low-intensity sharp peaks at ca 3000 and 3120 cm^{-1}, assigned to the -OH groups capping one of the Fe(III) metal centres in the trimeric inorganic units. The broad band observed in this region is also attributed both to the coordinated water molecules and the ones adsorbed in the pores.

1.4 Thermogravimetric Analysis

Thermogravimetric analysis (TGA) was carried out on a Perkin-Elmer TGA 7 thermogravimetric analyzer. 1.115 mg of the MIL-100(Fe) sample were placed on an alumina pan and heated under a nitrogen flow (40 mL min^{-1}). The heating ramp was set to 10 °C min^{-1}, from 30 to 447 °C. Figure S7a shows the results of the TGA profile. The continuum mass loss in the 30-200 °C range can be attributed to the desorption of water molecules, while degradation of the material is observed above 250 °C.

1.5 N_2 Adsorption Measurements

N_2 sorption measurements were carried out on the fully reconstructed MIL-100(Fe) sample with a a Micromeritics ASAP 2020 instrument at -196 °C and up to 1 bar. Prior to measurement, the powdered sample was outgassed at 180 °C and 10^{-6} Torr for 12 h. The calculated Brunauer-Emmett-Teller (BET) surface area was 1176.92 m^{2}g^{-1}. Figure S7b shows the N_2 adsorption isotherm collected at 77 K on MIL-100(Fe).

1.6 Operando HERFD-XAS, XAS-XRD, XES and RIXS Experiments

Operando high energy resolution fluorescence detected X-ray absorption spectroscopy (HERFD-XAS), 1s3p RXES and valence-to-core (VtC) XES measurements were performed at beamline ID-26 of the European Synchrotron Radiation Facility (ESRF) with the storage ring operating at 6 GeV and injection currents of 190 mA in multibunch mode. A double crystal Si(111) monochromator was used to select incident beam energy. The incident energy was calibrated by setting the first inflection point of a Fe foil to 7111.2 eV. The X-ray emission measurements were performed using a Johann-type spectrometer equipped with an array of five spherically bent Ge (620) analyzer crystals aligned in a Rowland geometry and combined with a silicon drift diode detector.[S10] The spectrometer and monochromator have an aver-

aged combined resolution of $\sim$ 1.0 eV. About 50 mg of a mixture of MIL-100(Fe) and boron nitride (BN) at a 1:4 MOF:BN ratio were pressed into a wafer and lodged into a reactor cell connected to the beamline gas lines. Radiation damage was assessed by determining the dwell time per sample spot through the collection of short energy range XAS scans of 10 sec/scan. The dwell time per sample spot avoiding radiation damage effects was employed for all experiments. HERFD-XAS spectra were collected in the energy range between 7100-7150 eV in 0.1 eV steps and subjected to a smoothing procedure using the Savitzky-Golay filter (polynomial order: 3, window size: 11 points).[S11] Resonant Kβ and VtC XES measurements were collected in energy ranges of 7030-7068 eV and 7060-7120 eV, respectively, in 0.2 eV steps. The incident energies for the 1s3p RXES measurements (7113.7, 7115.1 and 71124.5 eV) during the thermal activation of the pristine MIL-100(Fe) sample were selected based on the HERFD-XAS measurements.

Operando XAS-XRD measurements were performed at BM31 beamline (Swiss-Norwegian beamlines, SNBL) at the ESRF synchrotron. The ring was operating at 6 GeV with a current of 190 mA in multibunch mode. The XAS spectra were collected at the Fe K-edge continuously scanning between 7000 and 7900 eV with a step size of 0.3 eV using a Si(111) double crystal monochromator, and the beam was calibrated using a Fe foil assigning the first inflection point to 7111.2 eV. The PXRD patterns were collected with a 2D DEXELA detector[S12] at a wavelength of 0.3386 Å using a Si(111) channel-cut monochromator. Calibration was performed with NIST LaB_6 powder as a standard, and raw images were averaged and azimuthally integrated with the pyFAI software.[S13] XAS spectra and PXRD patterns were alternately recorded by automatically switching between the DEXELA detector and the XAS-dedicated ion chambers. About 50 mg of a mixture of MIL-100(Fe) and boron nitride (BN) at a 1:4 MOF:BN ratio were pressed into a wafer and then disrupted, sieving out the fraction with size above 125 μm. About 5 mg of the resulting powder were loaded in a 1 mm (0.02 wall thickness) quartz capillary reactor, plugged with quartz wool and hooked to the gas lines on the BM31 sample stage. The sample was first heated to 250 °C at 1 atm

in a 12 mL/min He flux, then the activated sample was cooled to 200 °C in He and exposed to a mixture of 12 mL/min He, 3mL/min CH_4 and 3 mL/min O_2 flux at 1 atm to perform the methane to methanol (MTM) reaction. The spent catalyst was subjected to one more cycle of activation at 250 °C in He, reaction at 200 °C with CH_4 and O_2, and reactivation at 250 °C in He demonstrating excellent cyclability (see main text). The sample was then reactivated at 250 °C once again, and exposed to CH_4 and O_2 at 100 °C, using the same fluxes as above. Finally, the activated sample was exposed to O_2 and CH_4 at 25 °C using the previously reported fluxes. In a different experiment, the sample was activated again at 250 °C in He and pure O_2 (6 mL/min), pure CH_4 (6 mL/min), and a mixture of O_2 and CH_4 (3 mL/min each) were each consecutively fluxed at 200 °C to assess the interaction with these species. Afterwards, a final activation at 250 °C in He was carried out to further verify the regeneration capacity of the MOF. A final experiment to compare the MTM reactivity of MIL-100(Fe) with N_2O and O_2 was carried out by exposing the MOF to a mixture of CH_4 and N_2O (3 mL/min each) at 200 °C and ambient pressure following activation at 250 °C in He as previously described. XAS and XRD measurements were collected during each reaction and activation step to monitor the state of the sample. A preliminary experiment was also performed using the same setup at the BM31 beamline, with an increased sample mass (∼20 mg of undiluted sample) to augment the possible methanol production without collecting XAS/XRD data. About 20 mg of MIL-100(Fe) were loaded in a 2 mm quartz capillary reactor with 0.01 wall thickness. In this case the He flux was 10 mL/min during the activation ramp and the reaction was carried out at 200 °C in a 5 mL/min O_2 and 5 mL/min CH_4 flux with no He. During all experiments, the outlet gas was fed to a Pfeiffer Omnistar GSD 301 C mass spectrometer to detect and monitor methanol production. Finally, Figure S8 presents a structureless Le Bail refinement of PXRD pattern collected on the sample collected at the synchrotron before thermal treatment. The evolution of the PXRD patterns recorded during the thermal treatment together with the XAS data, is shown in Figure S9.

2 Data Analysis

2.1 Determination of the Fe(II) Content

To estimate the Fe(II) content in the MIL-100(Fe) sample during each reaction/reactivation cycle, a linear correlation was established between the energy position at 0.925 normalized intensity in the XANES spectra of FeO and Fe_2O_3 and their respective Fe(II) content (100% and 0%, see Figures 3a and 3b of the main text). The resulting equation was y = -30.22x + 215396.78, where x is the energy position and y the Fe(II) percentage. The associated error was estimated by deriving the same linear correlation for all values in the 0.90 - 0.95 normalized intensity range and using each obtained equation to calculate the Fe(II) content present in the sample activated at 250 °C (see Figure S12). Since the Fe(II) values calculated linearly decrease from 19.7% to 14.9% when performing the calculation at 0.95 and 0.90 normalized intensity, respectively, we assume an error of ±5% for all the percentages we report.

2.2 Decomposition of the HERFD-XAS Dataset into the Spectra and Fractional Concentration of the Key Components

The HERFD-XAS experimental data was decomposed into the spectra and relative concentrations of the key species present in the reaction mixture employing the PyFitit code.[S14] Specifically, a transformation matrix-based approach belonging to the family of the multivariate curve resolution (MCR) family methods was used to retrieve the spectral and concentration profiles of the key components, whose number was established to be equal to $N = 3$ by means of the scree plot statistical test (see Figure 3d of the main text).[S14] In the analysis, two of the XANES normalized spectral components were constrained to coincide with the Fe K-edge XANES spectra of the as-synthesized and activated MIL-100(Fe) samples. This procedure allowed us to reduce the number of unknown matrix elements from N^2 to $N^2 - 2N$.

2.3 XAS and VtC-XES Calculations

The XANES and VtC-XES theoretical data analysis was performed with the FDMNES code using the Finite Difference Method (FDM). FDMNES originally employs a one-electron approach and is based on the *ab-initio* calculations of the electronic structure and further resolution of the discretized radial Schrödinger equation avoiding the muffin-tin approximation.[S15] Herein, the Hedin-Lundqvist formulation was used to derive the energy dependent exchange-correlation potential.[S16] In addition, the sparse solver method for the finite difference matrix was implemented.[S15,S17,S18] Simulations of XANES and VtC-XES spectra were performed including spin-orbit coupling, quadrupolar transitions, and self-consistency in the calculations. For all spectral simulations, a cluster radius of 6 Å centered on each Fe absorbing species of the trimeric MIL-100(Fe) unit was employed and the calculations were based on the available MOF crystal structure (see Figure S13).[S19] The valence electronic configuration of the Fe(III) metal sites in the pristine and partially hydroxylated MOF was set to $3d^5 4s^0 4p^3$, a procedure that properly describes the Fe(III) d-orbital character while conserving the system charge neutrality.[S15] A gaussian broadening of 2.5 eV was applied to the VtC-XES theoretical spectra to account for the experimental resolution.

3 Density Functional Theory Calculations

Density functional theory (DTF) calculations were carried out at the unrestricted M06-L/def2-TZVP[S20–S22] level of theory with the ORCA 5.0.2 quantum chemistry program,[S23] using a model for the trimeric nodes of MIL-100(Fe) where the BTC ligands are represented by formate ions. A depiction of this model is reported in Figure S35. This combination of functional, basis set and model has been benchmarked to the performance of multireference calculations and that of the corresponding model including phenyl rings,[S24,S25] and numerous works have employed it successfully to study the properties of MOFs that present this type of Fe_3O unit including their reactivity towards the MTMconversion.[S24–S29] The diagonalization-

free KDIIS converger was employed, invoking the approximate second order self-consistent field (SOSCF) method[S30] at low orbital gradient values to improve convergence. The resolution of identity (RI) approximation with an automatically constructed auxiliary basis set[S31] (corresponding to the AutoAux setting) was used for Coulomb integrals to reduce the computational cost. All calculations were performed on both the 2S+1=17 and 2S+1=15 spin surfaces to determine at which point of the reaction coordinate the spin transition between the reagents (O_2, Σ_g^3) and the products (methanol, a closed shell molecule) occurs. It is in fact mandatory to determine the reaction path on multiple spin surfaces in order to account for the fact that the O_2-based MTM conversion is formally spin-forbidden, an issue often regarded as the "spin dilemma" of this reaction.[S32] A geometry optimization was first carried out on all reactants, products and intermediates of the MTM reaction. A vibrational analysis was performed to ensure that the structures present no significant (<-30 cm^{-1}) negative frequencies and correspond to minima in the potential energy surface. The energy of every structure was corrected for the basis set superposition error (BSSE) following the method proposed by Boys and Bernardi,[S33] and BSSE was found to be lower than 6 $kJmol^{-1}$ for all structures. Thermochemistry at 298 K and 1 atm was also computed for every structure to construct the reaction enthalpy and Gibbs free energy diagrams, using the conventional statistical thermodinamics partition functions (ideal gas, particle in a box, rigid rotator and harmonic oscillator), with a cutoff value of 50 cm^{-1} for low frequency vibrations.

Subsequently, transition states **TS1** and **TS2** were localized with a combination of the nudged elastic band (NEB) algorithm and an eigenvector-following transition state search,[S34,S35] referred to as the NEB-TS method in the ORCA implementation.[S36] In this procedure, a climbing-image NEB (CI-NEB) optimization is first performed with loose convergence settings.[S37] A set of structures (the so-called "images") connecting the two intermediates is generated by simple interpolation of the atomic coordinates and a single-point energy and gradient calculation is carried out of on each image. The gradient is used to minimize the energy of all images following a direction perpendicular to the minimum energy

reaction path, while the highest energy image (the "climbing image") is also pushed uphill along the path towards the saddle point. When convergence is achieved, the CI-NEB procedure is halted and the climbing image is used as a starting guess for an eigenvector-following optimization to accurately define the transition state geometry. The number of NEB-TS images was equal to 12 for each pair of intermediates and the NEB-TS calculations were performed at the same level of theory as that employed for the optimizations. Transition states **TS3** and **TS4** have already been well characterized in the literature,[S24,S27] hence they have been localized with a conventional eigenvector-following transition state search.

The desorption electronic energy, enthalpy and Gibbs free energy of the $CH_3^\bullet$ radical from intermediates **C** and **F** (see main text) was determined starting from the already optimized structures of both intermediates on the 2S+1=15 spin surface, removing the radical and optimizing the resulting structures. The energetic parameters of the $CH_3^\bullet$-free structures was then added to that of a free $CH_3^\bullet$ radical to calculate the desorption electronic energy, enthalpy and Gibbs free energy according to the following equation:

$$\Delta X^{0c}_{des,CH_3^\bullet} = X^0_{\mathbf{C},\mathbf{F}} - (X^0_{\mathbf{C},\mathbf{F}-CH_3^\bullet} + X^0_{CH_3^\bullet}) + BSSE \tag{1}$$

where X indicates E, H and G.

To compare the enthalpy and Gibbs free energies of the two reaction stages, the enthalpy of one CH_4 molecule has been added to the enthalpy of species **B**, **TS1**, **C**, **TS2** and **D**. The same has been done with the enthalpy of one CH_3OH molecule for species **E**, **TS3**, **E**, **TS4** and **G**.

4 Supplementary Figures (Figures S1 - S41)

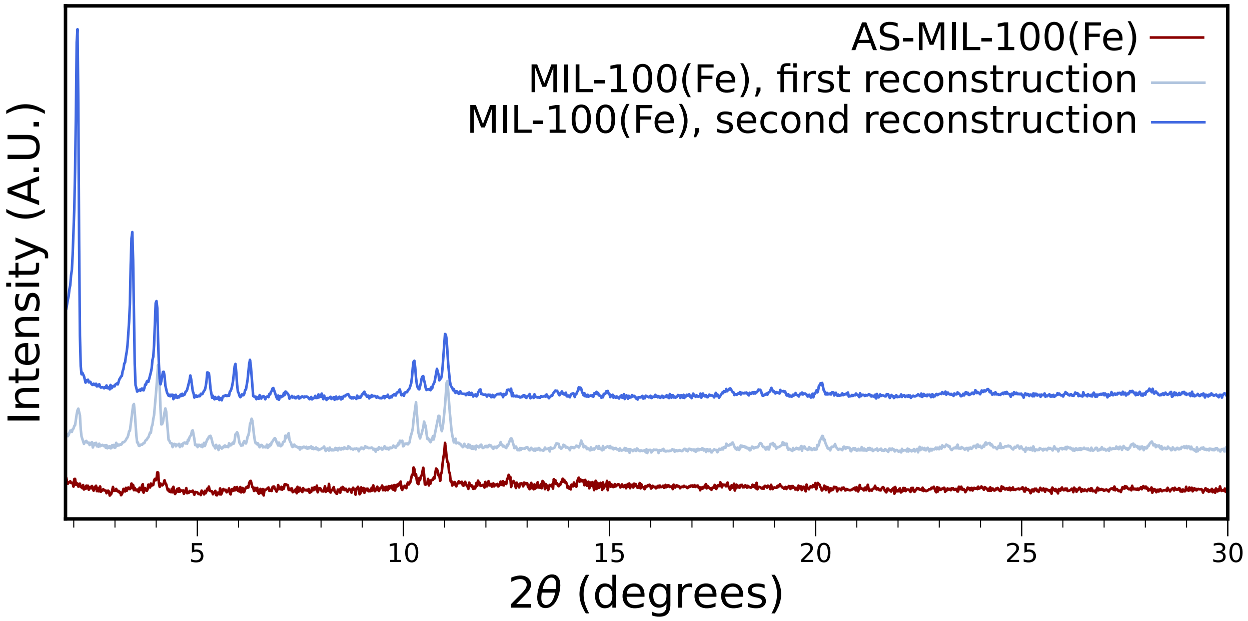

Figure S1: In-house PXRD patterns collected on the MIL-100(Fe) sample for each step of the water reconstruction procedure. As synthesized sample (red trace), sample after the first reconstruction step (light blue trace), and after the second reconstruction (blue trace). λ=1.5418 Å.

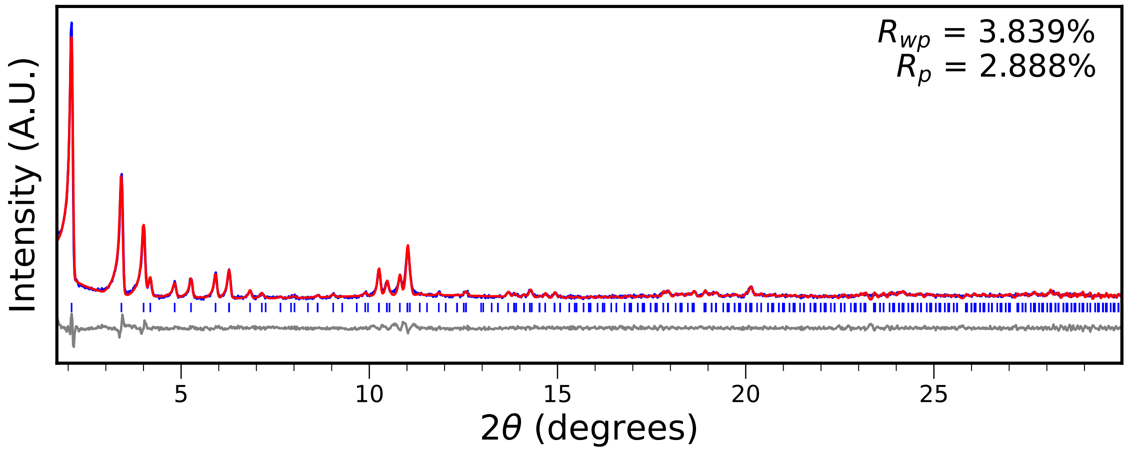

Figure S2: Whole powder pattern Le Bail refinement for the in-house PXRD pattern collected on the reconstructed MIL-100(Fe) sample. Experimental, calculated and difference traces are shown in blue, red and grey, respectively. The positions of the Bragg reflections are indicated with blue tick marks. Final figures of merit are reported in the plot. Space Group: Fd$\bar{3}$m, a=73.69 Å. λ=1.5418 Å.

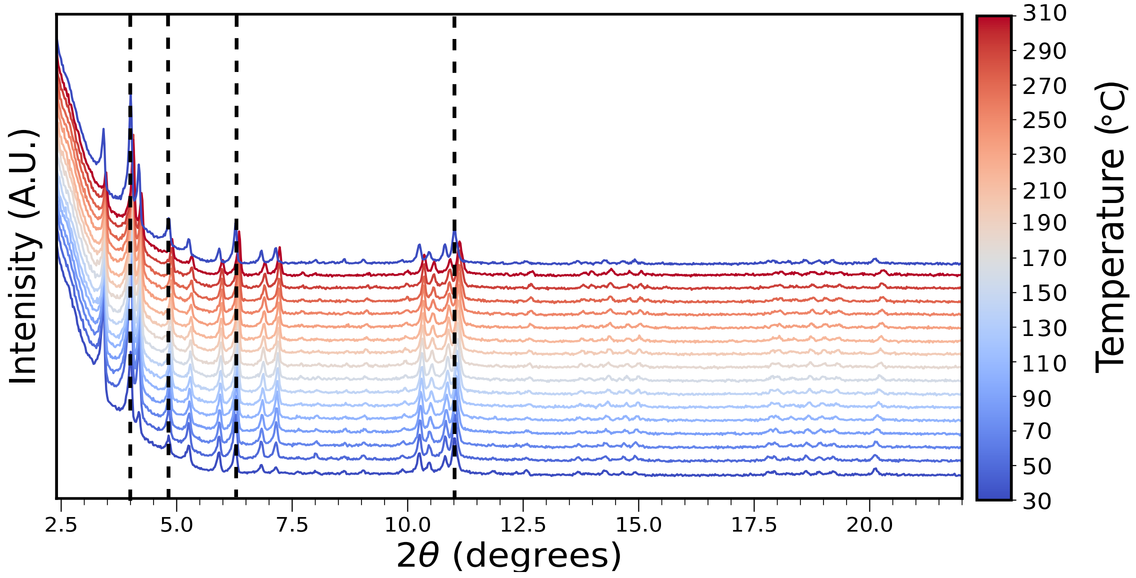

Figure S3: In-house VT-PXRD patterns of MIL-100(Fe) as a function of temperature in the range 30 (bottom trace, blue) – 310 (top trace, red) °C. The blue top trace represents sample cooled back to room temperature. λ=1.5418 Å.

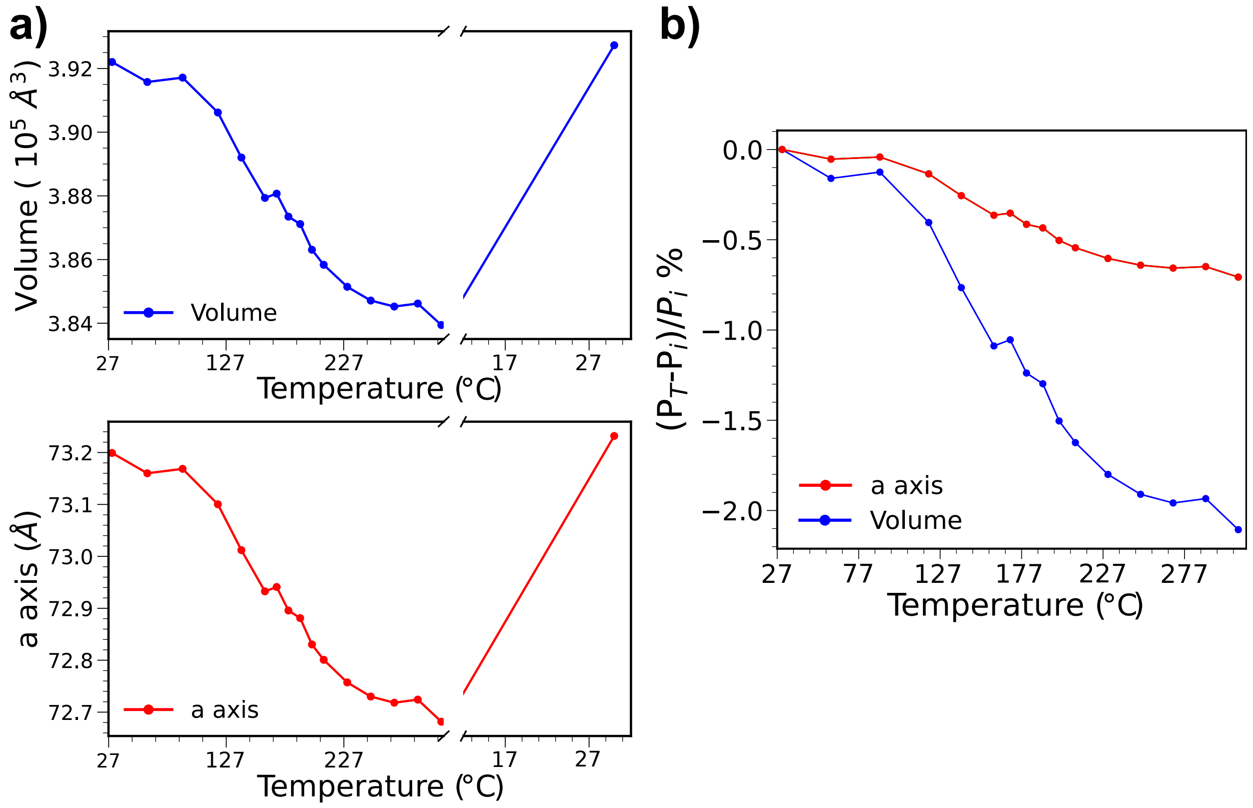

Figure S4: (a) Temperature evolution of the MIL-100(Fe) unit cell a axis (red) and cell volume (blue) during thermal treatment (T = 30-310 °C) and subsequent cooling (back to 30 °C) determined by the Le Bail refinement. (b) Temperature evolution of the MIL-100(Fe) unit cell parameters, P_T, normalized to the corresponding initial values (P_i) at T = 30 °C during thermal treatment (T=30-310 °C).

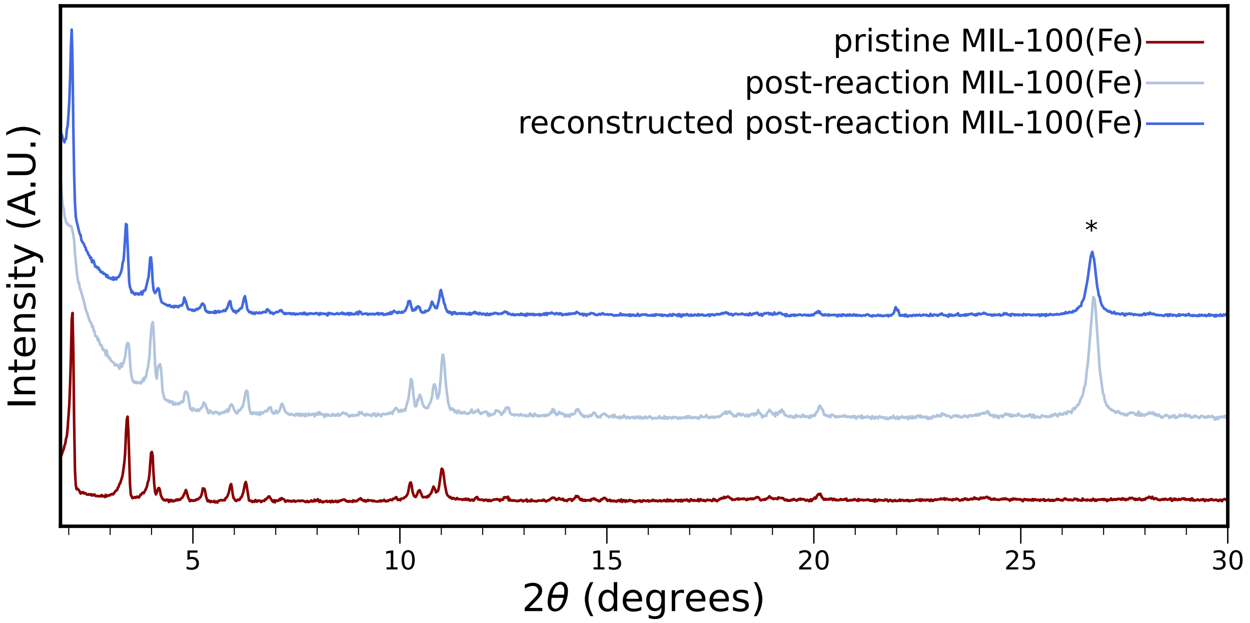

Figure S5: Comparison between the in-house PXRD patterns of pristine MIL-100(Fe), BN-diluted MIL-100(Fe) after the MTM process and of the same sample after the water reconstruction procedure (red, light blue and blue traces, respectively). The marked peak (*) is due to the BN phase used to dilute the sample. λ=1.5418 Å.

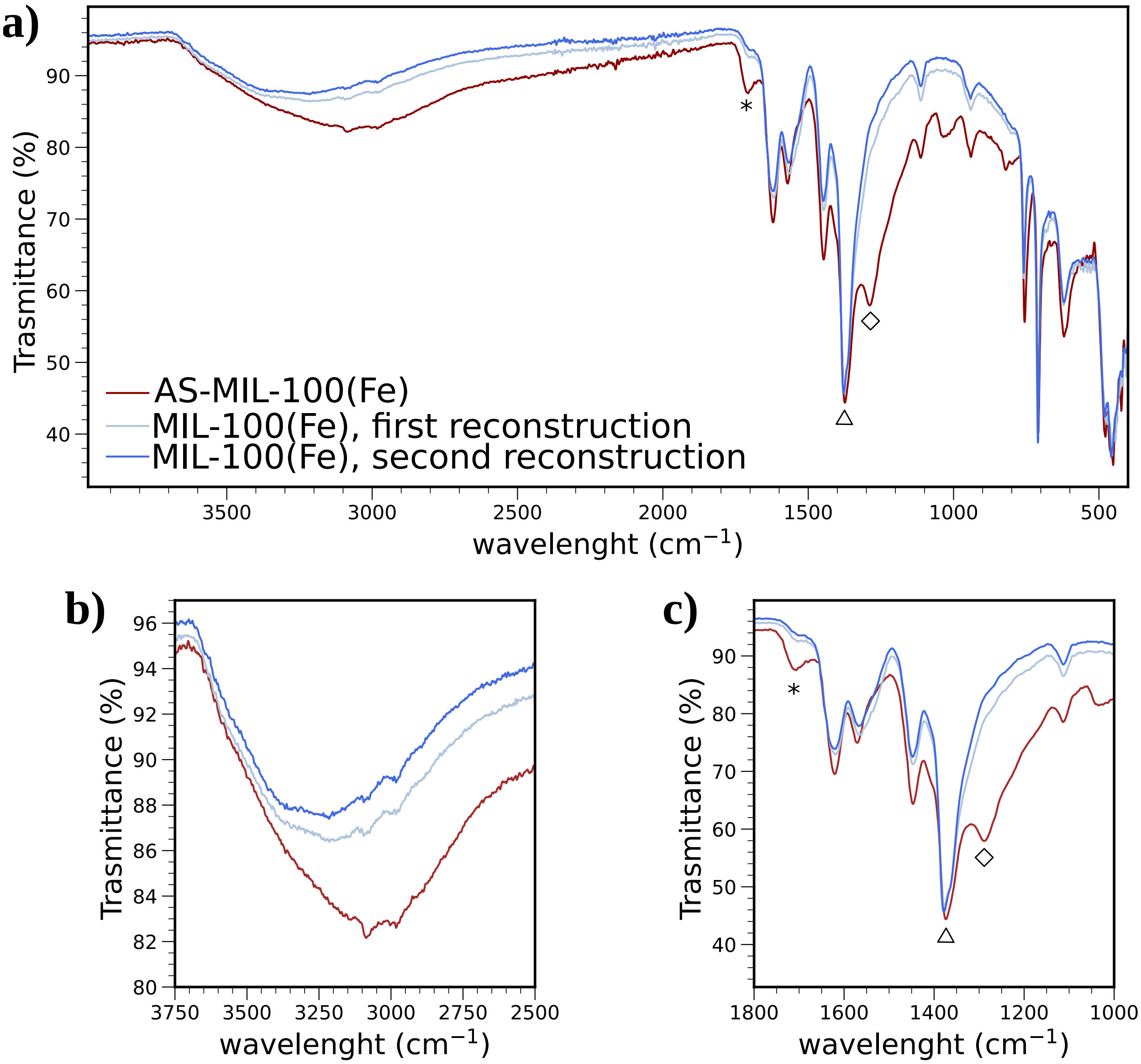

Figure S6: ATR-IR spectra of the MIL-100(Fe) sample for each step of the water reconstruction procedure. As synthesized sample (red trace), sample after the first reconstruction step (light blue trace), and after the second reconstruction (blue trace). a) Entire spectra of the samples. b) Magnification of the 3750-2500 cm^{-1} region in the ATR-IR spectra. b) Magnification of the 1000-1800 cm^{-1} region in the ATR-IR spectra. The bands highlighted at 1710 (* mark), 1374 (△ mark) and 1290 cm^{-1} (◇ mark) provide insight on the reconstruction process.

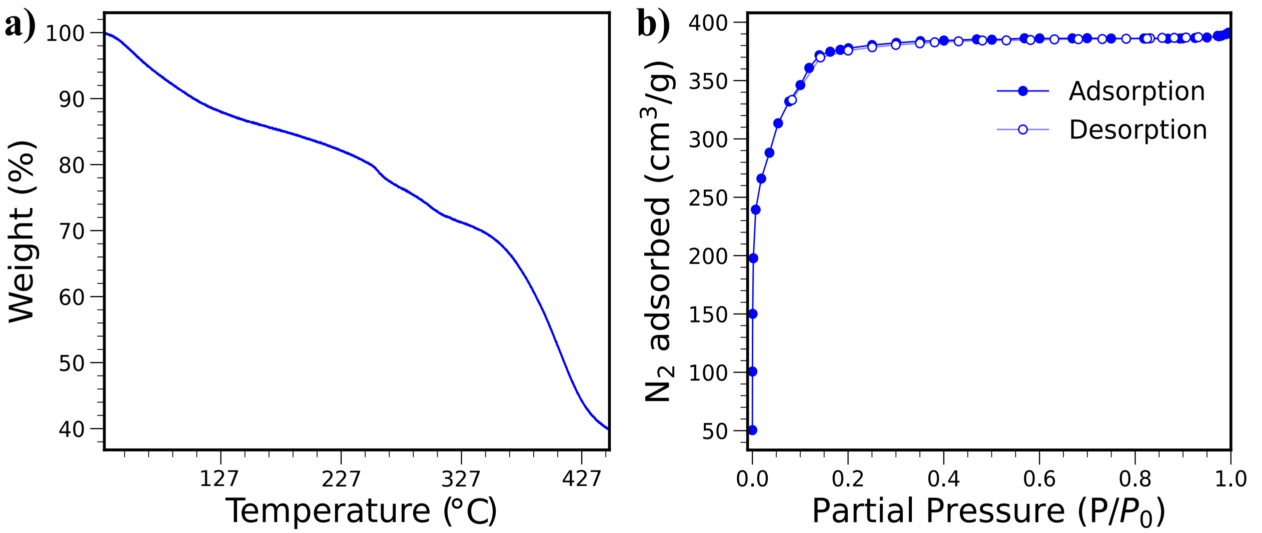

Figure S7: a) TGA profile of MIL-100(Fe) in the 303-720 K range. b) N_2 adsorption isotherm collected at -196 °C for MIL-100(Fe) activated under vacuum at 180 °C for 12h. BET surface area: 1176.92 m^2g^{-1}.

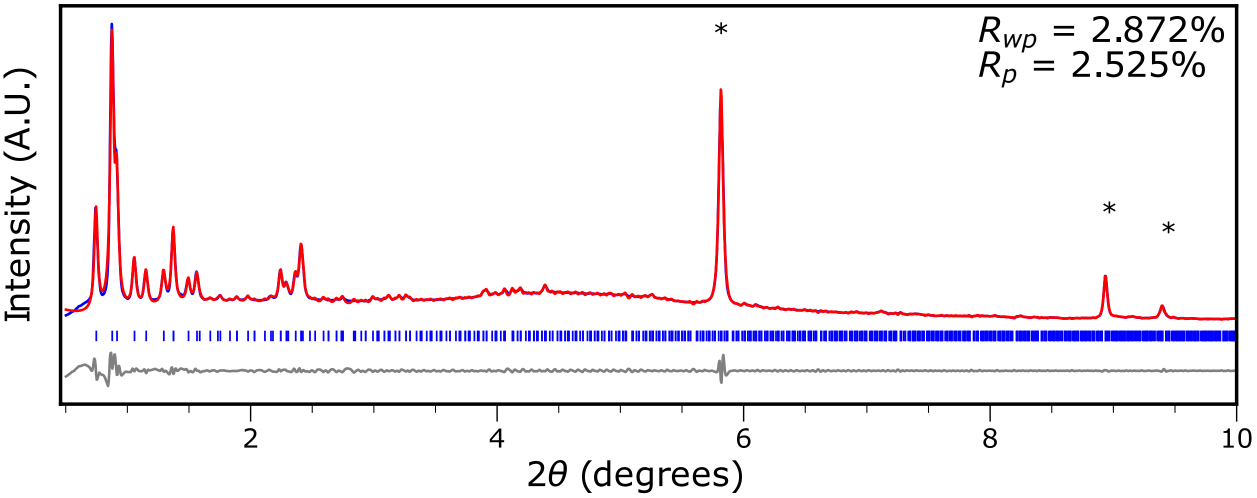

Figure S8: Whole powder pattern Le Bail refinement for the synchrotron PXRD pattern collected on the MIL-100(Fe) sample prepared for the operando experiments prior to thermal activation. Experimental, calculated and difference traces are reported in blue, red, and grey, respectively. Marked peaks at 5.8, 8.95, 9.41 2θ are due to the BN phase used to dilute the sample. Final figures of merit are reported in the plot. Space Group: Fd$\overline{3}$m, a=73.69 Å, λ=0.3385 Å.

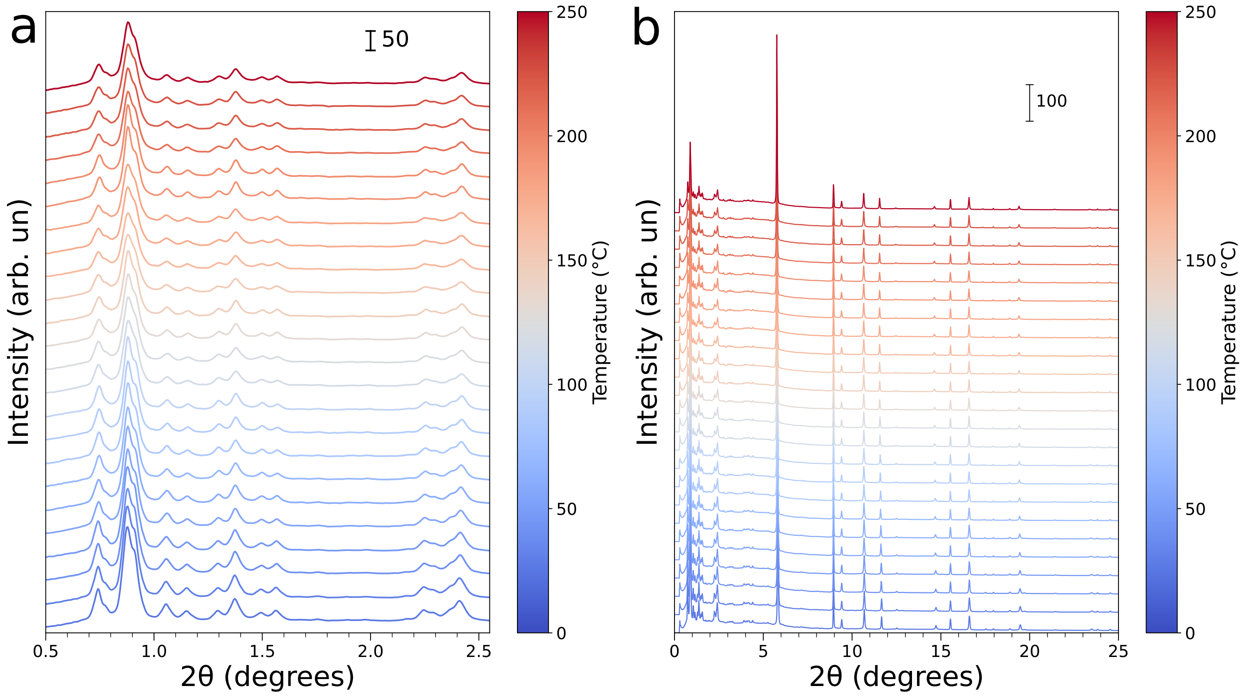

Figure S9: (a) Temperature evolution of the low angle region in the synchrotron PXRD patterns collected on the MIL-100(Fe) sample during thermal activation in He (from blue, 25 °C to red, 250 °C) (a), entire patterns (b). λ=0.3386 Å.

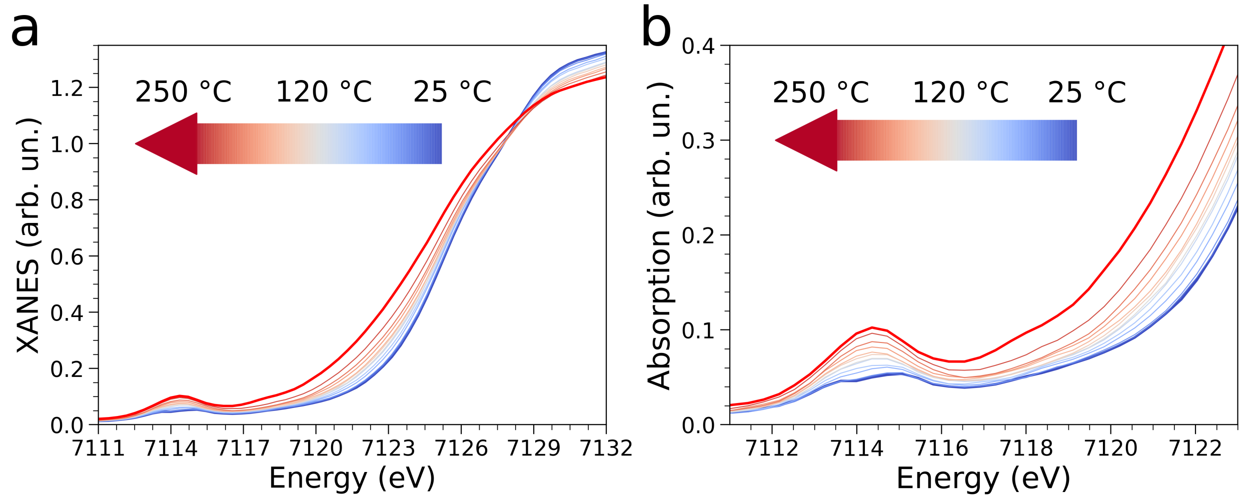

Figure S10: (a) Sequence of Fe K-edge XANES spectra collected during activation of the MIL-100(Fe) sample in He flux (blue, 25 °C, to dark red, 250 °C) and (b) magnification of the 7111.5-7122.5 eV region.

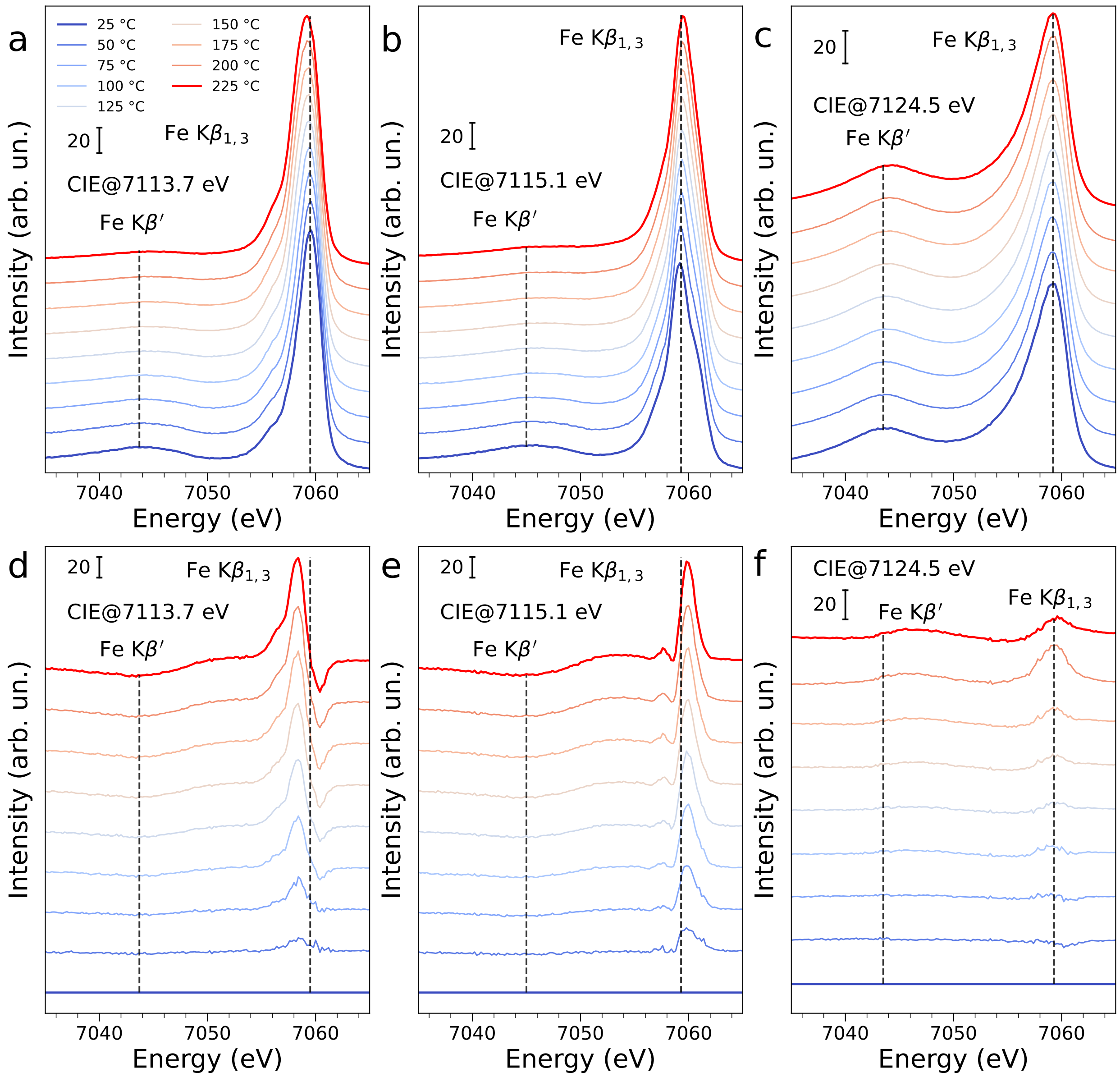

Figure S11: Fe K$\beta_{1,3}$ and Kβ' resonant XES spectra collected at different constant incident energies (CIE) of 7113.7 eV (a), 7115.1 eV (b) and 7124.5 eV (c) on the MIL-100(Fe) sample during thermal activation in He (from blue, 25 °C to red, 250 °C). Panels d, e and f show the difference between each spectrum and the one collected at 250 °C for each CIE. Dashed lines highlight the observed shifts in the K$\beta_{1,3}$ and Kβ' components.

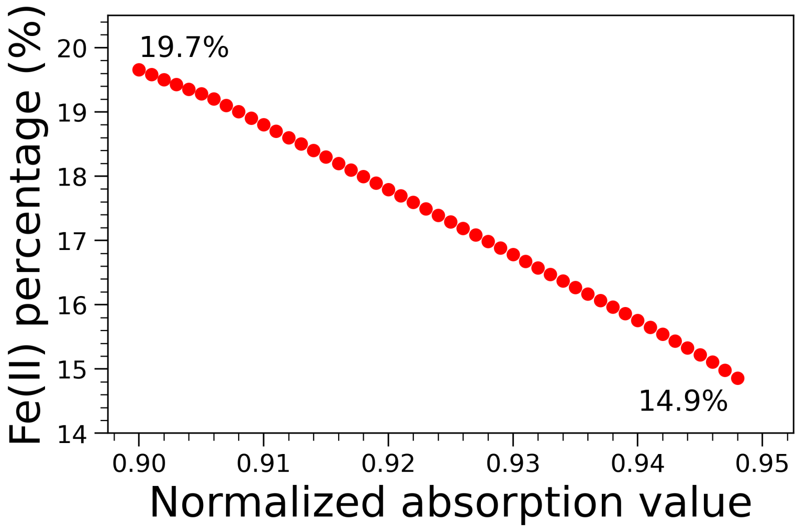

Figure S12: Fe(II) percentage as a function of the normalized intensity value at which the linear correlation is calculated. The spectrum of MIL-100(Fe) activated at 250 °C in He has been used to perform these calculations.

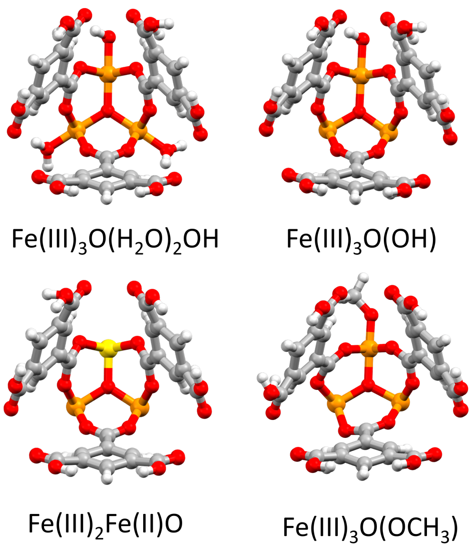

Figure S13: Clusters employed in the Fe VtC-XES and XAS calculations with the FDMNES program. Color code: Fe(III), orange, Fe(II), yellow, O, red, C, grey and H, white.

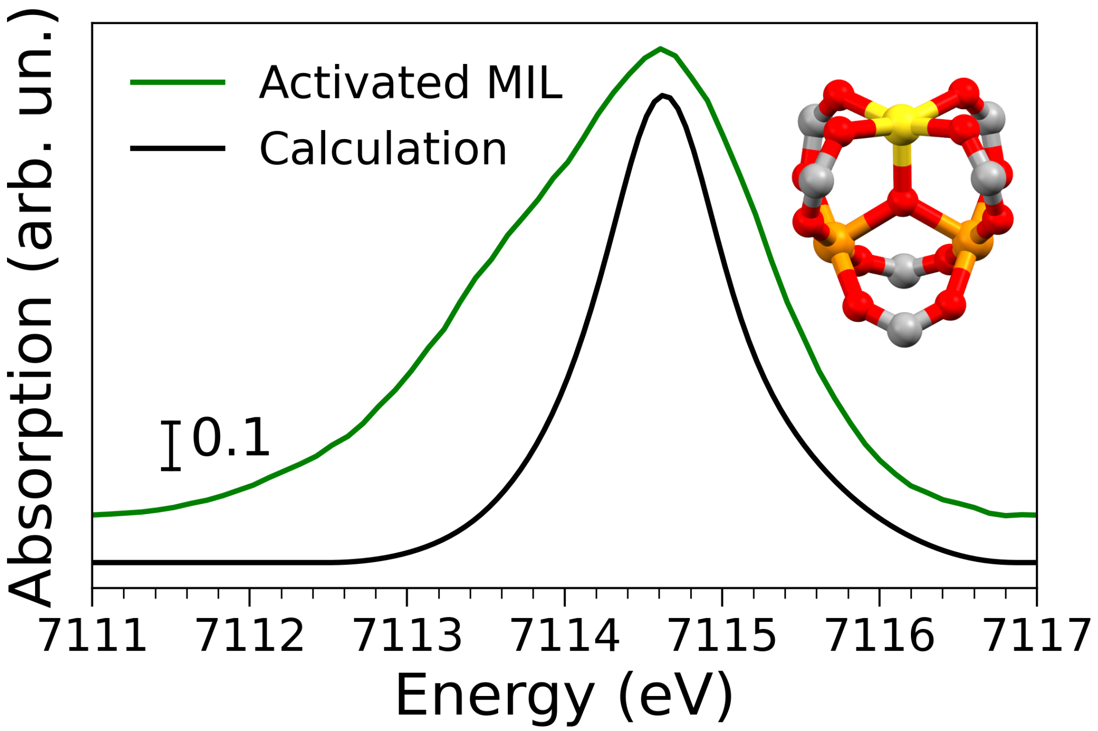

Figure S14: Comparison between the experimental Fe K-edge XAS spectrum of MIL-100(Fe) activated at 250 °C for 4 h, and the theoretical calculation performed with the $Fe(III)_2Fe(II)O$ cluster. The cluster employed in the calculation is depicted in the panel (Fe(III), orange, Fe(II), yellow, O, red, C, grey and H, white). The phenyl groups of the BTC ligands have been omitted for clarity.

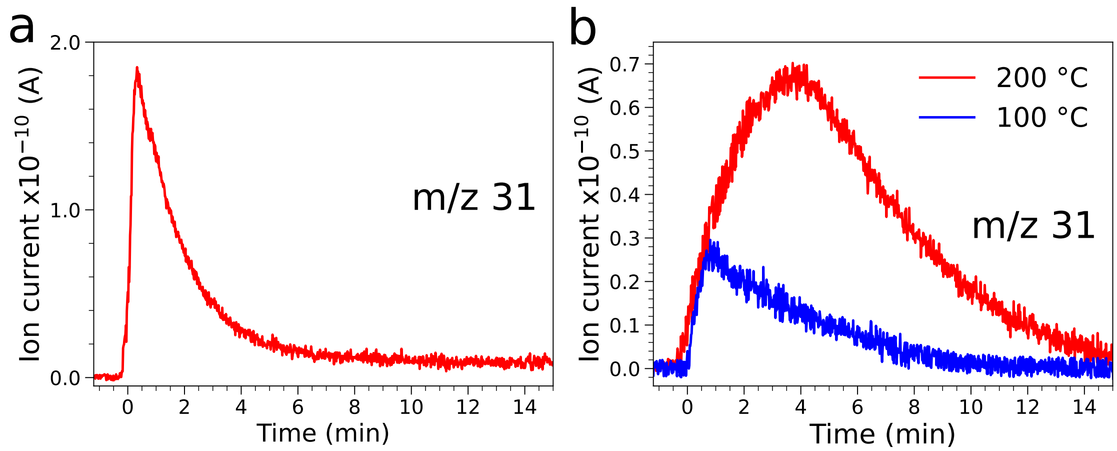

Figure S15: (a) Time evolution of the m/z=31 MS signal collected during a preliminary experiment on MIL-100(Fe) in a mixture of O_2 and CH_4 at 200 °C (red), using the same setup as for operando XAS but without collecting XAS data and employing a larger 2 mm-diameter capillary to increase the sample mass. (b) Time evolution of the m/z=31 MS signal collected during XAS experiments with a 1 mm capillary on MIL-100(Fe) in a mixture of O_2 and CH_4 at 200 °C (red) and 100 °C (blue).

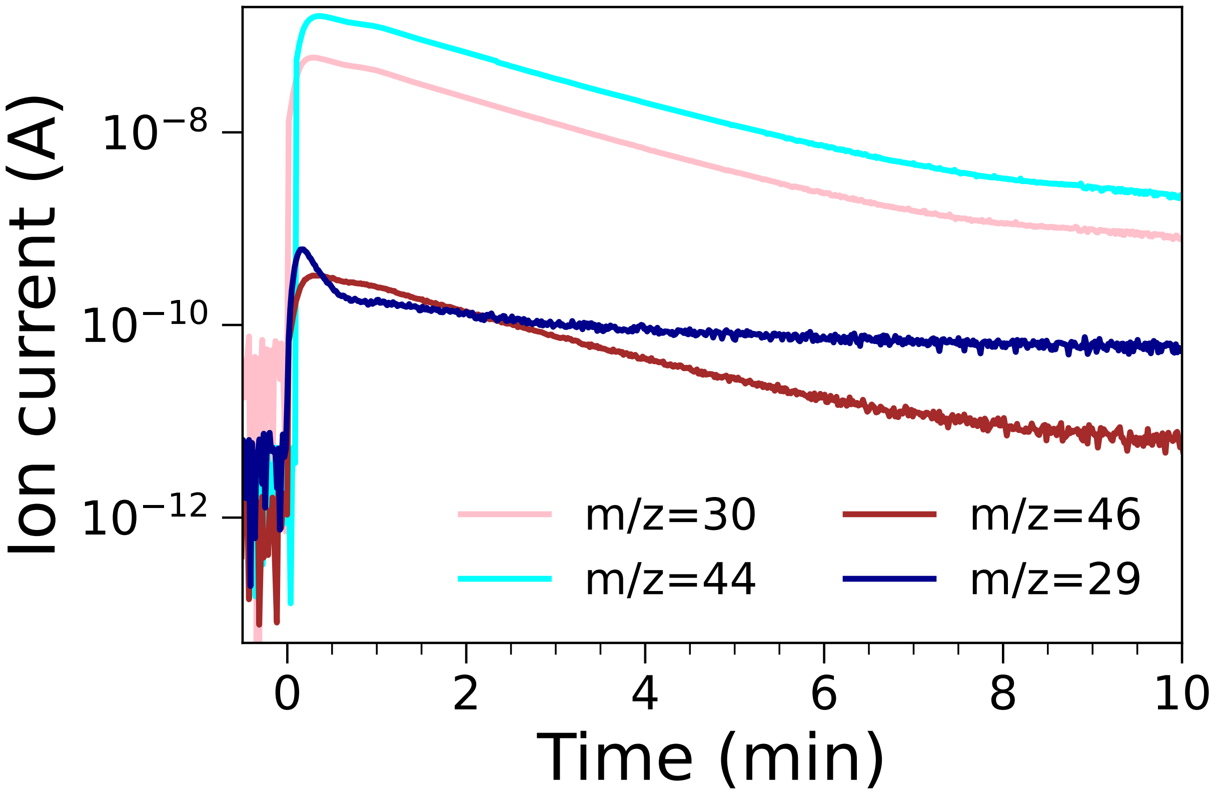

Figure S16: Time evolution of the m/z=30 (formaldehyde), m/z=44 (CO_2) and m/z=46 (dimethyl ether), and m/z=29 (associated both with methanol and all of its overoxidation products) MS signals collected during a preliminary experiment on MIL-100(Fe) in a mixture of O_2 and CH_4 at 200 °C, using the same setup as for operando XAS but without collecting XAS data and employing a larger 2 mm-diameter capillary to increase the sample mass. The ion current is reported in logarithmic scale.

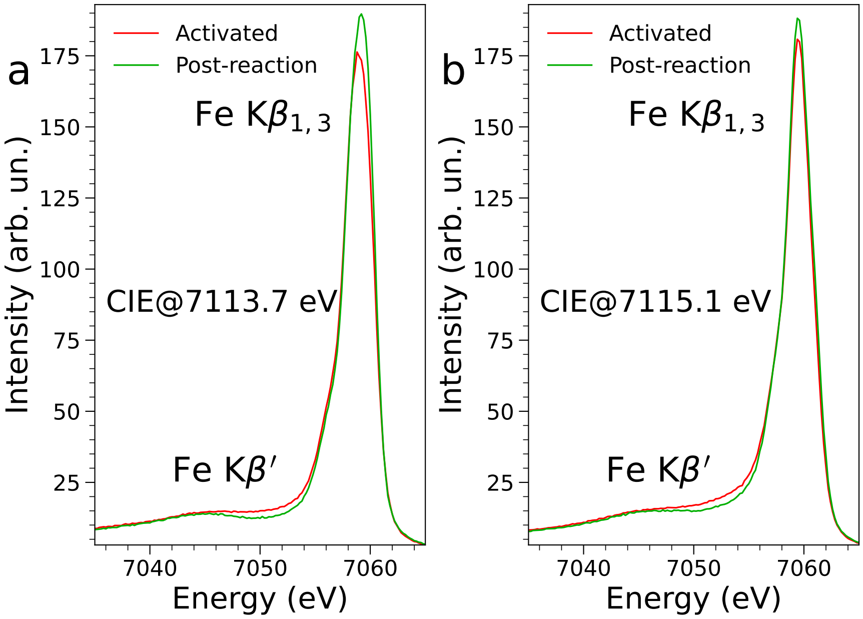

Figure S17: Fe K$\beta_{1,3}$ and Kβ' resonant XES spectra collected on the MIL-100(Fe) sample before (red) and after the reaction with a mixture of CH_4 and O_2 (green) at different constant incident energies (CIE): 7113.7 eV (a) and 7115.1 eV (b).

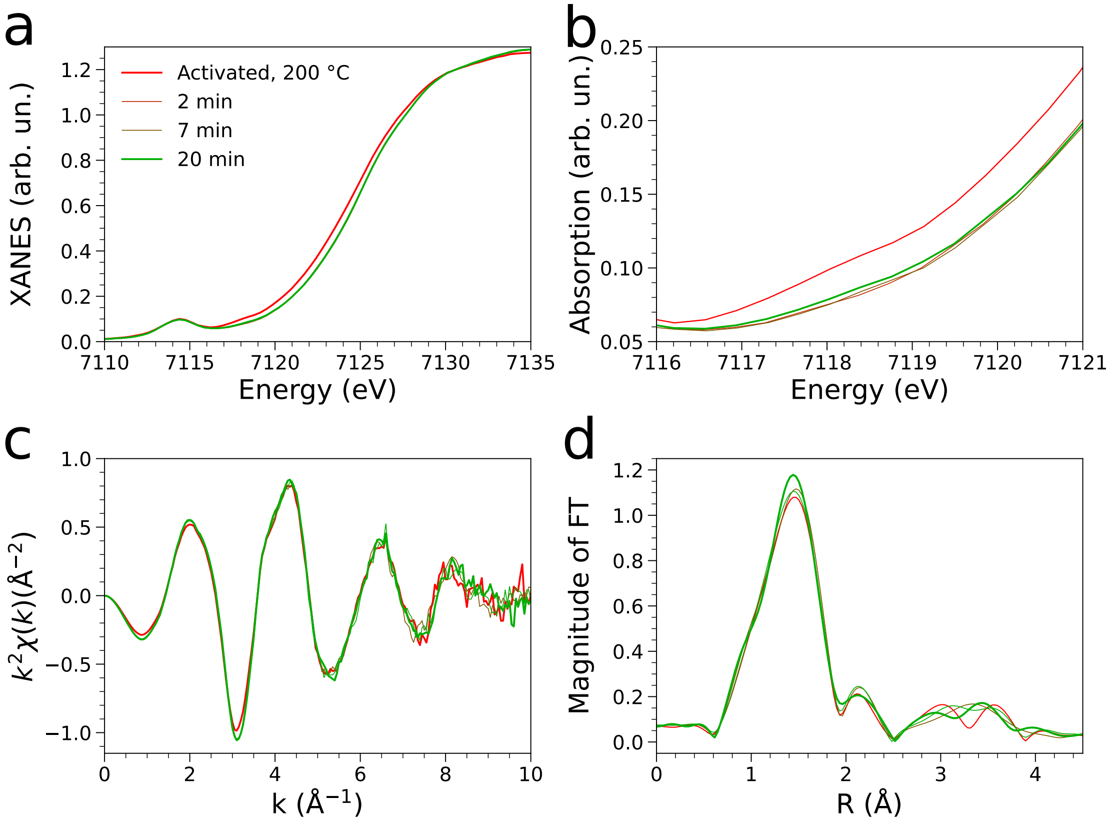

Figure S18: Sequence of Fe K-edge XAS spectra collected during reaction of the activated sample with a mixture of CH_4 and O_2 at 200 °C (first cycle). (a) XANES spectra collected during the reaction. (b) Magnification of the 7117-7122 eV region in the XANES spectra of panel a. (c) Evolution of the EXAFS signal ($k^2\chi(k)$) and (d) non-phase shift corrected FT magnitude of the $k^2\chi(k)$. The color code of all panels is the same as panel a.

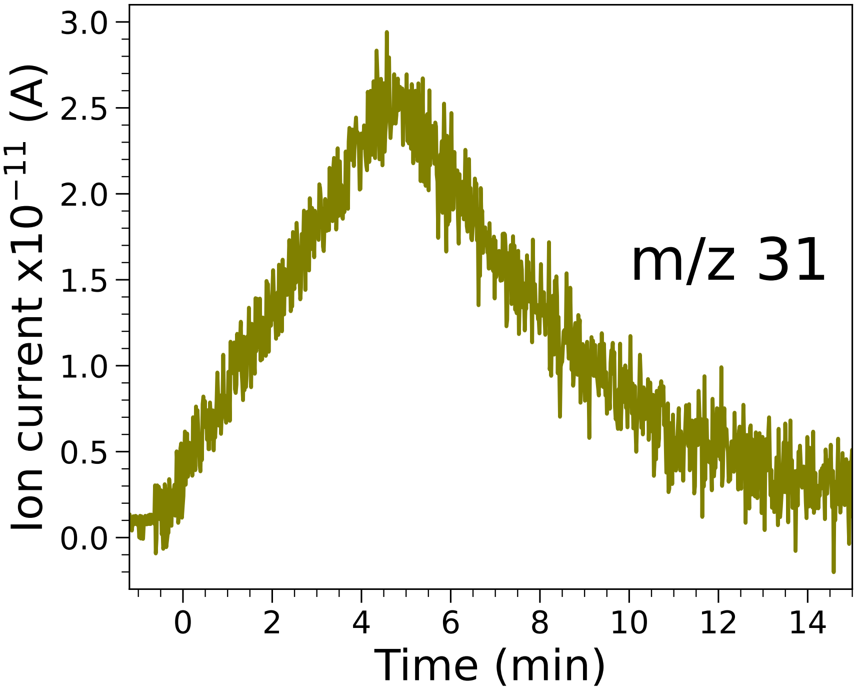

Figure S19: Time evolution of the m/z=31 MS signal collected on MIL-100(Fe) exposed to a mixture of O_2 and CH_4 at 200 °C during the XAS experiment (second reaction cycle).

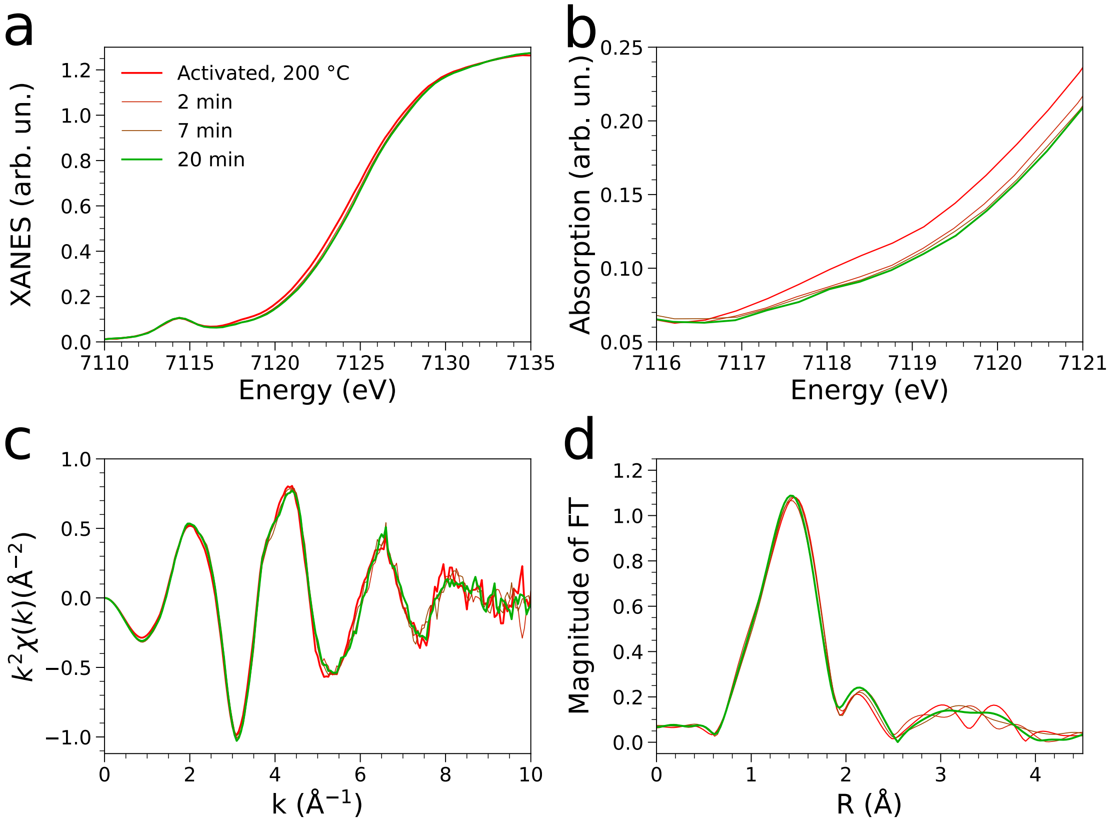

Figure S20: Sequence of Fe K-edge XAS spectra collected during reaction of the activated sample with a mixture of CH_4 and O_2 at 200 °C (second cycle). (a) XANES spectra collected during the reaction. (b) Magnification of the 7117-7122 eV region in the XANES spectra of panel a. (c) Evolution of the EXAFS signal ($k^2\chi(k)$) and (d) non-phase shift corrected FT magnitude of the $k^2\chi(k)$. The color code of all panels is the same as panel a.

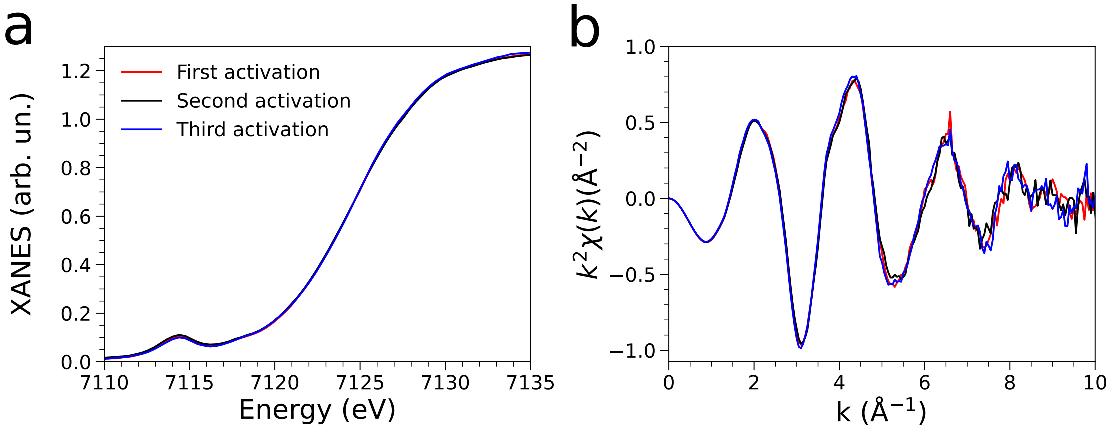

Figure S21: Comparison between Fe K-edge XAS data collected on MIL-100(Fe) activated at 250 °C in He after synthesis (first activation), after a first reaction cycle with CH_4 and O_2 at 200 °C and after a second reaction cycle in the same conditions. (a) XANES data, (b) corresponding $k^2\chi(k)$ EXAFS signals (same color code as panel a).

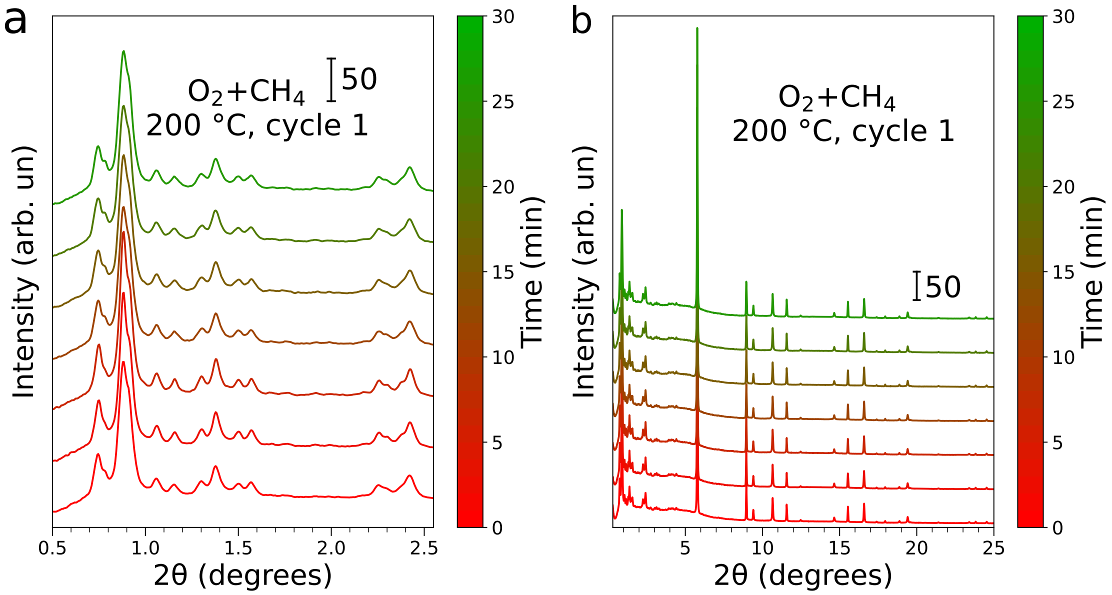

Figure S22: (a) Time evolution of the low angle region in the synchrotron PXRD patterns collected on the MIL-100(Fe) sample during the first reaction cycle with a mixture of CH_4 and O_2 at 200 °C (red, pretreated, to green, post reaction) (a), entire patterns (b). λ=0.3386 Å.

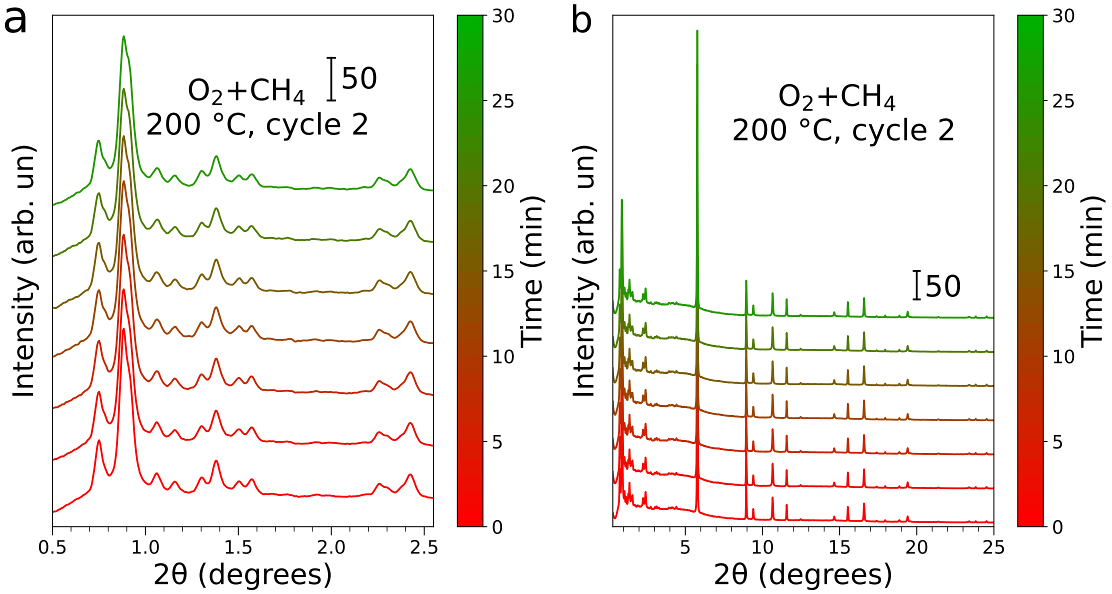

Figure S23: (a) Time evolution of the low angle region in the synchrotron PXRD patterns collected on the MIL-100(Fe) sample during the second reaction cycle with a mixture of CH_4 and O_2 at 200 °C (red, pretreated, to green, post reaction) (a), entire patterns (b). λ=0.3386 Å.

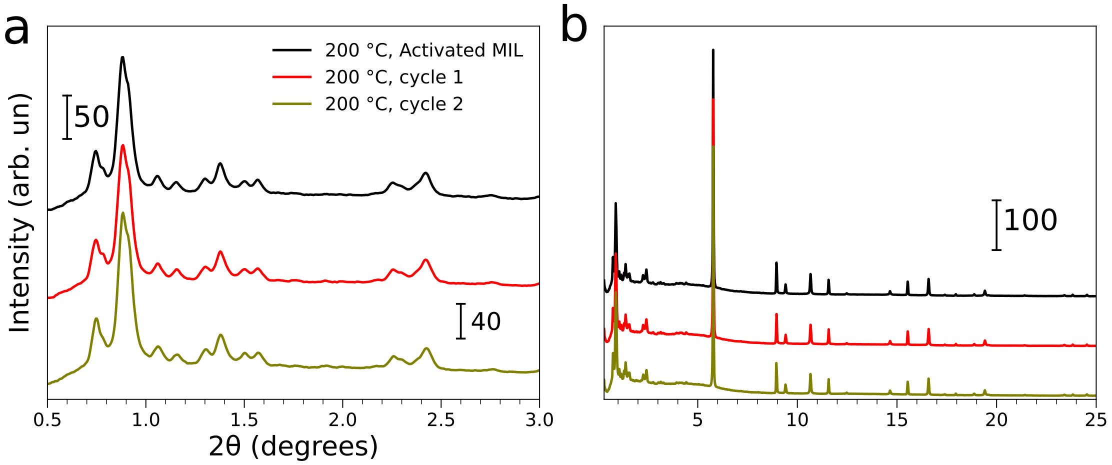

Figure S24: Synchrotron PXRD patterns collected at 200 °C on the MIL-100(Fe) sample after thermal activation at 250 °C in He (black), and after one (red) and two (olive) reaction cycles with CH_4 and O_2.

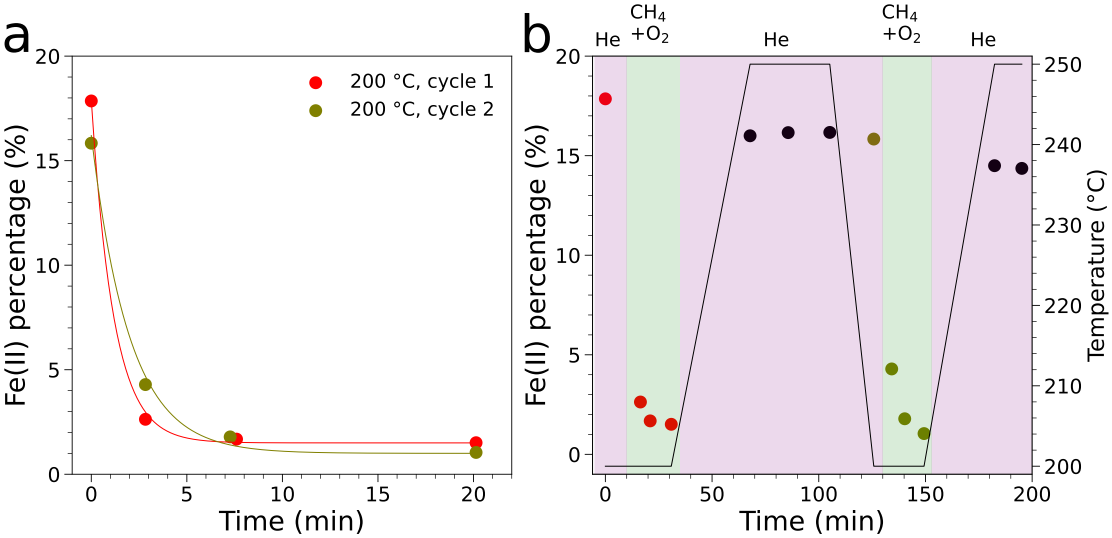

Figure S25: (a) Superimposed time evolution profiles of the Fe(II) percentage in MIL-100(Fe) during MTM oxidation performed at 200 °C for two cycles (red, first cycle, and olive, second cycle). The first point represents the activated MOF prior to the reaction while the solid lines are meant to aid visualization. (b) Sequential time evolution profile of the Fe(II) percentage in MIL-100(Fe) during the first two reaction cycles at 200 °C (red and olive dots, respectively), comprising the reactivation steps at 250 °C in He (black dots). The black curve represents the temperature at each step.

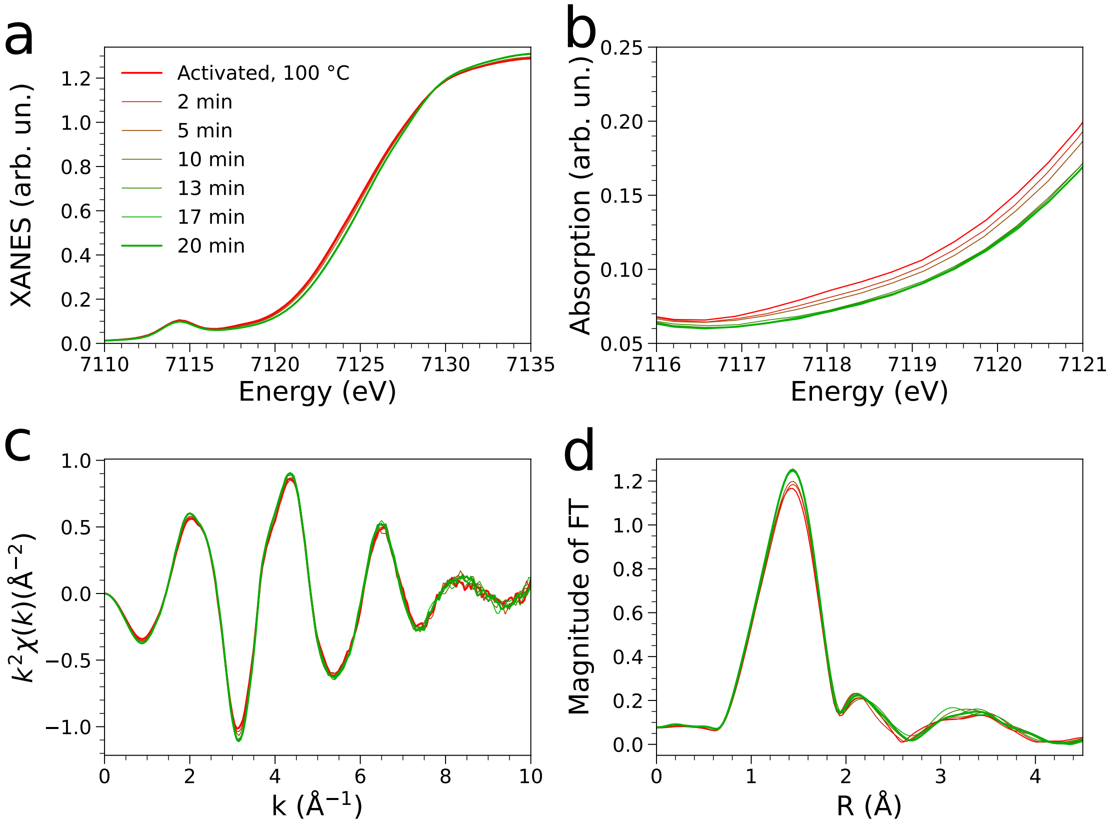

Figure S26: Sequence of Fe K-edge XAS spectra collected during reaction of the activated sample with a mixture of CH_4 and O_2 at 100 °C. (a) XANES spectra collected during the reaction. (b) Magnification of the 7117-7122 eV region in the XANES spectra of panel a. (c) Evolution of the EXAFS signal ($k^2\chi(k)$) and (d) non-phase shift corrected FT magnitude of the $k^2\chi(k)$. The color code of all panels is the same as panel a.

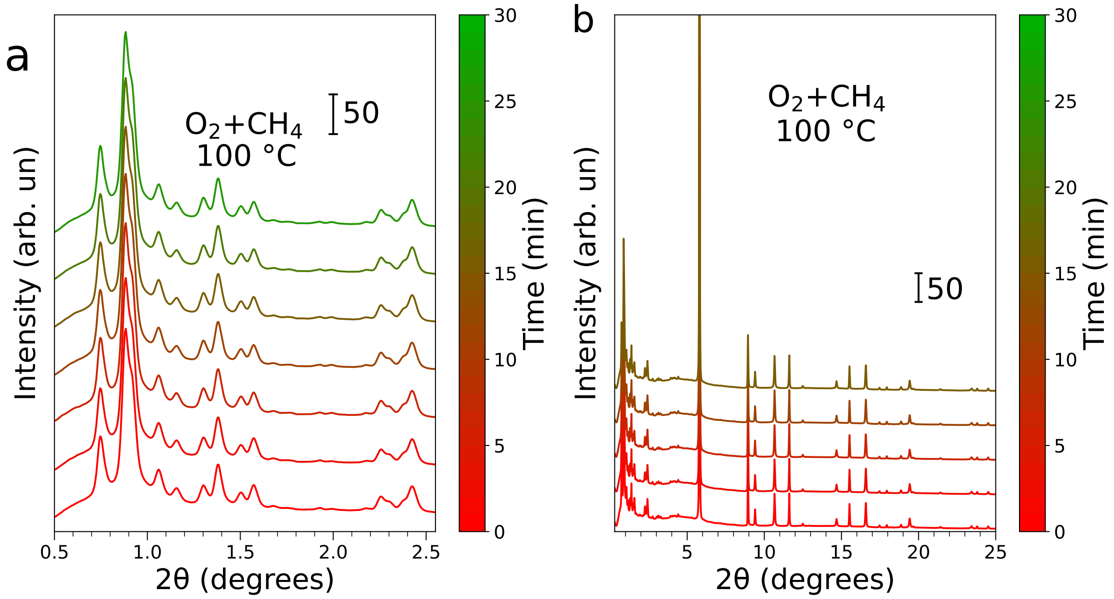

Figure S27: (a) Time evolution of the low angle region in the synchrotron PXRD patterns collected on the MIL-100(Fe) sample during the reaction with a mixture of CH_4 and O_2 at 100 °C (red, pretreated, to green, post reaction) (a), entire patterns (b). λ=0.3386 Å.

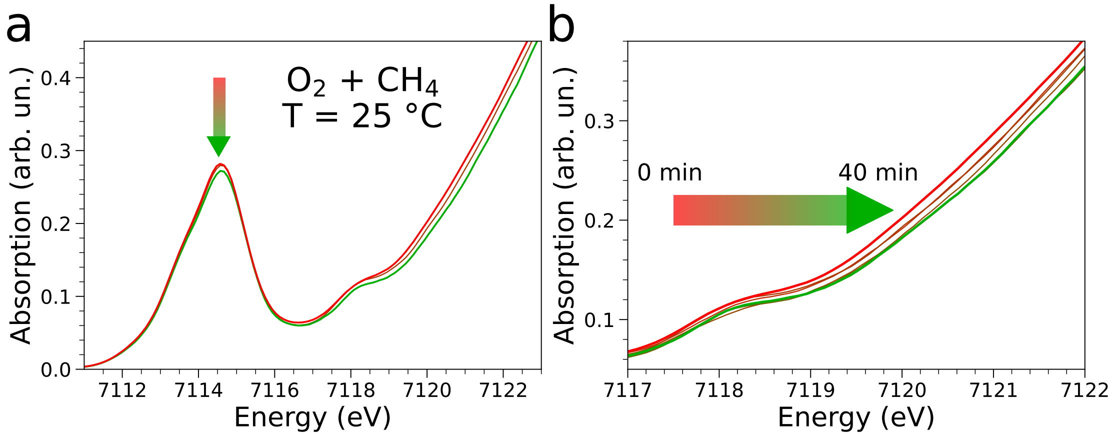

Figure S28: (a) Fe K-edge HERFD-XAS spectra collected on activated MIL-100(Fe) (red), during reaction of the activated sample with a mixture of O_2 and CH_4 at 25 °C (20 min, olive) and after the reaction (40 min, green). (b) Magnification of the 7117-7122 eV region in the sequence of Fe K-edge HERFD-XAS spectra collected on the sample before (bright red) and during the reaction (red, 0 min, to green, 40 min).

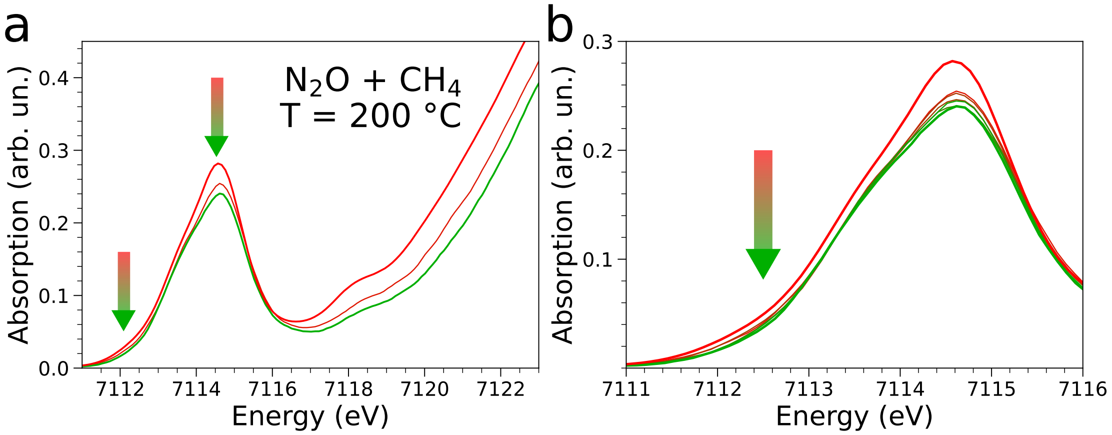

Figure S29: (a) Fe K-edge HERFD-XAS spectra collected on activated MIL-100(Fe) (red), during reaction of the activated sample with a mixture of N_2O and CH_4 at 200 °C (20 min, olive) and after the reaction (40 min, green). (b) Magnification of the 7117-7122 eV region in the sequence of Fe K-edge HERFD-XAS spectra collected on the sample before (bright red) and during the reaction (red, 0 min, to green, 40 min).

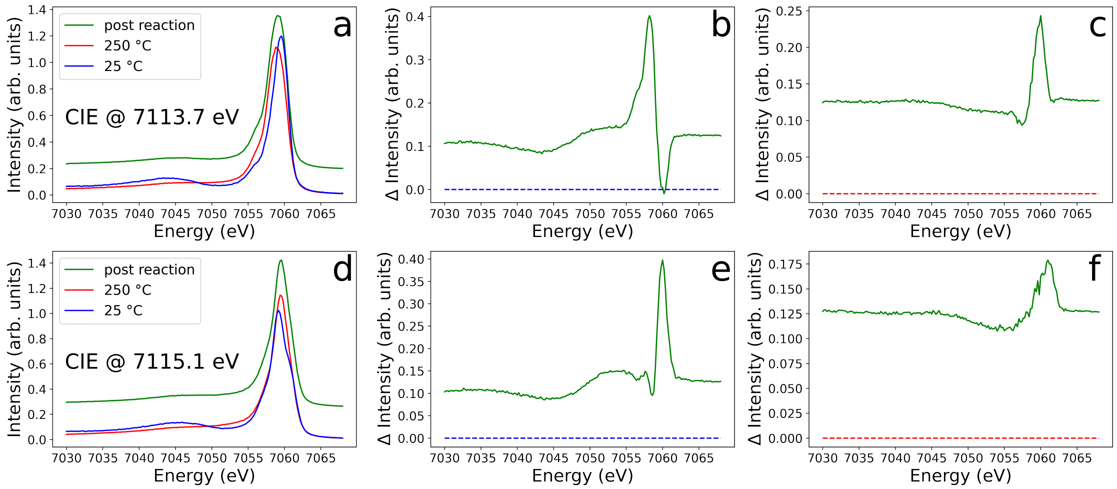

Figure S30: Fe K$\beta_{1,3}$ and Kβ' resonant XES spectra collected on the pristine (blue curve), activated (red curve) MIL-100(Fe) sample and on the sample exposed to N_2O and CH_4 (green) collected at different constant incident energies (CIE) of 7113.7 eV (a) and 7115.1 eV (d). The differences between the post-reaction spectrum and the ones collected on the pristine (b, CIE@7113.7, e, CIE@7115.1) and activated sample (c, CIE@7113.7, f, CIE@7115.1) are also reported.

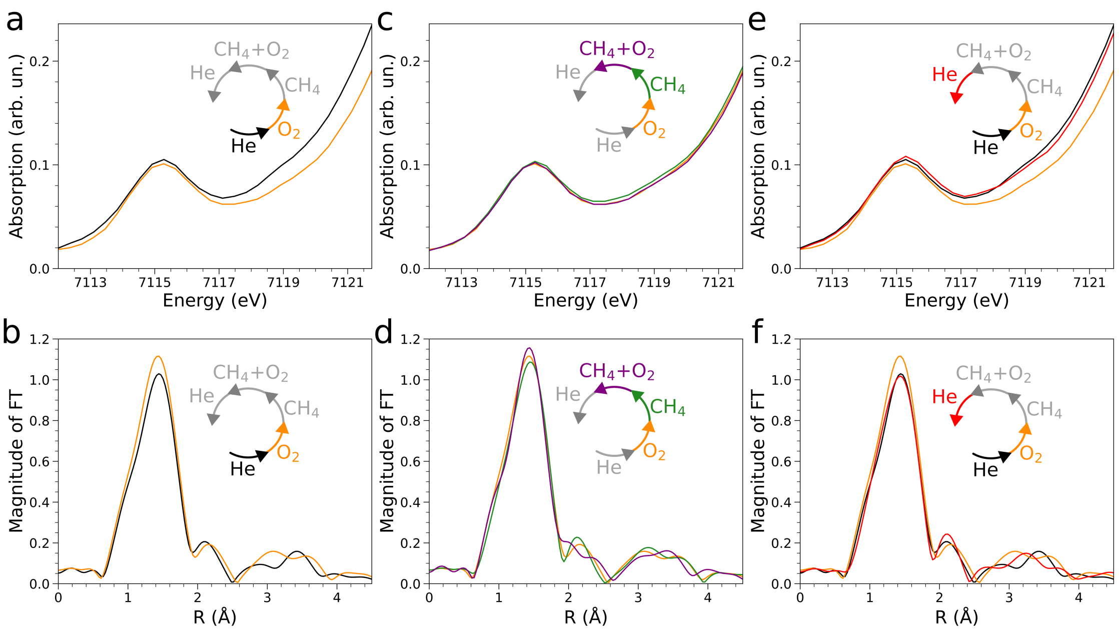

Figure S31: (a, c, e) Sequence of Fe K-edge XAS spectra of MIL-100(Fe) in different conditions: at 200 °C after activation at 250 °C in He flux (black), subsequently at 200 °C in O_2 flux (yellow), in CH_4 flux at 200 °C after O_2 (green), in a mixture of CH_4 and O_2 at 200 °C after CH_4 (purple) and at 250 °C in He after CH_4 and O_2 (red). (b, d, f) FT magnitude of the EXAFS signal recorded in the sequence of panel a. The color code is the same in the two series of panels.

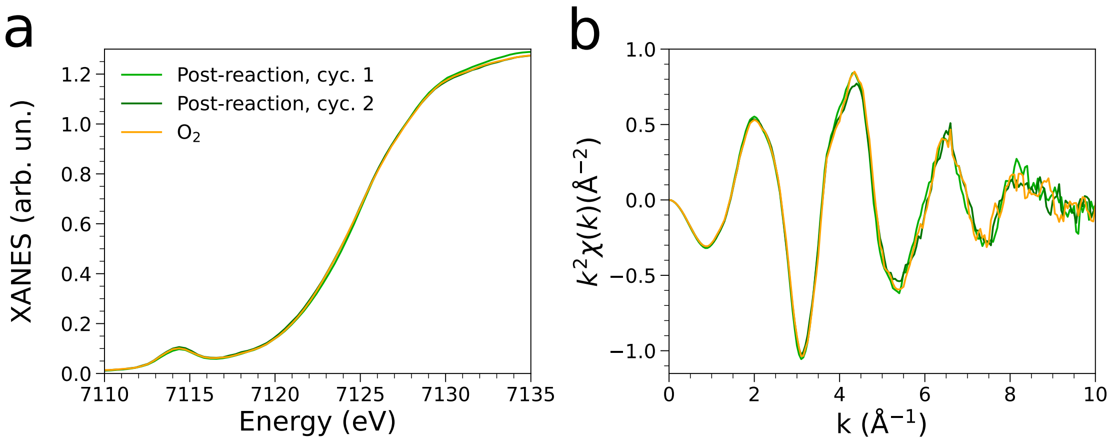

Figure S32: Comparison between Fe K-edge XAS data collected on MIL-100(Fe) after reaction with CH_4 and O_2 at 200 °C (cycle 1 and 2) and after exposure to pure O_2 at 200 °C: (a) XANES data, (b) corresponding $k^2\chi(k)$ EXAFS signals (same color code as panel a).

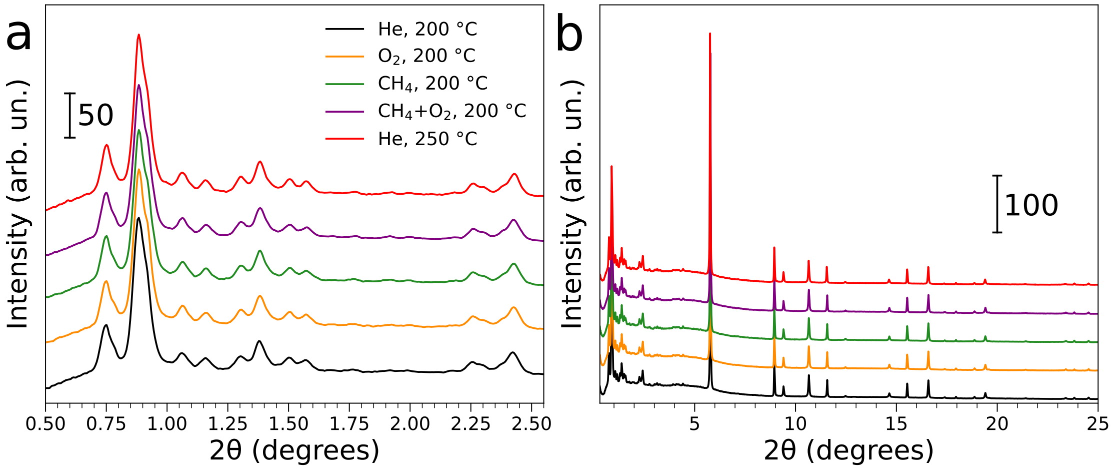

Figure S33: Sequence of synchrotron PXRD patterns collected on MIL-100(Fe) in different conditions: at 200 °C after activation at 250 °C in He flux (black), subsequently at 200 °C in O_2 flux (yellow), in CH_4 flux at 200 °C after O_2 (green), in a mixture of CH_4 and O_2 at 200 °C after CH_4 (purple) and at 250 °C in He after CH_4 and O_2 (red). λ=0.3386 Å.

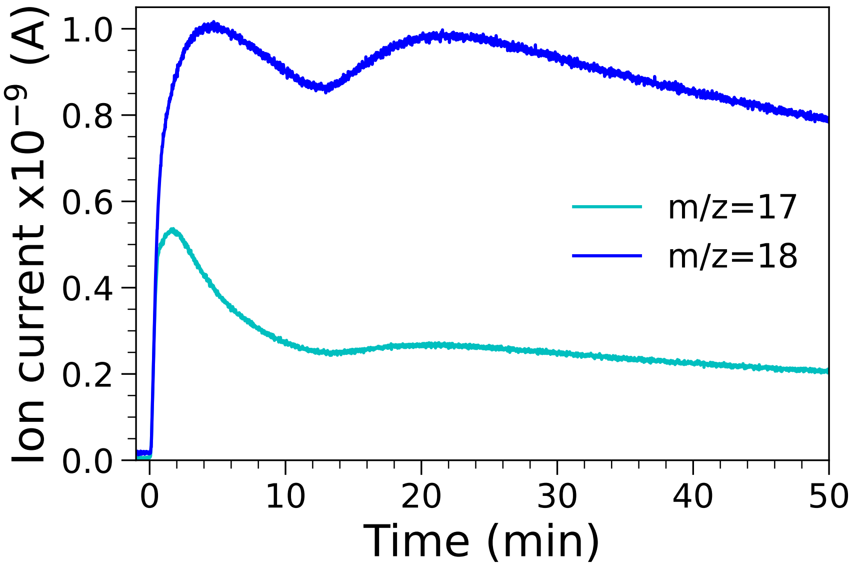

Figure S34: Time evolution of the m/z=17 and m/z=18 MS signals collected during exposure of activated MIL-100(Fe) to O_2 at 200 °C.

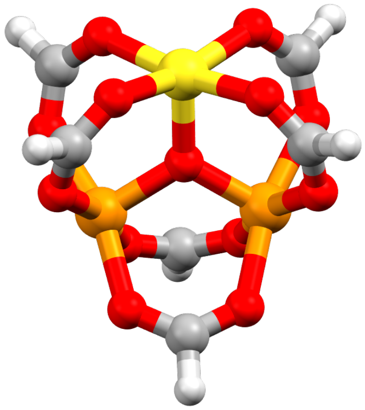

Figure S35: Model employed in the DFT calculations where the BTC ligands have been replaced by formyl groups. Color code: Fe(III), orange, Fe(II), yellow, O, red, C, grey and H, white.

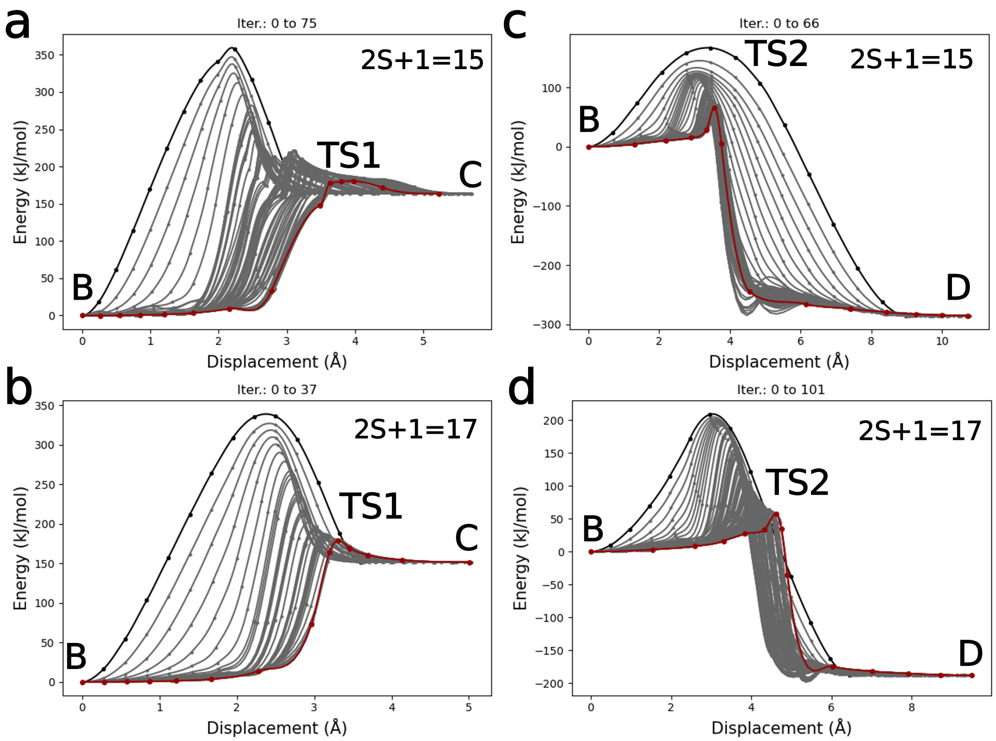

Figure S36: (a) NEB-CI localization of transition state TS1 on the 2S+1=15 spin surface. (b) NEB-CI localization of transition state TS1 on the 2S+1=17 spin surface. (b) NEB-CI localization of transition state TS2 on the 2S+1=15 spin surface. (b) NEB-CI localization of transition state TS2 on the 2S+1=17 spin surface. In all panels, the black and red dots represent the starting and final paths in the optimization, respectively, while the grey dots represent the intermediate paths. A spline interpolation of each path is given by solid lines to aid visualization.

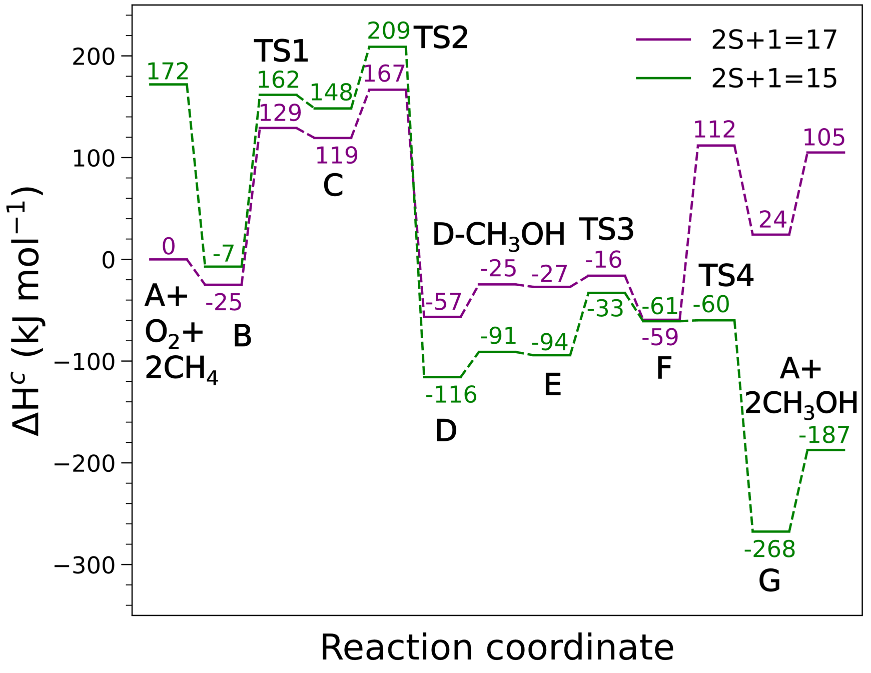

Figure S37: DFT-derived enthalpy diagram of the reaction mechanism comparing the reaction pathway on the 2S+1=15 and 2S+1=17 spin surfaces.

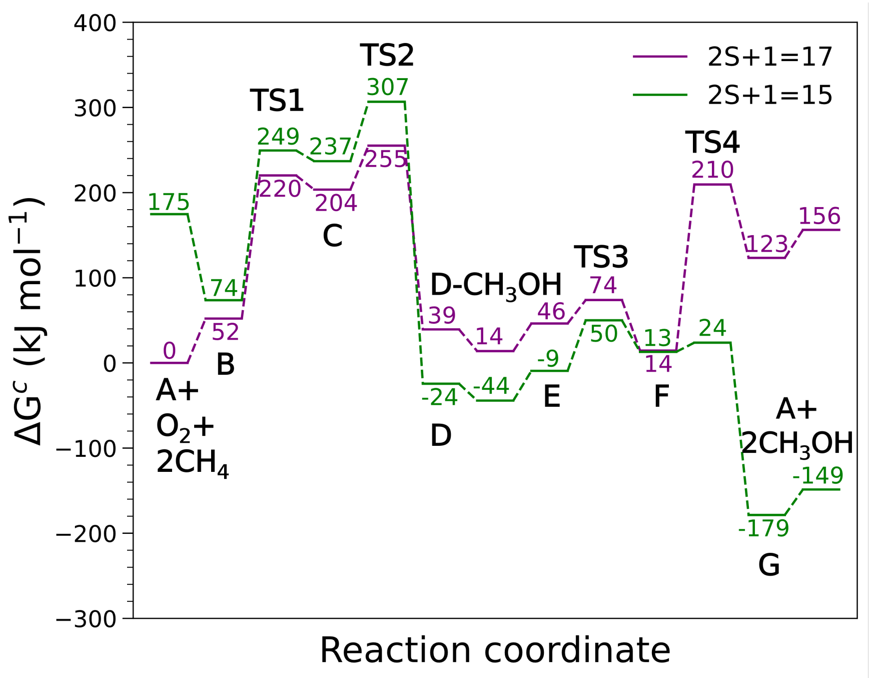

Figure S38: DFT-derived Gibbs free energy diagram of the reaction mechanism comparing the reaction pathway on the 2S+1=15 and 2S+1=17 spin surfaces.

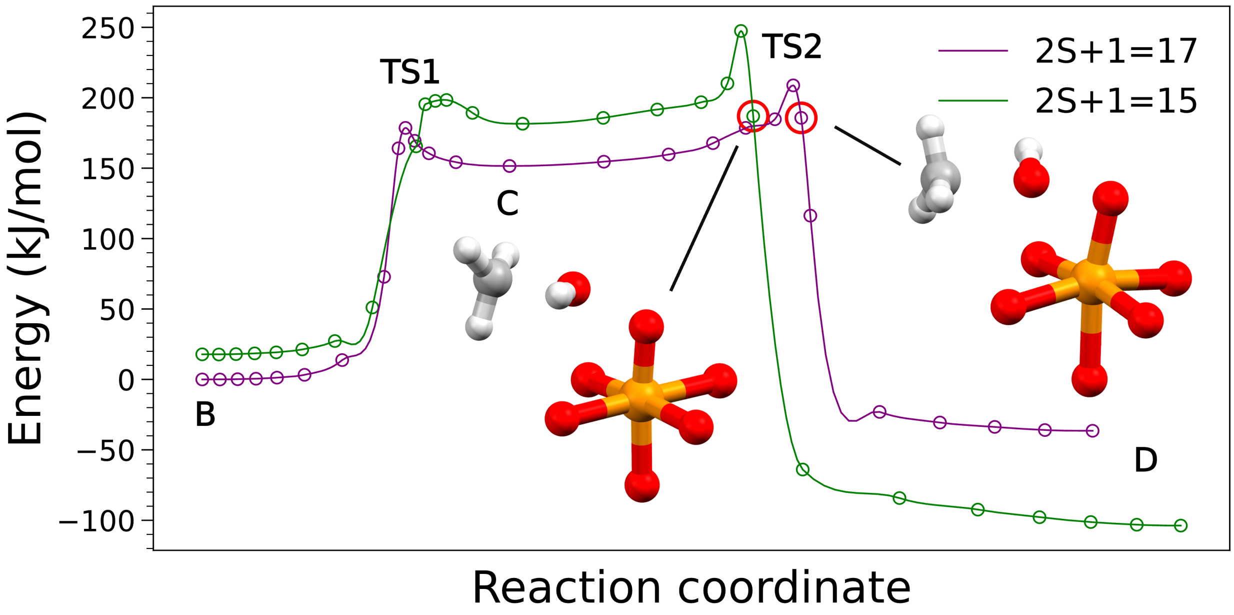

Figure S39: NEB-CI paths connecting intermediates B and D through intermediate C and transition states TS1-TS2 on the 2S+1=15 and 2S+1=17 spin surfaces. The two structures possessing similar electronic energy (image 18 on the 2S+1=15 surface and image 19 on the 2S+1=17 spin surface) are depicted in the image. Color code: orange, Fe(III), red, oxygen, grey, carbon and white, hydrogen). Only the first-neighbor oxygen atoms of the Fe center and the species formed during the reaction have been depicted for the sake of clarity.

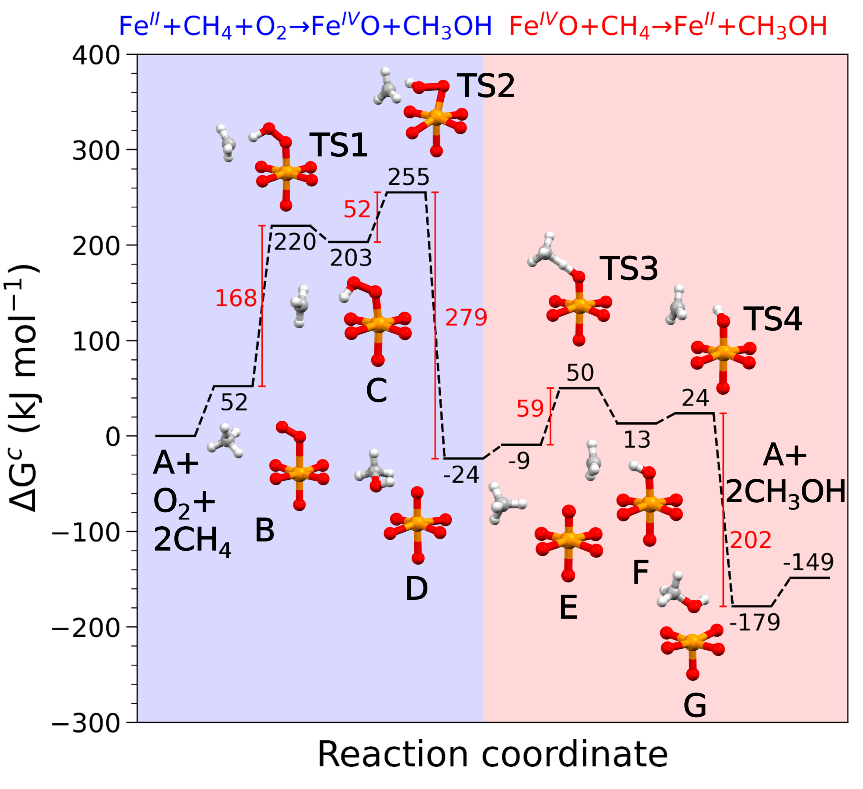

Figure S40: Lowest-Gibbs free energy reaction pathway of the reaction mechanism calculated by DFT. The separated reagents in their ground state set the zero of the scale. To compare the Gibbs free energy of the two reaction stages, the Gibbs free energy of one CH_4 molecule has been added to the Gibbs free energy of species B, TS1, C, TS2 and D. The same has been done with the Gibbs free energy of one CH_3OH molecule for species E, TS3, F, TS4 and G. The structures optimized in the calculations are depicted in the image (color code: orange, Fe(III), red, oxygen, grey, carbon and white, hydrogen). Only the first-neighbor oxygen atoms of the Fe center and the species formed during the reaction have been depicted for the sake of clarity.

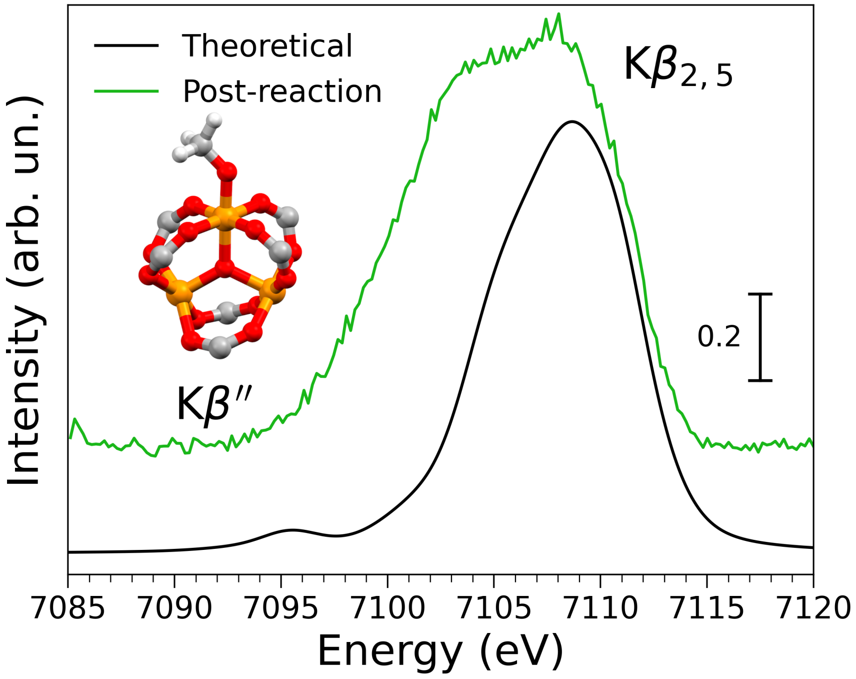

Figure S41: Comparison between the experimental Fe K$\beta_{2,5}$ and Kβ'' VtC-XES spectrum measured on the MIL-100(Fe) sample after the MTM reaction at 200 °C and the theoretical calculation performed using the Fe(III)$_3$O(OCH$_3$) cluster depicted in the panel (color code: Fe(III), orange, O, red, C, grey and H, white). The phenyl groups of the BTC ligands have been omitted for clarity.

5 Supplementary Tables (Tables S1 - S7)

Table S1: Temperature evolution of the refined unit cell parameters for MIL-100(Fe) during thermal treatment (T = 303-583 K) and subsequent cooling (back to 303 K).

**Temperature**	**a axis** (Å)	**Space Group**	**Volume** ($Å^3$)	$\mathbf{R}_{wp}$
303	73.20(4)	$Fd\bar{3}m$	392204(71)	3.73
333	73.16(4)	$Fd\bar{3}m$	391576(67)	3.60
363	73.17(4)	$Fd\bar{3}m$	391713(57)	3.48
393	73.10(4)	$Fd\bar{3}m$	390618(66)	3.59
413	73.01(4)	$Fd\bar{3}m$	389203(64)	3.54
433	72.93(4)	$Fd\bar{3}m$	387935(64)	3.57
443	72.94(4)	$Fd\bar{3}m$	388069(64)	3.59
453	72.90(4)	$Fd\bar{3}m$	387347(68)	3.47
463	72.88(4)	$Fd\bar{3}m$	387114(67)	3.59
473	72.83(4)	$Fd\bar{3}m$	386306(70)	3.58
483	72.80(4)	$Fd\bar{3}m$	385835(67)	3.56
503	72.76(4)	$Fd\bar{3}m$	385144(66)	3.55
523	72.73(4)	$Fd\bar{3}m$	384712(74)	3.55
543	72.72(5)	$Fd\bar{3}m$	384524(77)	3.53
563	72.72(5)	$Fd\bar{3}m$	384619(73)	3.58
583	72.68(5)	$Fd\bar{3}m$	383943(81)	3.50
303	73.23(4)	$Fd\bar{3}m$	392731(69)	3.45

Table S2: Absolute electronic energy, enthalpy and Gibbs free energy values (atomic units) for all DFT-optimized intermediates and transition state structures in the reaction mechanism, calculated on each spin surface.

**Intermediate**	**2S+1**	**E (a. u.)**	**H^0 (a. u.)**	**G^0 (a. u.)**
A	15	-5002.06669402	-5001.89173051	-5001.97389881
A	17	-5001.95190961	-5001.78033776	-5001.85777628
A+2CH_4+O_2	15 (A), 3 (O_2)	-5233.48685535	-5233.20698269	-5233.35804432
A+2CH_4+O_2	15 (A), 1 (O_2)	-5233.42135252	-5233.14148597	-5233.29150688
B	15	-5192.96674414	-5192.73365527	-5192.83114098
B	17	-5192.97350126	-5192.74044044	-5192.83932889
TS1	15	-5192.89774412	-5192.66956173	-5192.76439639
TS1	17	-5192.90868439	-5192.68243679	-5192.77600823
C	15	-5192.90438906	-5192.67464601	-5192.76915482
C	17	-5192.91564419	-5192.68568676	-5192.78184965
TS2	15	-5192.87929955	-5192.65171486	-5192.74272502
TS2	17	-5192.89826531	-5192.66788791	-5192.76245712
D	15	-5193.01322283	-5192.77588735	-5192.86935861
D	17	-5192.98752292	-5192.75364536	-5192.84535895
D-CH_3OH	15	-5077.25152288	-5077.07194613	-5077.15532026
D-CH_3OH	17	-5077.22532848	-5077.04666703	-5077.13317631
E	15	-5117.77934807	-5117.54995817	-5117.64161197
E	17	-5117.75386272	-5117.52439713	-5117.62045905
TS3	15	-5117.74873980	-5117.52706229	-5117.61943393
TS3	17	-5117.74170218	-5117.52067558	-5117.61040934
F	15	-5117.76293165	-5117.53731325	-5117.63322499
F	17	-5117.76241125	-5117.53675492	-5117.63262950
TS4	15	-5117.76077852	-5117.53712259	-5117.62924695
TS4	17	-5117.69544835	-5117.47235835	-5117.55915630
G	15	-5117.85073001	-5117.61766047	-5117.70776151
G	17	-5117.73610952	-5117.50652778	-5117.59281452
A+2CH_3OH	15 (A)	-5233.56459500	-5233.27836185	-5233.41464629
A+2CH_3OH	17 (A)	-5233.44981059	-5233.16696910	-5233.29852376

Table S3: Electronic energy, enthalpy and Gibbs free energy variation values (ΔE^c, ΔH^{0c} and ΔG^{0c}, kJ/mol), with respect to the separated reagents as zero of the scale, for all DFT-optimized intermediates and transition state structures in the reaction mechanism on the 2S+1=15 and 2S+1=17 spin surfaces. All reported values have been corrected for BSSE.

**Intermediate**	**2S+1**	**BSSE** (kJ/mol)	**ΔE** (kJ/mol)	**ΔH^{0c}** (kJ/mol)	**ΔG^{0c}** (kJ/mol)
A+2CH_4+O_2	15 (A), 3 (O_2)	-	171.98	171.96	174.69
A+2CH_4+O_2	15 (A), 1 (O_2)	-	0	0	0
B	15	0.89	-12.75	-7.13	73.65
B	17	0.84	-30.54	-24.99	52.10
TS1	15	1.48	168.99	161.73	249.47
TS1	17	2.69	141.48	129.14	220.19
C	15	1.43	151.50	148.33	236.92
C	17	1.35	121.87	119.27	203.52
TS2	15	1.82	217.77	208.94	306.71
TS2	17	2.10	168.25	166.75	255.18
D	15	3.15	-132.52	-115.75	-24.44
D	17	3.93	-64.26	-56.58	39.35
D-CH_3OH	15	-	-102.20	-91.01	-44.22
D-CH_3OH	17	-	-33.42	-24.64	13.92
E	15	1.02	-107.79	-94.26	-9.32
E	17	1.10	-40.80	-27.07	46.29
TS3	15	2.15	-26.30	-33.02	50.03
TS3	17	2.31	-7.66	-16.09	73.89
F	15	1.29	-64.42	-60.80	12.96
F	17	1.26	-63.08	-59.36	14.50
TS4	15	1.67	-58.38	-59.91	23.79
TS4	17	3.41	114.88	111.87	209.55
G	15	5.44	-290.78	-267.59	-178.58
G	17	5.58	10.29	24.32	123.35
A+2CH_3OH	15 (A)	-	-204.11	-187.41	-148.61
A+2CH_3OH	17 (A)	-	97.26	105.06	156.27

Table S4: Absolute DFT electronic energy, enthalpy and Gibbs free energy values (atomic units) calculated for the desorption of the CH_3 radical. Structures have been obtained by taking the optimized structures of intermediates C and F, used as the zero for the scale in each comparison, and separating the $\cdot CH_3$ radical.

**Structure**	**2S+1**	**E** **(a. u.)**	**H^0** **(a. u.)**	**G^0** **(a. u.)**
C-CH_3 (+CH_3)	16	-5153.06151372	-5152.86609052	-5152.86609052
F-CH_3 (+CH_3)	16	-5077.91185982	-5077.72175982	-5077.80824391

Table S5: DFT electronic energy, enthalpy and Gibbs free energy variation values (ΔE^c, ΔH^{0c} and ΔG^{0c}, kJ/mol) calculated for the desorption of the CH_3 radical. Calculations have been performed according to equation 1.

**Structure**	**2S+1**	**ΔE** (kJ/mol)	**ΔH⁰ᶜ** (kJ/mol)	**ΔG⁰ᶜ** (kJ/mol)
C-CH_3 (+CH_3)	16	-23.54	-20.46	27.00
F-CH_3 (+CH_3)	16	-15.58	-9.92	28.88

Table S6: Absolute DFT electronic energy, enthalpy and Gibbs free energy values (atomic units) for image 18 of the 2S+1=15 and image 19 of the 2S+1=17 NEB-CI paths connecting intermediates B and D.

**Image number**	**2S+1**	**E (a. u.)**	**H^0 (a. u.)**	**G^0 (a. u.)**
18	15	-5192.90232656	-5192.67445902	-5192.75956420
19	17	-5192.90252146	-5192.67388288	-5192.76344516

Table S7: DFT electronic energy, enthalpy and Gibbs free energy variation values (ΔE^c, ΔH^{0c} and ΔG^{0c}, kJ/mol) for image 18 of the 2S+1=15 and image 19 of the 2S+1=17 NEB-CI paths connecting intermediates B and D. Image 18 of the 2S+1=15 spin surface sets the zero of the scale.

**Image number**	**2S+1**	**BSSE (kJ/mol)**	**ΔE** (kJ/mol)	**ΔH^{0c}** (kJ/mol)	**ΔG^{0c}** (kJ/mol)
18	15	2.31	0.00	0.00	0.00
19	17	2.25	-0.57	1.45	-10.25

References

(S1) Tan, F.; Liu, M.; Li, K.; Wang, Y.; Wang, J.; Guo, X.; Zhang, G.; Song, C. Facile Synthesis of Size-Controlled MIL-100(Fe) with Excellent Adsorption Capacity for Methylene Blue. *Chem. Eng. J.* **2015**, *281*, 360–367.

(S2) Dhakshinamoorthy, A.; Alvaro, M.; Hwang, Y. K.; Seo, Y.-K.; Corma, A.; Garcia, H. Intracrystalline diffusion in Metal Organic Framework During Heterogeneous Catalysis: Influence of Particle Size on the Activity of MIL-100(Fe) for Oxidation Reactions. *Dalton Trans.* **2011**, *40*, 10719–10724.

(S3) Shi, J.; Hei, S.; Liu, H.; Fu, Y.; Zhang, F.; Zhong, Y.; Zhu, W. Synthesis of MIL-100(Fe) at Low Temperature and Atmospheric Pressure. *J Chem* **2013**, *2013*.

(S4) Hall, J. N.; Bollini, P. Low-temperature, Ambient Pressure Oxidation of Methane to Methanol over Every Tri–Iron Node in a Metal–Organic Framework Material. *Chem. - Eur. J.* **2020**, *26*, 16639–16643.

(S5) Souza, B. E.; Möslein, A. F.; Titov, K.; Taylor, J. D.; Rudic, S.; Tan, J.-C. Green Reconstruction of MIL-100(Fe) In Water for High Crystallinity and Enhanced Guest Encapsulation. *ACS Sustainable Chem. Eng.* **2020**, *8*, 8247–8255.

(S6) Le Bail, A.; Duroy, H.; Fourquet, J. L. Ab-initio Structure Determination of $LiSbWO_6$ by X-ray Powder Diffraction. *Mater. Res. Bull.* **1988**, *23*, 447–452.

(S7) Coelho, A. A. TOPAS-Academic V6. 2016.

(S8) Coelho, A. A. TOPAS and TOPAS-Academic: an Optimization Program Integrating Computer Algebra and Crystallographic Objects Written in C++. *J. Appl. Crystallogr.* **2018**, *51*, 210–218.

(S9) Cheary, R. W.; Coelho, A. A Fundamental Parameters Approach to X-ray Line–Profile Fitting. *J. Appl. Crystallogr.* **1992**, *25*, 109–121.

(S10) Glatzel, P.; Harris, A.; Marion, P.; Sikora, M.; Weng, T.-C.; Guilloud, C.; Lafuerza, S.; Rovezzi, M.; Detlefs, B.; Ducotté, L. The Five-Analyzer Point-to-Point Scanning Crystal Spectrometer at ESRF ID26. *J. Synchr. Rad.* **2021**, *28*, 362–371.

(S11) Savitzky, A.; Golay, M. J. Smoothing and Differentiation of Data by Simplified Least squares procedures. *Anal. Chem.* **1964**, *36*, 1627–1639.

(S12) Abdala, P. M.; Mauroy, H.; Van Beek, W. A Large–Area CMOS Detector for High-Energy Synchrotron Powder Diffraction and Total Scattering Experiments. *J. Appl. Crystallogr.* **2014**, *47*, 449–457.

(S13) Kieffer, J.; Wright, J. PyFAI: a Python Library for High Performance Azimuthal Integration on GPU. *Powder Diffr.* **2013**, *28*, S339–S350.

(S14) Martini, A.; Guda, S.; Guda, A.; Smolentsev, G.; Algasov, A.; Usoltsev, O.; Soldatov, M.; Bugaev, A.; Rusalev, Y.; Lamberti, C.; Soldatov, A. PyFitit: The Software for Quantitative Analysis of XANES Spectra Using Machine-Learning Algorithms. *Comput. Phys. Comm.* **2019**, 107064.

(S15) Joly, Y. X-ray Absorption Near-Edge Structure Calculations Beyond the Muffin-Tin Approximation. *Phys. Rev. B* **2001**, *63*, 125120.

(S16) Hedin, L.; Lundqvist, B. I. Explicit Local Exchange-Correlation Potentials. *Journal of Physics C: Solid State Physics* **1971**, *4*, 2064.

(S17) Bunău, O.; Joly, Y. Self-Consistent Aspects of X-ray Absorption Calculations. *J. Phys: Condes. Matter* **2009**, *21*, 345501.

(S18) Guda, S. A.; Guda, A. A.; Soldatov, M. A.; Lomachenko, K. A.; Bugaev, A. L.; Lamberti, C.; Gawelda, W.; Bressler, C.; Smolentsev, G.; Soldatov, A. V.; Joly, Y. Optimized Finite Difference Method for the Full-Potential XANES Simulations: Ap-

plication to Molecular Adsorption Geometries in MOFs and Metal-Ligand Intersystem Crossing Transients. *J. Chem. Th. Comp.* **2015**, *11*, 4512–4521.

(S19) Horcajada, P.; Surblé, S.; Serre, C.; Hong, D.-Y.; Seo, Y.-K.; Chang, J.-S.; Grenèche, J.-M.; Margiolaki, I.; Férey, G. Synthesis and Catalytic Properties of MIL-100 (Fe), an Iron(III) Carboxylate with Large Pores. *Chem. Commun.* **2007**, 2820–2822.

(S20) Zhao, Y.; Truhlar, D. G. The M06 Suite of Density Functionals for Main Group Thermochemistry, Thermochemical Kinetics, Noncovalent Interactions, Excited States, and Transition Elements: Two New Functionals and Systematic Testing of Four M06-Class Functionals and 12 Other Functionals. *Theor. Chem. Acc.* **2008**, *120*, 215–241.

(S21) Weigend, F.; Ahlrichs, R. Balanced Basis Sets of Split Valence, Triple Zeta Valence and Quadruple Zeta Valence Quality for H to Rn: Design and Assessment of Accuracy. *Phys. Chem. Chem. Phys.* **2005**, *7*, 3297–3305.

(S22) Weigend, F. Accurate Coulomb-Fitting Basis Sets for H to Rn. *Phys. Chem. Chem. Phys.* **2006**, *8*, 1057–1065.

(S23) Neese, F. Software Update: The ORCA Program System—Version 5.0. *Wiley Interdiscip. Rev.: Comput. Mol. Sci.* **2022**, *12*, e1606.

(S24) Vitillo, J. G.; Bhan, A.; Cramer, C. J.; Lu, C. C.; Gagliardi, L. Quantum Chemical Characterization of Structural Single Fe(II) Sites in MIL-Type Metal–Organic Frameworks for the Oxidation of Methane to Methanol and Ethane to Ethanol. *ACS Catal.* **2019**, *9*, 2870–2879.

(S25) Mavrandonakis, A.; Vogiatzis, K. D.; Boese, A. D.; Fink, K.; Heine, T.; Klopper, W. Ab Initio Study of the Adsorption of Small Molecules on Metal–Organic Frameworks with Oxo-Centered Trimetallic Building Units: the Role of the Undercoordinated Metal Ion. *Inorg. Chem.* **2015**, *54*, 8251–8263.

(S26) Simons, M. C.; Vitillo, J. G.; Babucci, M.; Hoffman, A. S.; Boubnov, A.; Beauvais, M. L.; Chen, Z.; Cramer, C. J.; Chapman, K. W.; Bare, S. R.; Gates, B. C.; Lu, C. C.; Gagliardi, L.; Bahn, A. Structure, Dynamics, and Reactivity for Light Alkane Oxidation of Fe(II) Sites Situated in the Nodes of a Metal–Organic Framework. *J. Am. Chem. Soc.* **2019**, *141*, 18142–18151.

(S27) Vitillo, J. G.; Lu, C. C.; Cramer, C. J.; Bhan, A.; Gagliardi, L. Influence of First and Second Coordination Environment on Structural Fe(II) Sites in MIL-101 for C–H Bond Activation in Methane. *ACS Catal.* **2020**, *11*, 579–589.

(S28) Simons, M. C.; Prinslow, S. D.; Babucci, M.; Hoffman, A. S.; Hong, J.; Vitillo, J. G.; Bare, S. R.; Gates, B. C.; Lu, C. C.; Gagliardi, L.; Bahn, A. Beyond Radical Rebound: Methane Oxidation to Methanol Catalyzed by Iron Species in Metal–Organic Framework Nodes. *J. Am. Chem. Soc.* **2021**, *143*, 12165–12174.

(S29) Vitillo, J. G.; Gagliardi, L. Thermal Treatment Effect on CO and NO Adsorption on Fe (II) and Fe (III) Species in Fe_3O-Based MIL-Type Metal–Organic Frameworks: A Density Functional Theory Study. *Inorg. Chem.* **2021**, *60*, 11813–11824.

(S30) Neese, F. Approximate Second–Order SCF Convergence for Spin Unrestricted Wavefunctions. *Chem. Phys. Lett.* **2000**, *325*, 93–98.

(S31) Stoychev, G. L.; Auer, A. A.; Neese, F. Automatic Generation of Auxiliary Basis Sets. *J. Chem. Theory Comput.* **2017**, *13*, 554–562.

(S32) Schwarz, H. Chemistry with Methane: Concepts Rather than Recipes. *Angew. Chem. Int. Ed.* **2011**, *50*, 10096–10115.

(S33) Boys, S. F.; Bernardi, F. The Calculation of Small Molecular Interactions by the Differences of Separate Total Energies. Some Procedures with Reduced Errors. *Mol. Phys.* **1970**, *19*, 553–566.

(S34) Henkelman, G.; Jónsson, H. Improved Tangent Estimate in the Nudged Elastic Band Method for Finding Minimum Energy Paths and Saddle Points. *J. Chem. Phys.* **2000**, *113*, 9978–9985.

(S35) Neese, F. The ORCA Program System. *Wiley Interdiscip. Rev.: Comput. Mol. Sci.* **2012**, *2*, 73–78.

(S36) Ásgeirsson, V.; Birgisson, B. O.; Bjornsson, R.; Becker, U.; Neese, F.; Riplinger, C.; Jónsson, H. Nudged Elastic Band Method for Molecular Reactions Using Energy-Weighted Springs Combined with Eigenvector Following. *J. Chem. Theory Comput.* **2021**, *17*, 4929–4945.

(S37) Henkelman, G.; Uberuaga, B. P.; Jónsson, H. A Climbing Image Nudged Elastic Band Method for Finding Saddle Points and Minimum Energy Paths. *J. Chem. Phys.* **2000**, *113*, 9901–9904.
